# Synthesis and Bioactivities of Novel Piperonylic Acid Derivatives Containing a Sulfonic Acid Ester Moiety

**DOI:** 10.3389/fchem.2022.913003

**Published:** 2022-05-31

**Authors:** Dandan Xie, Xin Hu, Xiaoli Ren, Zaiping Yang

**Affiliations:** ^1^ State Key Laboratory Breeding Base of Green Pesticide and Agricultural Bioengineering, Ministry of Education, Guizhou University, Huaxi District, Guiyang, China; ^2^ Key Laboratory of Green Pesticide and Agricultural Bioengineering, Ministry of Education, Guizhou University, Huaxi District, Guiyang, China; ^3^ School of Biological Sciences, Guizhou Education University, Wudang District, Guiyang, China; ^4^ School of Biologi and Engineering, Guizhou Medical University, Huaxi District, Guiyang, China

**Keywords:** piperonylic acid, sulfonic acid esters, synthesis, antibacterial activities, insecticidal activity

## Abstract

The crop loss caused by bacteria has increased year by year due to the lack of effective control agents. In order to develop efficient, broad-spectrum, and structurally simple agricultural bactericide, the structure of piperonylic acid was modified and a series of novel piperonylic acid derivatives containing a sulfonic acid ester moiety was synthesized. Bioassay results indicated the compounds exhibited significantly antibacterial activities. Among them, compound **41** exhibited excellent antibacterial activities against *Pseudomonas syringae pv. Actinidiae* (Psa), with inhibitory value 99 and 85% at 100 μg/ml and 50 μg/ml, respectively, which was higher than that of thiodiazole-copper (84 and 77%) and bismerthiazol (96 and 78%). In addition, some compounds also showed moderate insecticidal activity against *Spodoptera frugiperda.* The abovementioned results confirm the broadening of the application of piperonylic acid, with reliable support for the development of novel agrochemical bactericide.

## Graphical Abstract

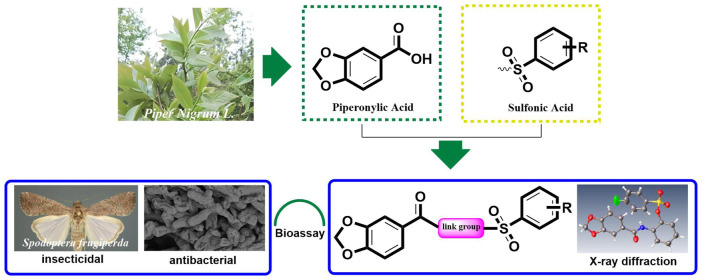

## 1 Introduction

Crop diseases caused by bacteria are considered as the second largest disease in agriculture, second only to fungal diseases, and cause major agricultural losses every year (Abdullahiab et al., 2020; Wang et al., 2021). Although there are some agents widely used to control bacterial diseases, such as bismerthiazol, streptomycin, and copper compounds (Chen M. H. et al., 2021), due to long-term and large-scale use for many years, it not only caused resistance in bacteria but also caused serious environmental problems. Pests were also an important culprit in reducing crop yields. In addition to fed directly on crops, pests also transmitted many viruses and bacteria during migration and feeding. Therefore, it was very necessary to develop an efficient and broad-spectrum agricultural bactericide (Wang et al., 2022).

Due to its characteristics of unique mechanism of action, novel scaffolds, and easy derivation, the natural products have always been a valuable source for lead compounds discovery in agricultural chemistry. Piperonylic acid is an aromatic acid mainly found in black pepper (Moreira et al., 2021). Lots of research results revealed members of the piperonylic acid family had a range of biological activities and were further developed into a commercial drug and widely used in the field of medicine, such as oxolinic acid (Yamazawa et al., 2021; Boycov et al., 2022; Quan et al., 2022), kakuol (Jang et al., 2020; Matsumoto et al., 2020; Sui et al., 2020), and miloxacin (Horie and Nakazawa, 1992; Ueno and Aoki, 1996; Ueno et al., 2001). In addition, piperonylic acid derivatives also showed good activity against bacteria (Umadevi et al., 2013). Sulfonic acid groups are widely used in the field of medicine mainly in the form of sulfonate derivatives. Such as apatinib mesylate (Guo Q. et al., 2020; Chen M. et al., 2021; Kou et al., 2021; Zheng et al., 2021), donafenib tosylate (Wang et al., 2017), and dabrafenib mesylate (Carlos et al., 2015; Liu et al., 2019; Rai et al., 2020) that have been widely used to treat cancer, gemifloxacin mesylate for antibacterial (Chai et al., 2019), and pradefovir mesylate for antiviral (Tuerkova and Zdrazil, 2020). However, many research results revealed that sulfonic acid ester derivatives also had very extensive and excellent biological activities, especially the antibacterial activity was impressive. Guo et al. (2019) and Guo T. et al. (2020) had reported that by splicing a sulfonic acid ester moiety into the backbone of 1,4-pentadien-3-one and chalcone, respectively, the two series of derivatives obtained showed excellent inhibitory activities against bacteria such as *Xanthomonas axonopodis pv. citri* (*Xac*), *Ralstonia solanacearum* (*Rs*), and *Xanthomonas oryzae pv. oryzae* (*Xoo*). Inspired by the results of these studies, the present work aims to incorporate a sulfonic acid ester moiety into the piperonylic acid backbone to synthesize a series of novel derivatives, and further evaluate their antibacterial and insecticidal activity, and hope to obtain piperonylic acid derivatives with good antibacterial activities.

## 2 Experimental

### 2.1 Chemistry

All starting materials and reagents were commercially available and used without further purification, except as indicated. The ^1^H NMR and ^13^C NMR spectra were recorded on a Bruker DPX 400 MHz (Bruker BioSpin GmbH, Rheinstetten, Germany) NMR spectrometer with CDCl_3_ as the solvent. The following abbreviations were used to explain the multiplicities: s, singlet; d, doublet; t, triplet; m, multiplet; and br, broadened. The melting points were determined on a WRX-4 microscope melting point apparatus (YiCe Apparatus & Equipment co., LTD, Shanghai, China). High-resolution mass spectrometry (HRMS) was conducted using a Thermo Scientific Q Exactive (Thermo Fisher Scientific, Massachusetts, America). The X-ray crystallographic data were determined on a D8 Quest X-ray diffractometer (Bruker BioSpin GmbH, Rheinstetten, German).

#### 2.1.1 General Procedures for Preparing Compounds

**SCHEME 1:**
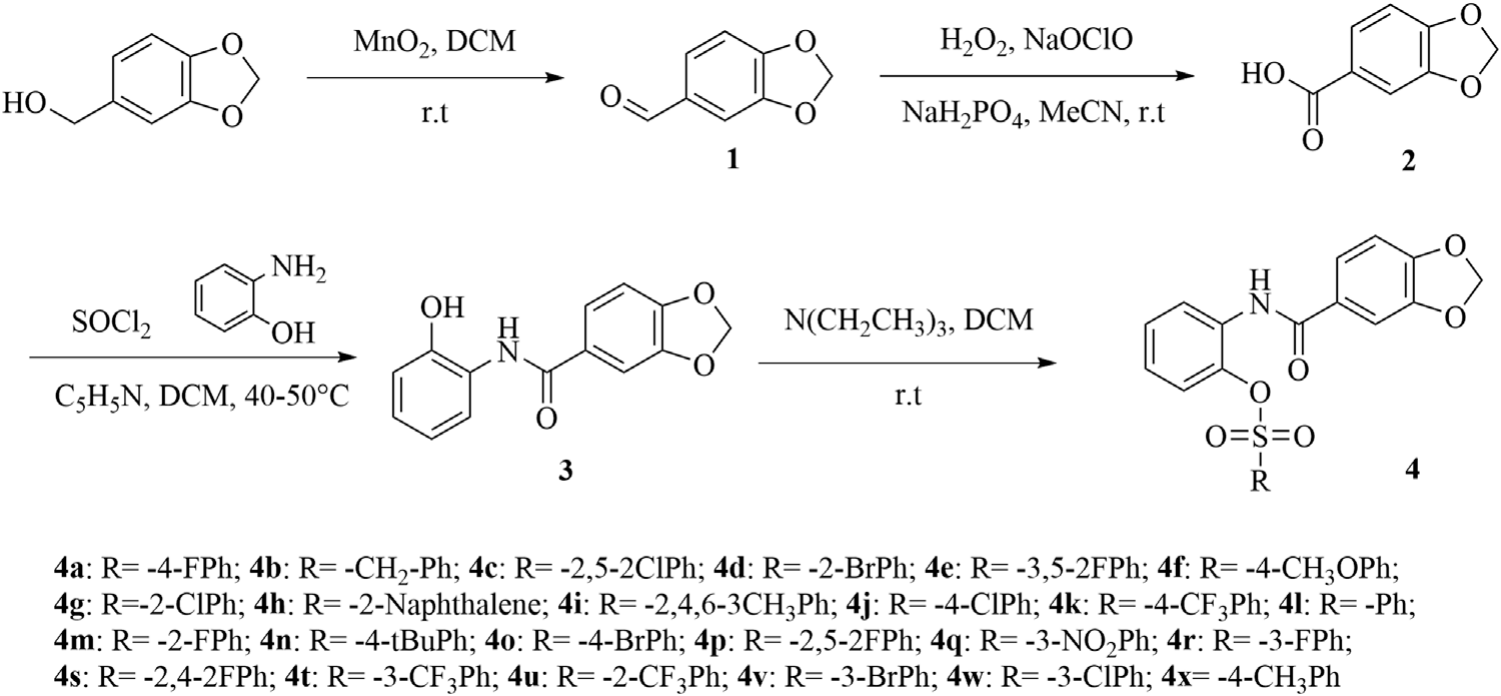
The synthetic route of title compounds 4a-4x.

The synthetic route for the final compounds **4a**–**4x** were depicted in Scheme 1. Intermediates **1–2** were synthesized according to a previously reported method (Dam and Madsen, 2009; Zazeri et al., 2020). Intermediate **3** was prepared according to literature method (Joseph et al., 2019). Target compounds **4a**–**4x** were synthesized by condensation of different sulfonyl chloride which contained different substituent group and intermediate **3** at room temperature condition. Intermediate **2** equivalent of triethylamine was added to the system as a catalyst to neutralize the HCl generated by the reaction so that the reaction can proceed smoothly. After approximately about 4 h, the solvent was removed, and the residue was purified by flash chromatography on silica gel with petroleum *n*-hexane/ethyl acetate (volume ratio 5:1) to obtain the pure product.

##### 2.1.1.1 N-(2-((4-fluorophenyl)sulfonyl)phenyl)benzo[d][1,3]dioxole-5-carboxamide (**4a**)

Light yellow powder, yield 82%. m.p 133.4–134.7°C. ^1^H NMR (400 MHz, CDCl_3_) *δ* 8.32 (dd, *J* = 8.3, 1.6 Hz, 1H, Ph-H), 8.26 (s, 1H, -NH-), 7.87 (dd, *J* = 9.0, 4.9 Hz, 2H, Ph-H), 7.40 (dd, *J* = 8.1, 1.9 Hz, 1H, Ph-H), 7.36–7.29 (m, 2H, Ph-H), 7.21–7.10 (m, 2H, Ph-H), 7.06–7.02 (m, 1.6 Hz, 1H, Ph-H), 6.94–6.87 (m, 2H, Ph-H), 6.09 (s, 2H, -OCH_2_O-). ^13^C NMR (100 MHz, CDCl_3_) *δ* 164.4, 151.0, 148.3, 139.2, 131.5, 131.4, 128.2, 124.5, 123.3, 122.9, 122.0, 117.1, 116.8, 108.3, 107.7, 102.0. HRMS (ESI): calculated for C_20_H_14_FNO_6_S [M + Na]^+^: 438.0526, found: 438.0418.

##### 2.1.1.2 2-(Benzo[d][1,3]dioxole-5-carboxamido)phenyl Phenyl Methanesulfonate (**4b**)

Light yellow powder, yield 80%. m.p 127.5–128.5°C. ^1^H NMR (400 MHz, CDCl_3_) *δ* 7.48 (dd, *J* = 7.8, 1.8 Hz, 1H, -NH-), 7.29 (s, 2H, Ph-H), 7.13–7.01 (m, 6H, Ph-H), 6.95 (dd, *J* = 8.2, 1.5 Hz, 1H, Ph-H), 6.83 (d, *J* = 8.7 Hz, 1H, Ph-H), 6.07 (s, 2H, -OCH_2_O-), 4.65 (s, 2H, -CH_2_-PH). ^13^C NMR (100 MHz, CDCl_3_) *δ* 164.6, 150.8, 148.1, 138.0, 131.8, 130.9, 129.6, 129.2, 128.4, 128.2, 126.8, 124.6, 123.3, 123.0, 122.1, 108.2, 107.8, 101.8, 57.8. HRMS (ESI): calculated for C_21_H_17_NO_6_S [M + Na]^+^: 434.0777, found: 434.0667.

##### 2.1.1.3 2-(Benzo[d][1,3]dioxole-5-carboxamido)phenyl 2,5-Dichlorobenzenesulfonate (**4c**)

Light yellow powder, yield 83%. m.p 149.7–152.7°C. ^1^H NMR (400 MHz, CDCl_3_) *δ* 8.67 (s, 1H, -NH-), 8.30–7.76 (m, 2H, Ph-H), 7.69–7.33 (m, 2H, Ph-H, Ph-H), 7.21–6.74 (m, 6H, Ph-H), 6.08 (s, 2H, -OCH_2_O-). ^13^C NMR (100 MHz, CDCl_3_) *δ* 166.4 151.3, 148.8, 127.2, 125.7, 122.4, 122.3, 120.6, 119.8, 108.3, 107.9, 102.1. HRMS (ESI): calculated for C_20_H_13_Cl_2_NO_6_S [M + Na]^+^: 487.9732, found: 487.9705.

##### 2.1.1.4 2-(Benzo[d][1,3]dioxole-5-carboxamido)phenyl 2-Bromobenzenesulfonate (**4d**)

Light yellow powder, yield 85%. m.p 130.5–132.5°C. ^1^H NMR (400 MHz, CDCl_3_) *δ* 8.58 (s, 1H, -NH-), 8.37 (dd, *J* = 8.3, 1.6 Hz, 1H, Ph-H), 8.11–8.04 (m, 1H, Ph-H), 7.78 (d, *J* = 7.5 Hz, 1H, Ph-H), 7.56–7.46 (m, 3H, Ph-H), 7.41 (d, *J* = 1.8 Hz, 1H, Ph-H), 7.31–7.29 (m, 1H, Ph-H), 7.16–6.98 (m, 2H, Ph-H), 6.90 (d, *J* = 8.1 Hz, 1H, Ph-H), 6.08 (s, 2H, -OCH_2_O-). ^13^C NMR (100 MHz, CDCl_3_) *δ* 164.7, 151.0, 148.2, 138.8, 135.9, 135.6, 132.6, 128.2, 128.0, 124.5, 123.3, 122.8, 122.3, 121.3, 108.2, 108.0, 101.9. HRMS (ESI): calculated for C_20_H_14_BrNO_6_S [M + Na]^+^: 497.9617, found: 497.9614.

##### 2.1.1.5 2-(Benzo[d][1,3]dioxole-5-carboxamido)phenyl 3,5-Difluorobenzenesulfonate (**4e**)

Light yellow powder, yield 81.5%. m.p 152.7–153.8°C. ^1^H NMR (400 MHz, CDCl_3_) *δ* 8.32 (dd, *J* = 8.3, 1.6 Hz, 1H, Ph-H), 8.17 (s, 1H, -NH-), 7.46–7.31 (m, 5H, Ph-H), 7.17–6.82 (m, 4H, Ph-H), 6.09 (s, 2H, -OCH_2_O-). ^13^C NMR (100 MHz, CDCl_3_) *δ* 164.4, 161.6, 151.1, 148.4, 139.1, 131.2, 128.5, 128.1, 124.8, 123.6, 122.6, 121.9, 112.3, 112.0, 110.6, 108.3, 107.7, 102.0. HRMS (ESI): calculated for C_20_H_13_F_2_NO_6_S [M + Na]^+^: 456.0324, found: 456.0319.

##### 2.1.1.6 2-(Benzo[d][1,3]dioxole-5-carboxamido)phenyl 4-Methoxybenzenesulfonate (**4f**)

Light yellow powder, yield 89%. m.p 94.3–95.2°C. ^1^H NMR (400 MHz, CDCl_3_) *δ* 8.34 (s, 1H, -NH-), 8.32 (d, *J* = 2.0 Hz, 1H, Ph-H), 7.75 (d, *J* = 9.0 Hz, 2H, Ph-H), 7.39 (dd, *J* = 8.1, 1.9 Hz, 1H, Ph-H), 7.34–7.28 (m, 2H, Ph-H), 7.01 (dd, *J* = 7.5, 1.6 Hz, 1H, Ph-H), 6.95–6.87 (m, 4H, Ph-H), 6.08 (s, 2H, -OCH_2_O-), 3.86 (s, 3H, -OCH_3_). ^13^C NMR (100 MHz, CDCl_3_) *δ* 164.6, 164.4, 150.9, 139.3, 130.8, 127.9, 125.7, 124.4, 123.1, 122.9, 122.0, 114.7, 108.2, 107.8, 101.9, 55.8. HRMS (ESI): calculated for C_21_H_17_NO_7_S [M + Na]^+^: 450.0618, found: 450.0619.

##### 2.1.1.7 2-(Benzo[d][1,3]dioxole-5-carboxamido)phenyl 2-Chlorobenzenesulfonate (**4g**)

Light yellow powder, yield 88%. m.p 101.8–103.6°C. ^1^H NMR (400 MHz, CDCl_3_) *δ* 8.53 (s, 1H, -NH-), 8.40 (dd, *J* = 8.3, 1.6 Hz, 1H, Ph-H), 7.93–7.90 (m, 1H, Ph-H), 7.73–7.67 (m, 1H, Ph-H), 7.49 (dd, *J* = 8.1, 1.9 Hz, 1H, Ph-H), 7.42 (d, *J* = 1.8 Hz, 1H, Ph-H), 7.36–7.29 (m, 2H, Ph-H), 7.24–7.16 (m, 1H, Ph-H), 7.09–7.05 (m, 1H, Ph-H), 6.91 (d, *J* = 8.2 Hz, 1H, Ph-H), 6.08 (s, 2H, -OCH_2_O-). ^13^C NMR (100 MHz, CDCl_3_) *δ* 164.6, 160.8, 158.2, 151.0, 148.2, 138.4, 137.5, 137.4, 131.5, 131.4, 128.4, 128.2, 124.8, 124.5, 123.0, 122.8, 122.2, 117.7, 117.5, 108.2, 107.9, 101.9. HRMS (ESI): calculated for C_20_H_14_ClNO_6_S [M + Na]^+^: 454.0210, found: 424.0146.

##### 2.1.1.8 2-(Benzo[d][1,3]dioxole-5-carboxamido)phenyl Naphthalene-2-sulfonate (**4h**)

Light yellow powder, yield 83%. m.p 145.6–147.1°C. ^1^H NMR (400 MHz, CDCl_3_) *δ* 8.44 (s, 1H, Ph-H), 8.32 (dd, *J* = 8.3, 1.4 Hz, 1H, Ph-H), 8.22 (s, 1H, -NH-), 7.90 (q, *J* = 8.4 Hz, 3H, Ph-H), 7.75–7.58 (m, 3H, Ph-H), 7.32–7.27 (m, 1H, Ph-H), 7.24–7.16 (m, 2H, Ph-H), 6.77 (d, *J* = 8.1 Hz, 1H, -OCH_2_O-). ^13^C NMR (100 MHz, CDCl_3_) *δ* 164.3, 150.8, 148.1, 139.3, 135.6, 131.8, 131.6, 131.4, 130.5, 129.9, 129.5, 128.1, 128.0, 124.4, 123.1, 123.0, 122.4, 121.7, 108.1, 107.7, 101.8. HRMS (ESI): calculated for C_24_H_17_Cl_2_NO_6_S [M + Na]^+^: 470.0777, found: 470.6777.

##### 2.1.1.9 2-(Benzo[d][1,3]dioxole-5-carboxamido)phenyl 2,4,6-Trimethylbenzenesulfonate (**4i**)

Light yellow powder, yield 84%. m.p 130.5–131.6°C. ^1^H NMR (400 MHz, CDCl_3_) *δ* 8.60 (s, 1H, -NH-), 8.38 (dd, *J* = 8.3, 1.6 Hz, 1H, Ph-H), 7.47 (dd, *J* = 8.1, 1.8 Hz, 1H, Ph-H), 7.41 (d, *J* = 1.9 Hz, 1H, Ph-H), 7.29 (d, *J* = 1.4 Hz, 1H, Ph-H), 7.02–6.84 (m, 4H, Ph-H), 6.67 (dd, *J* = 8.2, 1.5 Hz, 1H, Ph-H), 6.07 (s, 2H, -OCH_2_O-), 2.56 (s, 6H, -2CH_3_), 2.34 (s, 3H, -CH_3_). ^13^C NMR (100 MHz, CDCl_3_) *δ* 164.6, 150.9, 148.2, 144.7, 140.7, 139.1, 132.1, 132.0, 129.8, 128.7, 127.8, 124.3, 123.2, 122.5, 122.0, 108.2, 107.9, 101.8, 22.9, 21.2. HRMS (ESI): calculated for C_23_H_21_NO_6_S [M + Na]^+^: 462.0982, found: 462.0977.

##### 2.1.1.10 2-(Benzo[d][1,3]dioxole-5-carboxamido)phenyl 4-Chlorobenzenesulfonate (**4j**)

Light yellow powder, yield 85%. m.p 134.6–137.0°C. ^1^H NMR (400 MHz, CDCl_3_) *δ* 8.31 (dd, *J* = 8.3, 1.6 Hz, 1H, Ph-H), 8.19 (s, 1H, -NH-), 7.77 (d, *J* = 8.6 Hz, 2H, Ph-H), 7.44 (d, *J* = 8.6 Hz, 2H, Ph-H), 7.37 (dd, *J* = 8.1, 1.9 Hz, 1H, Ph-H), 7.33–7.29 (m, 2H, Ph-H), 7.13–7.02 (m, 1H, Ph-H), 6.95 (dd, *J* = 8.2, 1.5 Hz, 1H, Ph-H), 6.90 (d, *J* = 8.1 Hz, 1H, Ph-H), 6.09 (s, 2H, -2CH_3_). ^13^C NMR (100 MHz, CDCl_3_) *δ* 164.3, 151.1, 148.3, 141.8, 139.2, 133.1, 131.3, 129.9, 129.8, 128.2, 124.6, 123.3, 122.9, 121.9, 108.3, 107.7, 102.0. HRMS (ESI): calculated for C_20_H_14_ClNO_6_S [M + Na]^+^: 454.0122, found: 454.0119.

##### 2.1.1.11 2-(Benzo[d][1,3]dioxole-5-carboxamido)phenyl 4-(Trifluoromethyl)benzenesulfonate (**4k**)

Light yellow powder, yield 82.3%. m.p 120.8–122.5°C. ^1^H NMR (400 MHz, CDCl_3_) *δ* 8.31 (dd, *J* = 8.3, 1.6 Hz, 1H, Ph-H), 8.17 (s, 1H, -NH-), 8.05–7.66 (m, 4H, Ph-H), 7.46–7.29 (m, 3H, Ph-H), 7.10–6.86 (m, 3H, Ph-H), 6.09 (s, 2H, -OCH_2_O-). ^13^C NMR (100 MHz, CDCl_3_) δ 164.2, 151.1, 148.3, 139.2, 138.3, 131.2, 129.0, 128.4, 128.1, 126.6, 126.7, 124.7, 123.6, 122.8, 121.9, 108.2, 107.7, 102.0. HRMS (ESI): calculated for C_21_H_14_F_3_NO_6_S [M + Na]^+^: 488.0386, found: 488.0386.

##### 2.1.1.12 2-(Benzo[d][1,3]dioxole-5-carboxamido)phenyl Benzenesulfonate (**4l**)

Light yellow powder, yield 86.2%. m.p 140.0–140.9°C. ^1^H NMR (400 MHz, CDCl_3_) *δ* 8.34 (dd, *J* = 8.3, 1.6 Hz, 1H, Ph-H), 8.31 (s, 1H, -NH-), 7.86 (dd, *J* = 8.5, 1.3 Hz, 2H, Ph-H), 7.69–7.65 (m, 1H, Ph-H), 7.52–7.48 (m, 2H, Ph-H), 7.40–7.37 (m, 1H, Ph-H), 7.35–7.28 (m, 2H, Ph-H), 7.04–7.02 (m, 1H, Ph-H), 6.95–6.86 (m, 2H, Ph-H), 6.08 (s, 2H, -OCH_2_O-). ^13^C NMR (100 MHz, CDCl_3_) *δ* 164.4, 151.0, 148.2, 139.2, 134.9, 131.4, 129.5, 128.4, 128.0, 124.4, 123.0, 122.9, 122.0, 108.2, 107.8, 101.9. HRMS (ESI): calculated for C_20_H_15_NO_6_S [M + Na]^+^: 420.0512, found: 420.0511.

##### 2.1.1.13 2-(Benzo[d][1,3]dioxole-5-carboxamido)phenyl 2-Fluorobenzenesulfonate (**4m**)

Light yellow powder, yield 80%. m.p 93.7–94.7°C. ^1^H NMR (400 MHz, CDCl_3_) *δ* 8.58 (s, 1H, -NH-), 8.37 (dd, *J* = 8.3, 1.6 Hz, 1H, Ph-H), 8.05 (dd, *J* = 8.0, 1.6 Hz, 1H, Ph-H), 7.62–7.54 (m, 2H, Ph-H), 7.49 (dd, *J* = 8.1, 1.8 Hz, 1H, Ph-H), 7.41 (d, *J* = 1.8 Hz, 1H, Ph-H), 7.36–7.27 (m, 2H, Ph-H), 7.14–7.02 (m, 2H, Ph-H), 6.91 (d, *J* = 8.1 Hz, 1H, Ph-H), 6.08 (s, 2H, Ph-H). ^13^C NMR (100 MHz, CDCl_3_) *δ* 164.7, 151.0, 138.7, 135.7, 132.4, 131.6, 128.2, 127.4, 124.5, 123.2, 122.8, 122.2, 108.2, 108.0, 101.9. HRMS (ESI): Calculated for C_20_H_14_FNO_6_S [M + K]^+^: 454.0163, found: 454.0120.

##### 2.1.1.13 2-(Benzo[d][1,3]dioxole-5-carboxamido)phenyl 4-(tert-butyl) Benzenesulfonate (**4n**)

Light yellow powder, yield 82%. m.p 107.7–109.3°C. ^1^H NMR (400 MHz, CDCl_3_) *δ* 8.36 (dd, *J* = 8.3, 1.6 Hz, 1H, Ph-H), 8.33 (s, 1H, -NH-), 7.64 (dd, *J* = 111.7, 8.7 Hz, 4H, Ph-H), 7.39 (dd, *J* = 8.1, 1.9 Hz, 1H, Ph-H), 7.33–7.27 (m, 2H, Ph-H), 7.06–6.96 (m, 2H, Ph-H), 6.90 (d, *J* = 8.1 Hz, 1H, Ph-H), 6.08 (s, 2H, -OCH_2_O-), 1.31 (s, 9H, -CH_3_ × 3). ^13^C NMR (100 MHz, CDCl_3_) *δ* 164.2, 159.2, 150.9, 148.2, 139.2, 131.5, 128.4, 128.3, 128.0, 126.5, 124.3, 123.0, 122.8, 122.0, 108.2, 107.8, 101.9, 35.4, 30.9. HRMS (ESI): calculated for C_24_H_23_Cl_2_NO_6_S [M + Na]^+^: 476.1144, found: 476.1138.

##### 2.1.1.14 2-(Benzo[d][1,3]dioxole-5-carboxamido)phenyl 4-Bromobenzenesulfonate (**4o**)

Light yellow powder, yield 83%. m.p 122.2–125.1°C. ^1^H NMR (400 MHz, CDCl_3_) *δ* 8.31 (dd, *J* = 8.3, 1.6 Hz, 1H, Ph-H), 8.17 (s, 1H, -NH-), 7.73–7.58 (m, 4H, Ph-H), 7.43–7.30 (m, 3H, Ph-H), 7.06 (td, *J* = 7.9, 1.6 Hz, 1H, Ph-H), 6.96 (dd, *J* = 8.2, 1.5 Hz, 1H, Ph-H), 6.90 (d, *J* = 8.1 Hz, 1H, Ph-H), 6.09 (s, 2H, -OCH_2_O-). ^13^C NMR (100 MHz, 7.30 (m, 3H) *δ* 164.3, 151.1, 148.3, 139.2, 133.6, 132.9, 131.2, 130.5, 129.8, 128.2, 124.6, 123.3, 122.9, 121.9, 108.3, 107.7, 102.0. HRMS (ESI): calculated for C_20_H_14_BrNO_6_S [M + Na]^+^: 497.9617, found: 497.9612.

##### 2.1.1.15 2-(Benzo[d][1,3]dioxole-5-carboxamido)phenyl 2,5-Difluorobenzenesulfonate (**4p**)

Light yellow powder, yield 88%. m.p 109.9–121.6°C. ^1^H NMR (400 MHz, CDCl_3_) *δ* 8.45 (s, 1H, -NH-), 8.39 (dd, *J* = 8.3, 1.6 Hz, 1H, Ph-H), 7.66–7.57 (m, 1H, Ph-H), 7.48 (dd, *J* = 8.1, 1.9 Hz, 1H, Ph-H), 7.44–7.30 (m, 3H, Ph-H), 7.24–7.17 (m, 2H, Ph-H), 7.10 (ddd, *J* = 8.2, 7.4, 1.6 Hz, 1H, Ph-H), 6.92 (d, *J* = 8.1 Hz, 1H, Ph-H), 6.08 (s, 2H, -OCH_2_O-). ^13^C NMR (100 MHz, CDCl_3_) *δ* 164.6, 151.1, 148.3, 138.4, 131.4, 128.5, 124.6, 123.2, 122.7, 122.1, 118.2, 108.3, 107.8, 101.9. HRMS (ESI): calculated for C_20_H_13_F_2_NO_6_S [M + Na]^+^: 456.0323, found: 456.0322.

##### 2.1.1.16 2-(Benzo[d][1,3]dioxole-5-carboxamido)phenyl 3-Nitrobenzenesulfonate (**4q**)

Light yellow powder, yield 81%. m.p 116.2–127.1°C. ^1^H NMR (400 MHz, CDCl_3_) *δ* 8.44 (s, 1H, -NH-), 8.24 (dd, *J* = 8.3, 1.6 Hz, 1H, Ph-H), 7.86 (dd, *J* = 7.8, 1.5 Hz, 1H, Ph-H), 7.74 (td, *J* = 7.7, 1.5 Hz, 1H, Ph-H), 7.66 (td, *J* = 7.7, 1.4 Hz, 1H, Ph-H), 7.53 (dd, *J* = 7.9, 1.4 Hz, 1H, Ph-H), 7.43–7.29 (m, 3H, Ph-H), 7.21 (d, *J* = 1.9 Hz, 1H, Ph-H), 7.15 (ddd, *J* = 8.3, 7.5, 1.6 Hz, 1H, Ph-H), 6.86 (d, *J* = 8.1 Hz, 1H, Ph-H), 6.07 (s, 2H, -OCH_2_O-). ^13^C NMR (100 MHz, CDCl_3_) *δ* 164.6, 151.0, 148.0, 138.9, 135.7, 132.5, 132.1, 131.0, 128.4, 128.2, 127.8, 124.9, 124.7, 123.6, 123.2, 122.4, 108.1, 108.0, 101.9. HRMS (ESI): calculated for C_20_H_14_Cl_2_N_2_O_8_S [M + Na]^+^: 465.0363, found: 465.0362.

##### 2.1.1.17 2-(Benzo[d][1,3]dioxole-5-carboxamido)phenyl 3-Fluorobenzenesulfonate (**4r**)

Light yellow powder, yield 86.6%. m.p 160.0–162.1°C. ^1^H NMR (400 MHz, CDCl_3_) *δ* 8.33 (dd, *J* = 8.3, 1.6 Hz, 1H, Ph-H), 8.23 (s, 1H, -NH-), 7.67–7.62 (m, 1H, Ph-H), 7.59 (ddd, *J* = 7.7, 2.6, 1.7 Hz, 1H, Ph-H), 7.50 (td, *J* = 8.1, 5.1 Hz, 1H, Ph-H), 7.41–7.31 (m, 4H, Ph-H), 7.05 (ddd, *J* = 8.9, 7.4, 1.6 Hz, 1H, Ph-H), 6.96 (dd, *J* = 8.2, 1.5 Hz, 1H, Ph-H), 6.91 (d, *J* = 8.1 Hz, 1H, Ph-H), 6.09 (s, 2H, -OCH_2_O-). ^13^C NMR (100 MHz, CDCl_3_) *δ* 164.4, 151.0, 148.3, 139.1, 131.4, 131.3, 128.3, 128.2, 124.6, 124.3, 123.3, 122.8, 122.4, 122.2, 121.9, 116.0, 115.7, 108.3, 107.7, 101.9. HRMS (ESI): calculated for C_20_H_14_FNO_6_S [M + Na]^+^: 438.0418, found: 438.0419.

##### 2.1.1.18 2-(Benzo[d][1,3]dioxole-5-carboxamido)phenyl 2,4-Difluorobenzenesulfonate (**4s**)

Light yellow powder, yield 83.2%. m.p 95.6–98.4°C. ^1^H NMR (400 MHz, CDCl_3_) *δ* 8.46 (s, 1H, -NH-), 8.38 (dd, *J* = 8.3, 1.6 Hz, 1H, Ph-H), 7.97–7.87 (m, 1H, Ph-H), 7.49 (dd, *J* = 8.1, 1.9 Hz, 1H, Ph-H), 7.41 (d, *J* = 1.9 Hz, 1H, Ph-H), 7.35–7.29 (m, 1H, Ph-H), 7.21–7.14 (m, 1H, Ph-H), 7.12–6.87 (m, 4H, Ph-H), 6.09 (s, 2H, -OCH_2_O-). ^13^C NMR (100 MHz, CDCl_3_) *δ* 164.6, 151.0, 148.3, 138.5, 133.4, 133.3, 131.4, 128.4, 124.6, 123.3, 122.8, 122.1, 112.7, 108.3, 107.8, 106.3, 101.9. HRMS (ESI): calculated for C_20_H_13_F_2_NO_6_S [M + Na]^+^: 456.0324, found: 456.0326.

##### 2.1.1.19 2-(Benzo[d][1,3]dioxole-5-carboxamido)phenyl 3-(Trifluoromethyl)benzenesulfonate (**4t**)

Light yellow powder, yield 84%. m.p 120.9–123.6°C. ^1^H NMR (400 MHz, CDCl_3_) *δ* 8.33 (dd, *J* = 8.3, 1.6 Hz, 1H, Ph-H), 8.23 (s, 1H, -NH-), 8.14 (s, 1H, Ph-H), 8.06–7.90 (m, 2H, Ph-H), 7.67 (t, *J* = 7.9 Hz, 1H, Ph-H), 7.43–7.31 (m, 3H, Ph-H), 7.06 (ddd, *J* = 9.1, 7.4, 1.6 Hz, 1H, Ph-H), 6.94 (dd, *J* = 8.3, 1.5 Hz, 1H, Ph-H), 6.90 (d, *J* = 8.1 Hz, 1H, Ph-H), 6.08 (s, 2H, -OCH_2_O-). ^13^C NMR (100 MHz, CDCl_3_) δ 164.3, 151.1, 148.3, 139.0, 135.9, 131.6, 131.2, 130.4, 128.4, 128.1, 125.5, 124.6, 123.5, 122.7, 121.9, 108.3, 107.7, 102.0. HRMS (ESI): calculated for C_21_H_14_F_3_NO_6_S [M + Na]^+^: 488.0386, found: 488.0396.

##### 2.1.1.20 2-(Benzo[d][1,3]dioxole-5-carboxamido)phenyl 2-(Trifluoromethyl)benzenesulfonate (**4u**)

Light yellow powder, yield 87%. m.p 101.3–105.4°C. ^1^H NMR (400 MHz, CDCl_3_) *δ* 8.36 (s, 1H, -NH-), 8.34 (d, *J* = 4.1 Hz, 1H, Ph-H), 8.13 (d, *J* = 7.9 Hz, 1H, Ph-H), 7.90 (d, *J* = 8.3 Hz, 1H, Ph-H), 7.85–7.69 (m, 2H, Ph-H), 7.42 (dd, *J* = 8.1, 1.8 Hz, 1H, Ph-H), 7.35 (d, *J* = 1.9 Hz, 1H, Ph-H), 7.33–7.28 (m, 1H, Ph-H), 7.08–7.04 (m, 2H, Ph-H), 6.90 (d, *J* = 8.1 Hz, 1H, Ph-H), 6.08 (s, 2H, -OCH_2_O-). ^13^C NMR (100 MHz, CDCl_3_) *δ* 148.2, 138.8, 134.8, 132.9, 132.6, 131.4, 128.3, 124.5, 123.2, 123.0, 122.1, 108.2, 107.8, 101.9. HRMS (ESI): calculated for C_21_H_14_F_3_NO_6_S [M + Na]^+^: 488.0386, found: 488.0388.

##### 2.1.1.21 2-(Benzo[d][1,3]dioxole-5-carboxamido)phenyl 3-Bromobenzenesulfonate (**4v**)

Light yellow powder, yield 84%. m.p 158.0–158.5°C. ^1^H NMR (400 MHz, CDCl_3_) *δ* 8.34 (dd, *J* = 8.3, 1.6 Hz, 1H, Ph-H), 8.20 (s, 1H, -NH-), 8.04 (t, *J* = 1.9 Hz, 1H, Ph-H), 7.80–7.71 (m, 2H, Ph-H), 7.44–7.30 (m, 4H, Ph-H), 7.09–7.05 (m, 1H, Ph-H), 6.98 (dd, *J* = 8.2, 1.6 Hz, 1H, Ph-H), 6.91 (d, *J* = 8.1 Hz, 1H, Ph-H), 6.09 (s, 2H, -OCH_2_O-). ^13^C NMR (100 MHz, CDCl_3_) *δ* 151.1, 148.4, 139.1, 137.9, 136.4, 131.3, 131.1, 130.9, 128.3, 128.2, 127.0, 124.6, 123.5, 123.3, 122.8, 121.9, 108.3, 107.8, 101.9. HRMS (ESI): calculated for C_20_H_14_BrNO_6_S [M + Na]^+^: 497.9617, found: 497.9619.

##### 2.1.1.22 2-(Benzo[d][1,3]dioxole-5-carboxamido)phenyl 3-Chlorobenzenesulfonate (**4w**)

Light yellow powder, yield 86%. m.p 148.4–150.1°C. ^1^H NMR (400 MHz, CDCl_3_) *δ* 8.33 (dd, *J* = 8.2, 1.6 Hz, 1H, Ph-H), 8.20 (s, 1H, -NH-), 7.88 (t, *J* = 1.9 Hz, 1H, Ph-H), 7.74–7.67 (m, 1H, Ph-H), 7.61 (dd, *J* = 2.1, 1.0 Hz, 1H, Ph-H), 7.44 (t, *J* = 8.0 Hz, 1H, Ph-H), 7.39–7.30 (m, 3H, Ph-H), 7.07 (ddd, *J* = 9.0, 7.4, 1.6 Hz, 1H, Ph-H), 6.98 (dd, *J* = 8.2, 1.5 Hz, 1H, Ph-H), 6.91 (d, *J* = 8.1 Hz, 1H, Ph-H), 6.08 (s, 2H, -OCH_2_O-). ^13^C NMR (100 MHz, CDCl_3_) *δ* 164.3, 151.1, 148.3, 139.1, 136.3, 135.9, 135.0, 131.3, 130.7, 128.3, 126.5, 124.6, 123.3, 122.8, 121.9, 108.3, 107.8, 101.9. HRMS (ESI): calculated for C_20_H_14_ClNO_6_S [M + Na]^+^: 454.0168, found: 454.0121.

##### 2.1.1.23 2-(Benzo[d][1,3]dioxole-5-carboxamido)phenyl 4-Methylbenzenesulfonate (**4x**)

Light yellow powder, yield 85%. m.p 130.0–131.1°C. ^1^H NMR (400 MHz, CDCl_3_) *δ* 8.33 (dd, *J* = 8.3, 1.6 Hz, 1H, Ph-H), 8.28 (s, 1H, -NH-), 7.72 (d, *J* = 8.4 Hz, 2H, Ph-H), 7.38 (dd, *J* = 8.1, 1.8 Hz, 1H, Ph-H), 7.32–7.27 (m, 3H, Ph-H), 7.26 (s, 1H, Ph-H), 7.02 (td, *J* = 7.8, 7.4, 1.6 Hz, 1H, Ph-H), 6.94 (dd, *J* = 8.2, 1.5 Hz, 1H, Ph-H), 6.90 (d, *J* = 8.1 Hz, 1H, Ph-H), 6.08 (s, 2H, -OCH_2_O-), 2.42 (s, 3H, -CH_3_). ^13^C NMR (100 MHz, CDCl_3_) *δ* 164.3, 150.9, 148.2, 146.3, 139.2, 131.6, 131.4, 130.1, 128.4, 128.0, 124.4, 123.0, 122.9, 122.0, 108.2, 107.8, 101.9, 21.8. HRMS (ESI): calculated for C_21_H_17_NO_6_S [M + Na]^+^: 434.0669, found: 434.0673.

### 2.2 Antimicrobial Assay

The antimicrobial activity of the derivatives (**4a**–**4x**) was tested using the turbidimeter test, the commercial agricultural bactericide bismerthiazol, thiodiazole-copper and lead compound piperonylic acid used as control. The test compounds were dissolved in 150 μL of dimethylformamide (DMF) and diluted with 0.1% (v/v) Tween-20 to prepare two concentrations of 100 and 50 μg/ml. One milliliter of the liquid sample was added to the 40 ml non-toxic nutrient broth medium (NB: 1.5 g of beef extract, 2.5 g of peptone, 0.5 g of yeast powder, 5.0 g of glucose, and 500 ml of distilled water, pH 7.0–7.2). Then, 40 μL of NB medium containing bacteria was added to 5 ml of solvent NB containing the test compounds or thiodiazole–copper. The inoculated test tubes were incubated at 30 ± 1°C with continuous shaking at 180 rpm for 48 h. The culture growth was monitored spectrophotometrically by measuring the optical density at 600 nm (OD_600_) and expressed as corrected turbidity. The relative inhibition rates Inhibition (%) were calculated as the following equation, where C_tur_ was the corrected turbidity value of bacterial growth on untreated NB and T_tur_ was the corrected turbidity value of bacterial growth on treated NB.

$$\text{Inhibition}\left(\text{\%}\right)=\left({\text{C}}_{\text{tur}}{\;-\text{ T}}_{\text{tur}}\right){\text{/C}}_{\text{tur}}\times 100\text{\%}\text{.}$$

### 2.3 Insecticidal Activity Assay

Divide 20 second-instar larvae of *Spodoptera frugiperda* into 20 small cups and starve for 3–4 h. Cut the fresh corn leaves into small leaf discs of 1 cm × 1 cm with scissors, and then soak them in each test solution for 5 s, and then air dry them naturally. Then put them in a cup with *Spodoptera frugiperda* and keep it under the conditions of temperature of 25 ± 1°C, relative humidity of 60∼70%, and a light-dark cycle of L: D = 14 h: 10 h. Feed normal fresh corn leaf discs after 12 h and record the number of dead insects at 12, 24, and 36 h.

## 3 Results and Discussion

### 3.1 Chemistry

**FIGURE 1:**
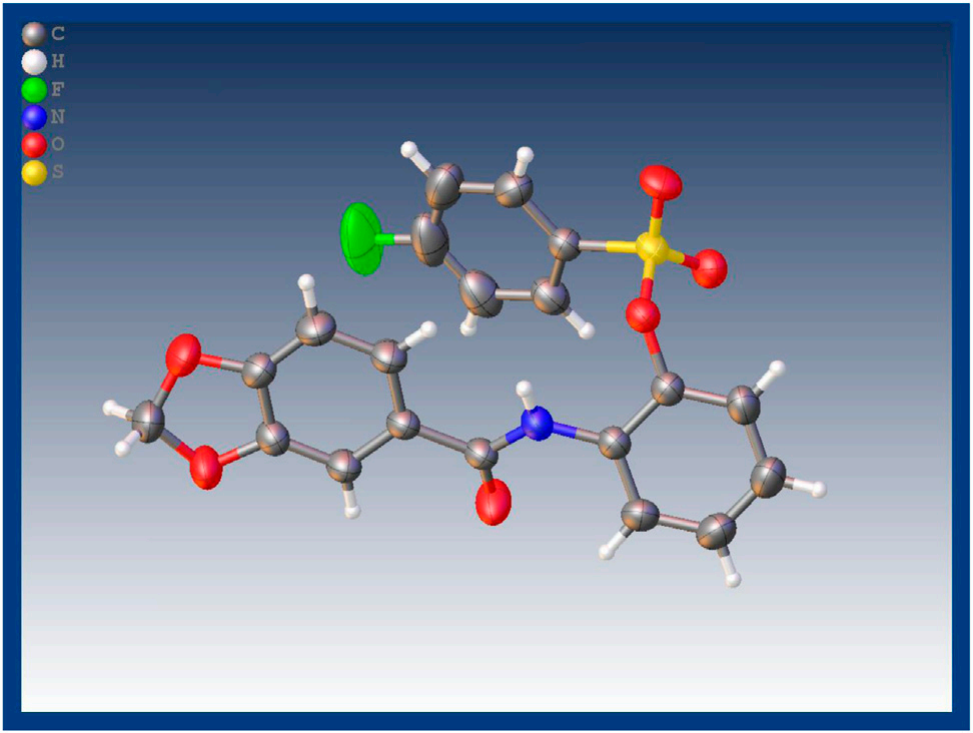
X-ray crystal structure of compound **4a**.

The synthetic route for the target compounds **4a–4x** was shown in Scheme 1. Intermediates **2–3** were prepared according to previously reported procedures, and the yield of all compounds was satisfactory, usually higher than 80%. In the syntheses target compounds of **4a–4x**, the yield when using inorganic base, such as K_2_CO_3_ or KHCO_3_ as catalyst was usually only approximately 30%. When inorganic base was replaced with organic base triethylamine as the catalyst, the yield was considerably greater usually more than 80%. It was worth noting that when the intermediates **3** were synthesized using acid and 2-amino phenol the carboxyl group might have reacted with hydroxyl group to form an ester, or it may have reacted with the amino group to form an amide. These two structures of isomers were difficult to confirm through HRMS or NMR. In order to get the exact structure of the target compound, we used X-ray to confirm the structure of compound **4a**, and the results are show in Figure 1 (CCDC 2131244). Crystal data of **4a** indicated that target compounds were in the form of carbonamide instead of carbonate.

### 3.2 *In Vitro* Antibacterial Activity

Antibacterial activities of target compounds **4a–4x** against agriculturally important pathogenic bacteria *Psa* and *Xoo* were determined *in vitro via* the turbidimetric method, using the commercialized bismerthiazol, thiodiazole-copper, and piperonylic acid as a control agent. The bactericide which was used to make the bioassay was provided by Guizhou Tea Institute, and the results of the bioassay against *Psa* and *Xoo* are shown in Table 1 and indicated that most of the title compounds exhibited good to excellent activities *in vitro*. Compounds **4l**, **4o,** and **4v** showed excellent activities against *Psa* at 100 μg/ml with inhibition rates of 99%, which were higher than those of thiodiazole-copper (84%), bismerthiazol (96%), and lead compound piperonylic acid (59%), respectively. In particular, even at a concentration as low as 50 μg/ml, compound **4l** was found to still possess a pronounced anti-Pas efficacy of 85%. Moreover, compounds **4e**, **4f**, and **4j** exhibited higher activities (i.e., 87, 67 and 76%, respectively) against *Xoo* than that of thiodiazole-copper (60%), bismerthiazol (55%), and piperonylic acid (21%) at 100 μg/ml even at a concentration as low as 50 μg/ml, compound **4e** was found to still possess a pronounced anti-*Xoo* effifcacy of 76%, which was significantly higher than that of control agent. It is worth mentioning that whether for *Psa* or *Xoo*, the activities of almost all target compounds were significantly higher than that of the lead compound piperonylic acid. This indicated that incorporation of a sulfonic acid ester moiety into the piperonylic acid backbone could significantly improve its antibacterial activity.

**TABLE 1:** *In vitro* antibacterial activities of the target compounds **4a**–**4x**.

Compound	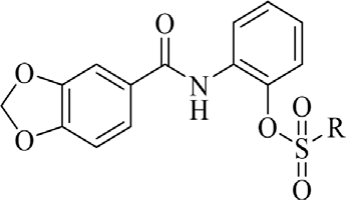	*Pseudomonas syringae pv. actinidiae (Psa)*		*Xanthomonas oryzae pv. oryzae (Xoo)*	
	R	100 μg/ml	50 μg/ml	100 μg/ml	50 μg/ml
**4a**	-4-FPh	76 ± 1.3	68 ± 2.7	52 ± 0.6	45 ± 0.9%
**4b**	-CH_2_-Ph	77 ± 1.2	73 ± 1.2	42 ± 2.2	38 ± 2.3%
**4c**	-2,5-2ClPh	80 ± 0.8	75 ± 1.4	59 ± 1.1	52 ± 1.1%
**4d**	-2-BrPh	96 ± 1.5	80 ± 1.6	38 ± 1.3	30 ± 0.8%
**4e**	-3,5-2BrPh	83 ± 1.8	77 ± 2.4	87 ± 2.8	76 ± 1.5%
**4f**	-4-CH_3_OPh	87 ± 0.7	80 ± 1.0	67 ± 3.6	51 ± 0.9%
**4g**	-2-ClPh	82 ± 1.0	80 ± 1.7	37 ± 1.7	61 ± 0.8%
**4h**	-2-Naphthalene	93 ± 2.1	80 ± 1.4	35 ± 0.5	17 ± 1.2%
**4i**	-2,4,6-3CH_3_Ph	82 ± 1.1	75 ± 2.2	61 ± 1.9	44 ± 1.1%
**4j**	-4-ClPh	90 ± 1.7	71 ± 1.3	76 ± 0.8	43 ± 2.4%
**4k**	-4-CF_3_Ph	88 ± 1.8	72 ± 2.0	53 ± 1.7	39 ± 1.2%
**4l**	-Ph	99 ± 2.0	85 ± 1.3	47 ± 2.7	18 ± 1.6%
**4m**	-2-FPh	94 ± 3.0	78 ± 0.6	45 ± 0.6	13 ± 2.9%
**4n**	-4-tBu Ph	88 ± 2.3	79 ± 3.0	38 ± 1.6	36 ± 1.8%
**4o**	-4-BrPh	99 ± 1.4	81 ± 2.1	59 ± 3.4	54 ± 2.7%
**4p**	-2,5-2FPh	94 ± 1.0	78 ± 1.3	54 ± 2.3	44 ± 1.3%
**4q**	-3-NO_2_Ph	81 ± 1.1	78 ± 2.6	62 ± 1.8	32 ± 2.5%
**4r**	-3-FPh	86 ± 1.4	79 ± 2.9	61 ± 2.0	30 ± 1.3%
**4s**	-2,4-2FPh	91 ± 2.3	89 ± 0.8	64 ± 2.0	42 ± 2.4%
**4t**	-3-CF_3_Ph	87 ± 2.1	78 ± 0.9	47 ± 1.4	9 ± 1.5%
**4u**	-2-CF_3_Ph	81 ± 1.6	78 ± 1.4	44 ± 2.8	12 ± 0.3%
**4v**	-3-BrPh	99 ± 1.1	81 ± 1.6	64 ± 1.8	47 ± 0.4%
**4w**	-3-FPh	79 ± 1.6	75 ± 2.4	54 ± 2.9	30 ± 2.3%
**4x**	-4-CH_3_Ph	83 ± 1.4	78 ± 1.3	29 ± 1.0	8 ± 1.5%
—	Piperonylic acid	59 ± 2.1	47 ± 1.1	21 ± 1.9	13 ± 1.7%
—	Bismerthiazol	96 ± 3.1	78 ± 2.1	55 ± 2.2	53 ± 2.3%
—	Thiodiazole-copper	84 ± 1.8	77 ± 3.1	60 ± 1.9	59 ± 0.6%

### 3.3 Insecticidal Activity Assay Against *Spodoptera frugiperda*

**FIGURE 2:**
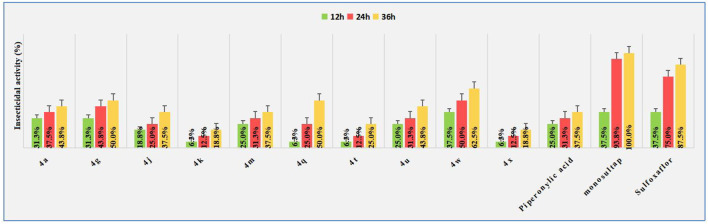
Insecticidal activity against *Spodoptera frugiperda* of title compounds.

In view of the literature which reported that piperonylic acid has certain insecticidal activities, we also evaluated the activity of some title compounds against *Spodoptera frugiperda* at 50 μg/ml, The *Spodoptera frugiperda* used in the biological tests were collected from fields in Luodian County, Guizhou Province, China, and bred in a greenhouse. The pesticidal results are shown in Figure 2. Although most compounds exhibited certain insecticidal effect on *Spodoptera frugiperda*, such as the lethal rate of compound **4g**, **4q**, and **4w** on to the second instar larvae of the insect reached 50.0, 50.0 and 62.5% at the 36 h, respectively, which was significantly higher than the lead structure piperonylic acid (37.5%), but still lower than the commercial insecticide monosultap (100%) and sulfoxaflor (87.5%).

## 4 Conclusion

In summary, in order to seek new efficiency, broad-spectrum, and structure simple agricultural bactericide, a series of novel piperonylic acid derivatives containing a sulfonic acid ester moiety was synthesized. The structures of the title compounds were verified by ^1^H NMR, ^13^C NMR, and HRMS. The bioassay results revealed that these compounds showed good inhibition activity against *Xoo* and *Psa*, and some compounds even exhibited higher antibacterial activity than those of commercial bactericide which are widely used. The title compounds showed weaker activity against *|Spodoptera frugiperda* compared with commercial pesticides. Thus, we recommend these newly designed and synthesized scaffolds should be used as a bactericide lead compound rather than an insecticide lead compound for further optimization and research.

## Data Availability

The original contributions presented in the study are included in the article/Supplementary Material; further inquiries can be directed to the corresponding author.
