# Supplementary material for: Synthesis and Bioactivities of Novel Piperonylic Acid Derivatives Containing a Sulfonic Acid Ester Moiety
Source: Front Chem. 2022 May 31;10:913003. doi: 10.3389/fchem.2022.913003 (PMC9192962; doi:10.3389/fchem.2022.913003)
Supplement: Supplementary file 1 [file DataSheet1.pdf]

Synthesis and Bioactivities of Novel Piperonylic Acid Derivatives Containing a Sulfonic Acid Esters Moiety

Dandan Xie,[1,*] Xin Hu,[2] Xiaoli Ren,[1] Zaiping Yang[3]

[1] *State Key Laboratory Breeding Base of Green Pesticide and Agricultural Bioengineering, Key Laboratory of Green Pesticide and Agricultural Bioengineering, Ministry of Education, Guizhou University, Huaxi District, Guiyang 550025, China*

[2] *School of Biological Sciences, Guizhou Education University, Wudang District, Guiyang 550018, China*

[3] *School of Biologi & Engineering, Guizhou Medical University, Huaxi District, Guiyang 550025, China*

* Author to whom correspondence should be addressed; E-mail: xddxed@163.com

Spectral data for compounds 4a-4x

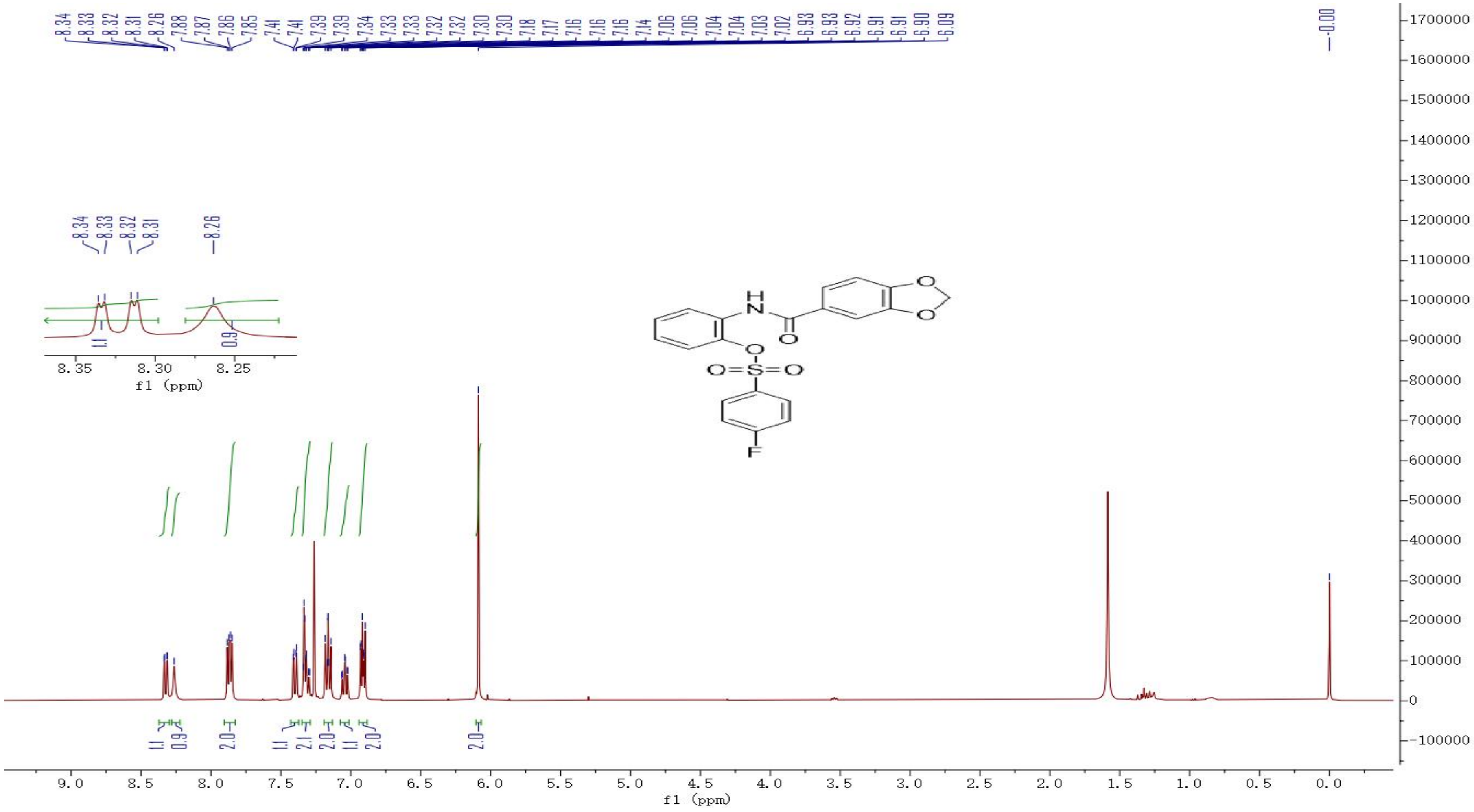

^{1}H NMR of Compound **4a**

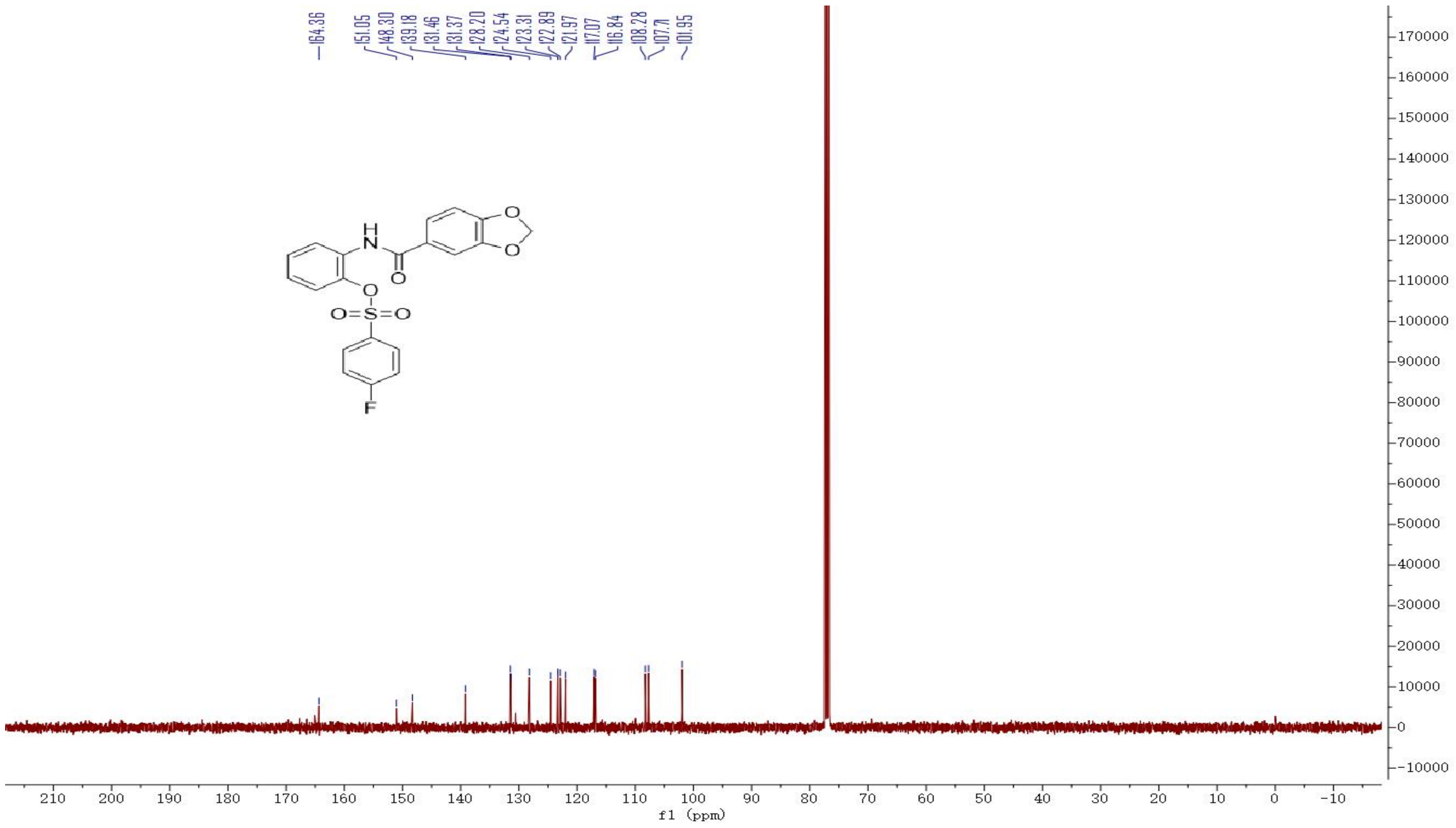

^{13}C NMR of Compound **4a**

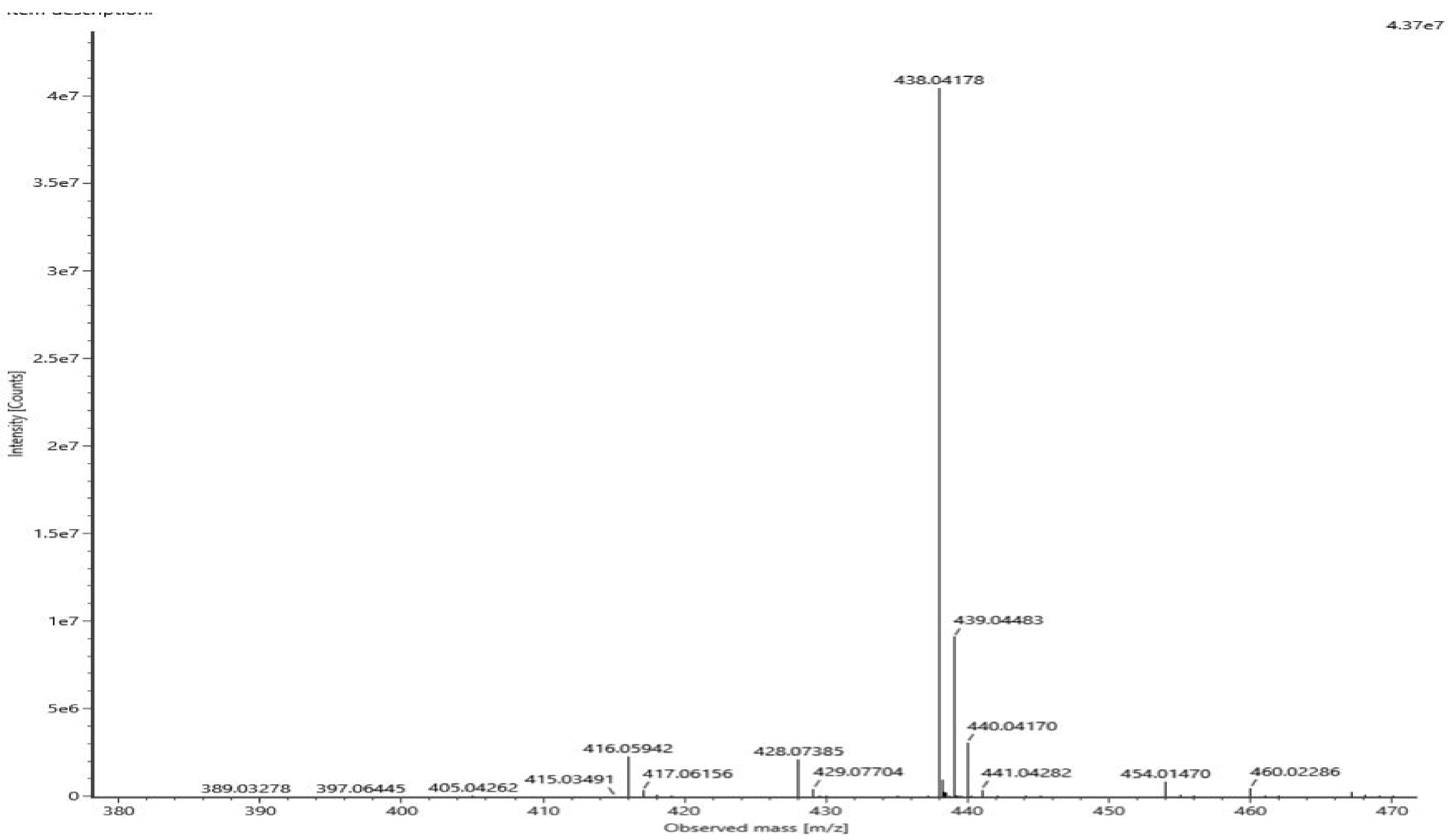

HRMS of Compound **4a**

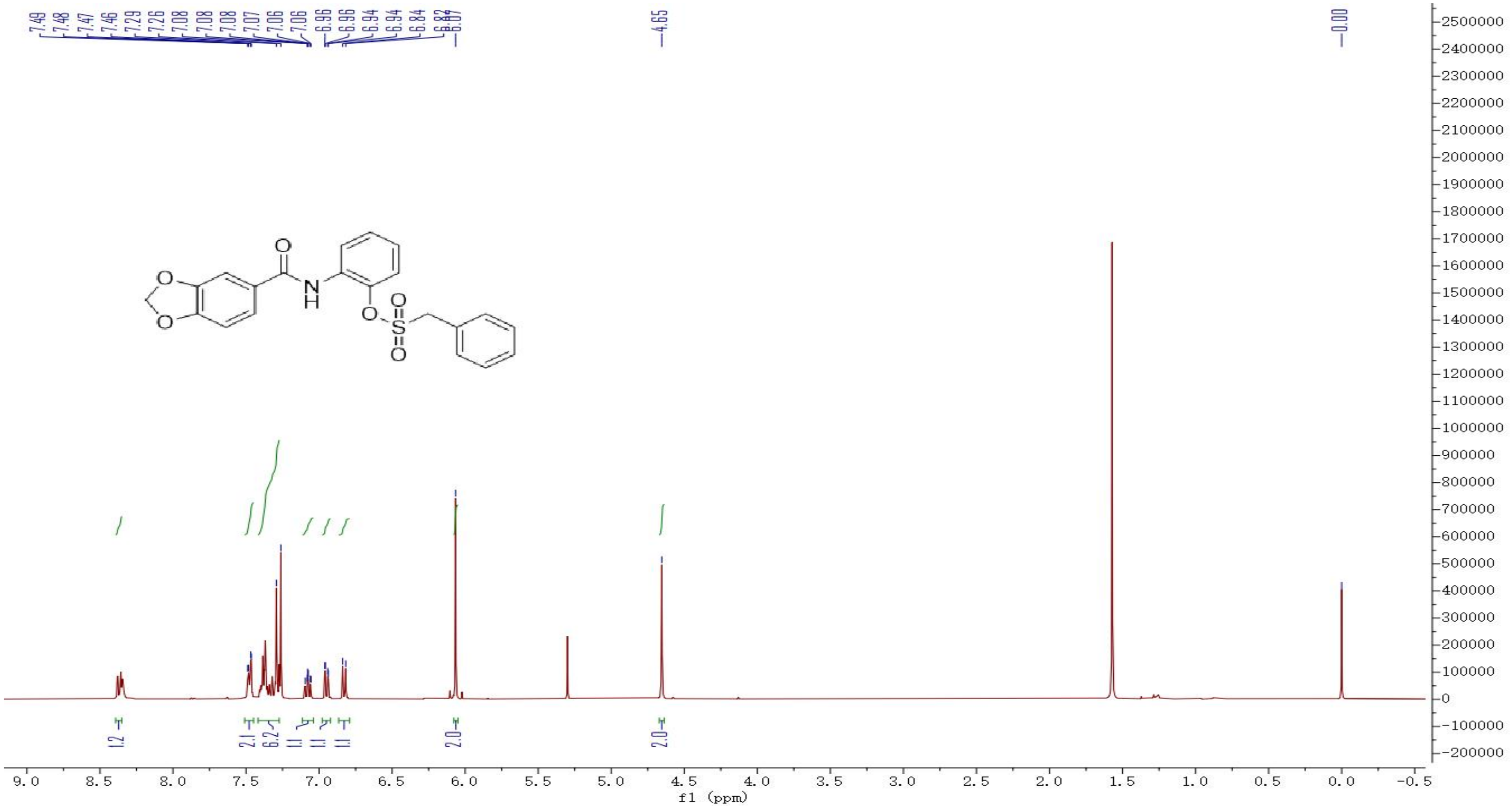

^{1}H NMR of Compound **4b**

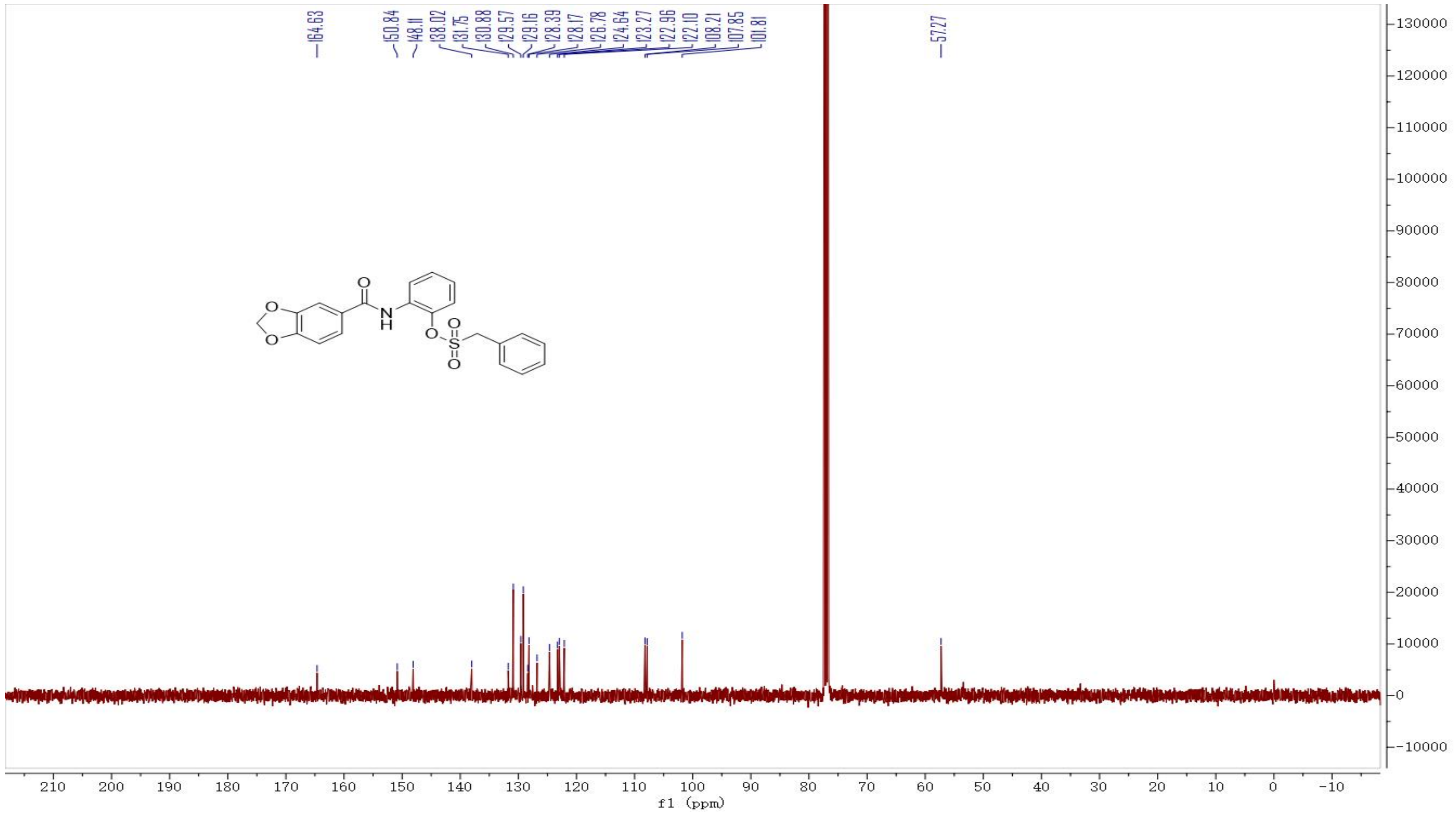

^{13}C NMR of Compound **4b**

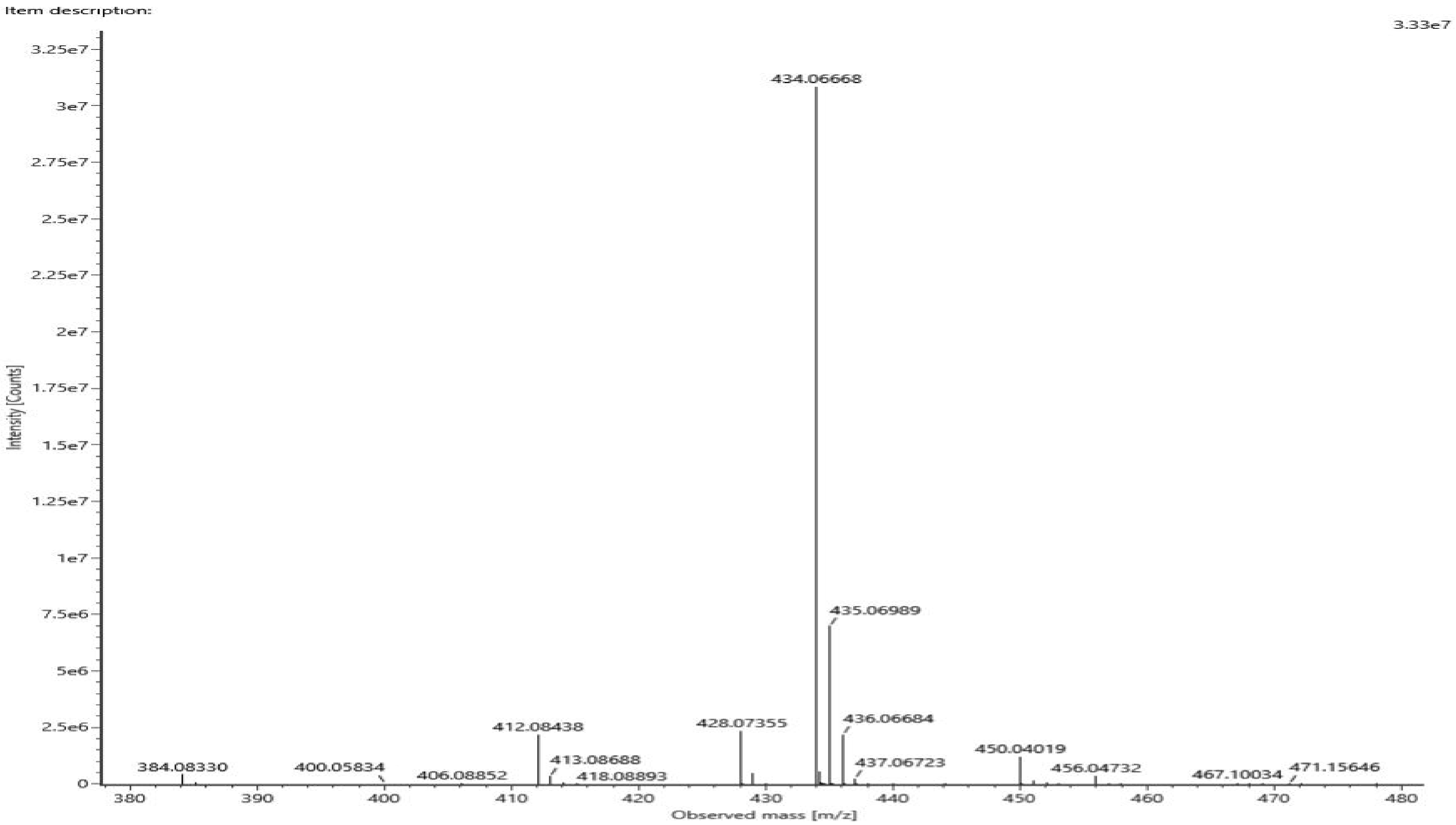

HRMS of Compound **4b**

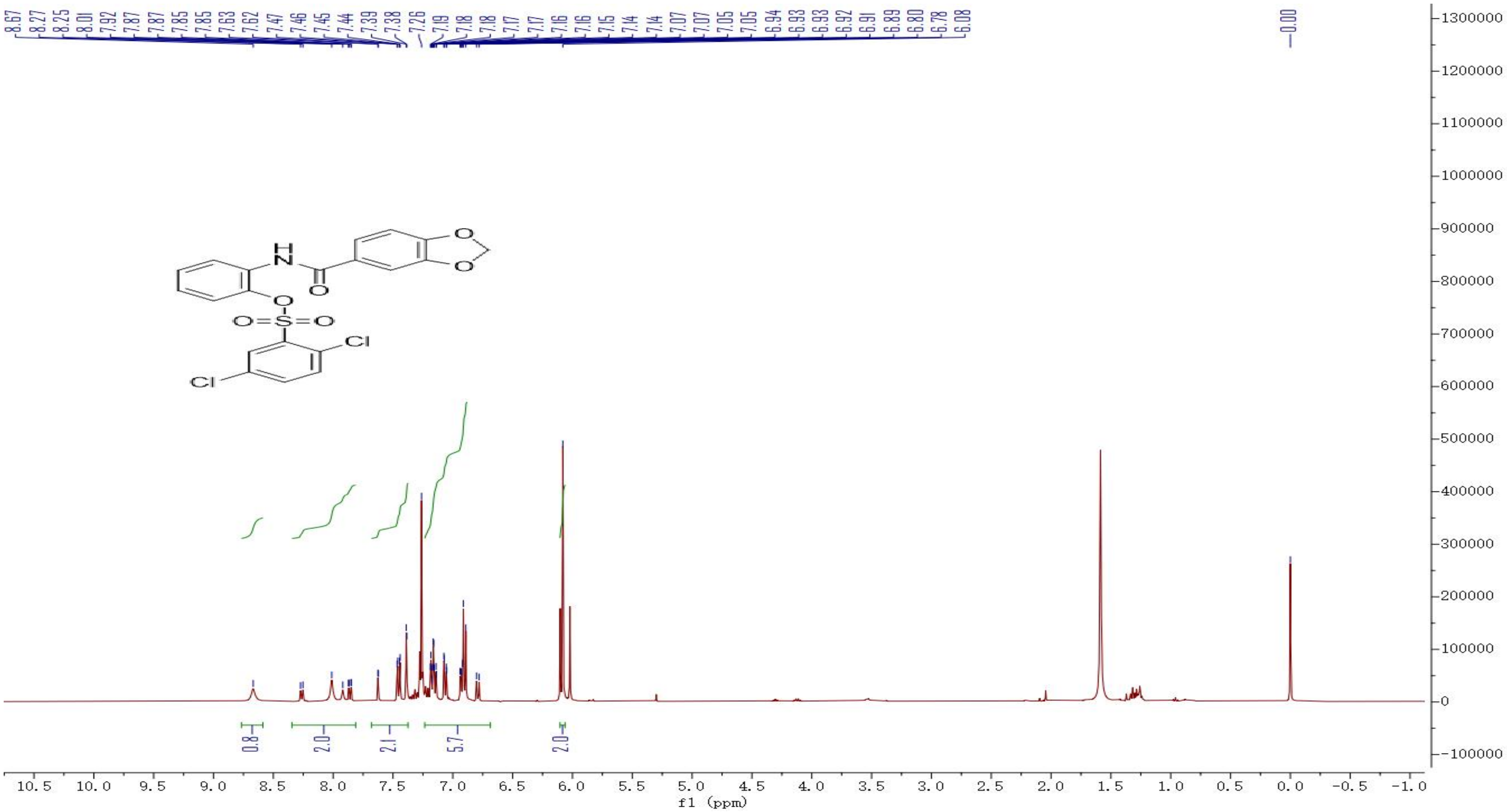

^{1}H NMR of Compound **4c**

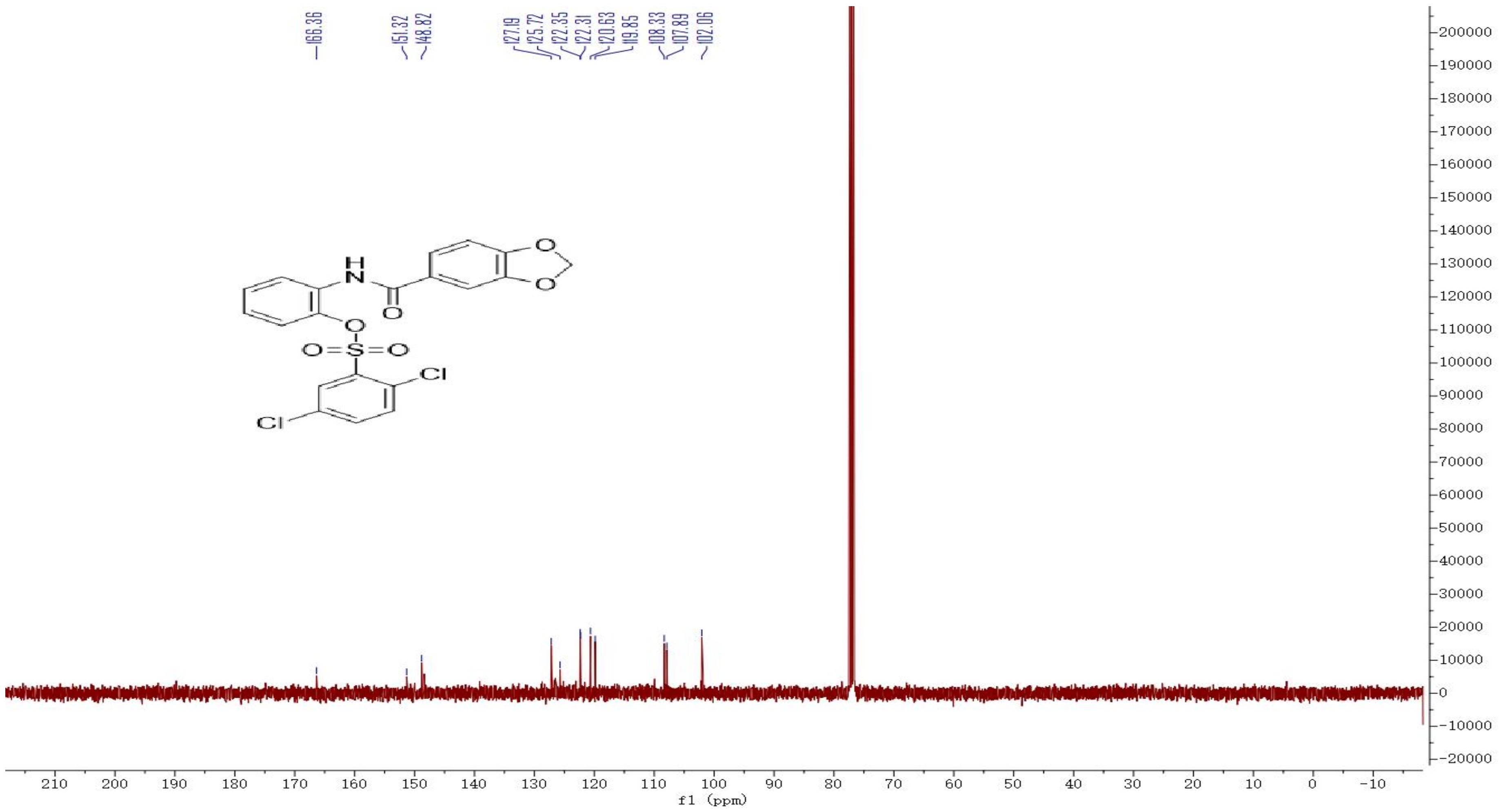

^{13}C NMR of Compound **4c**

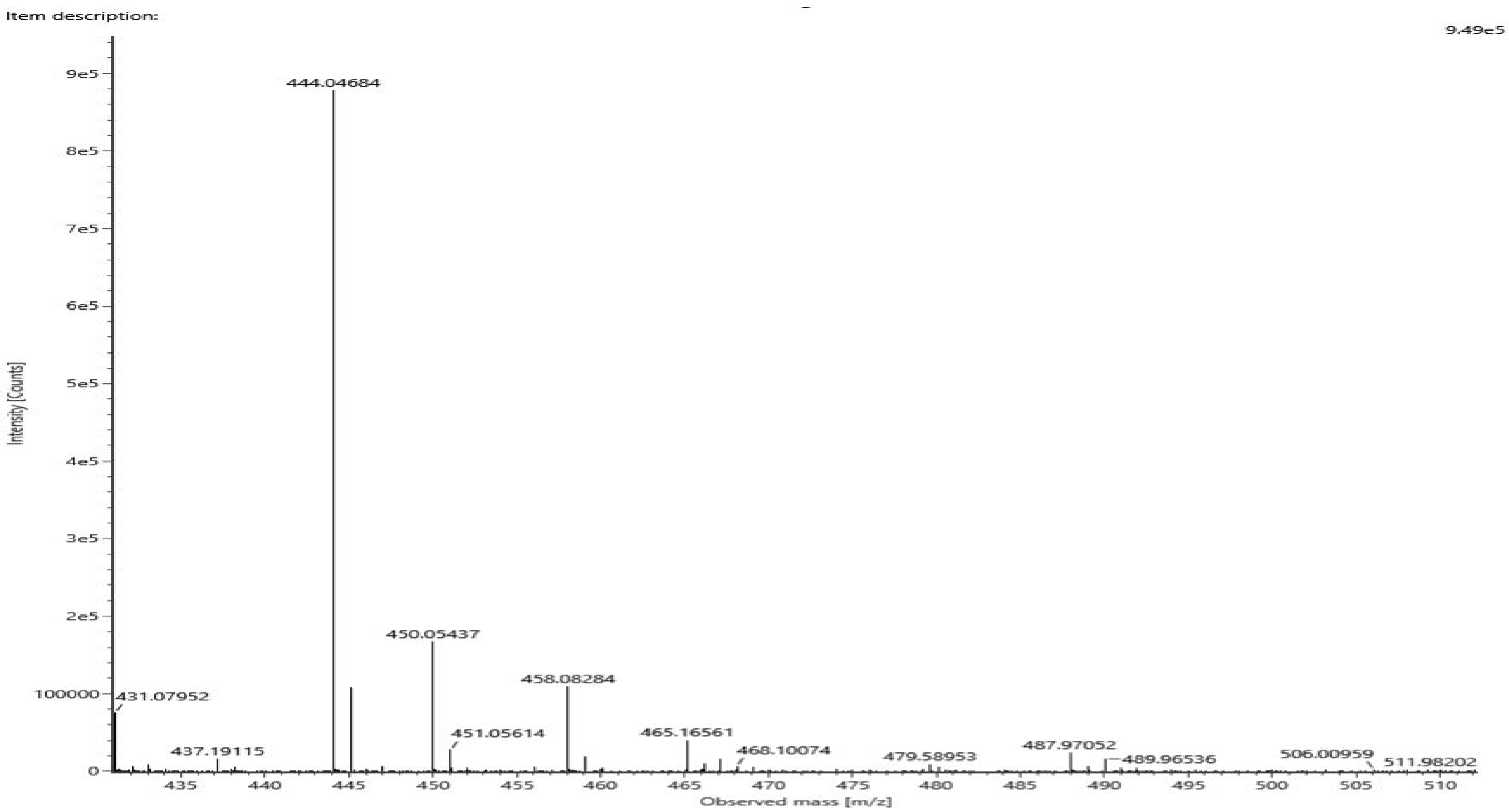

HRMS of Compound **4c**

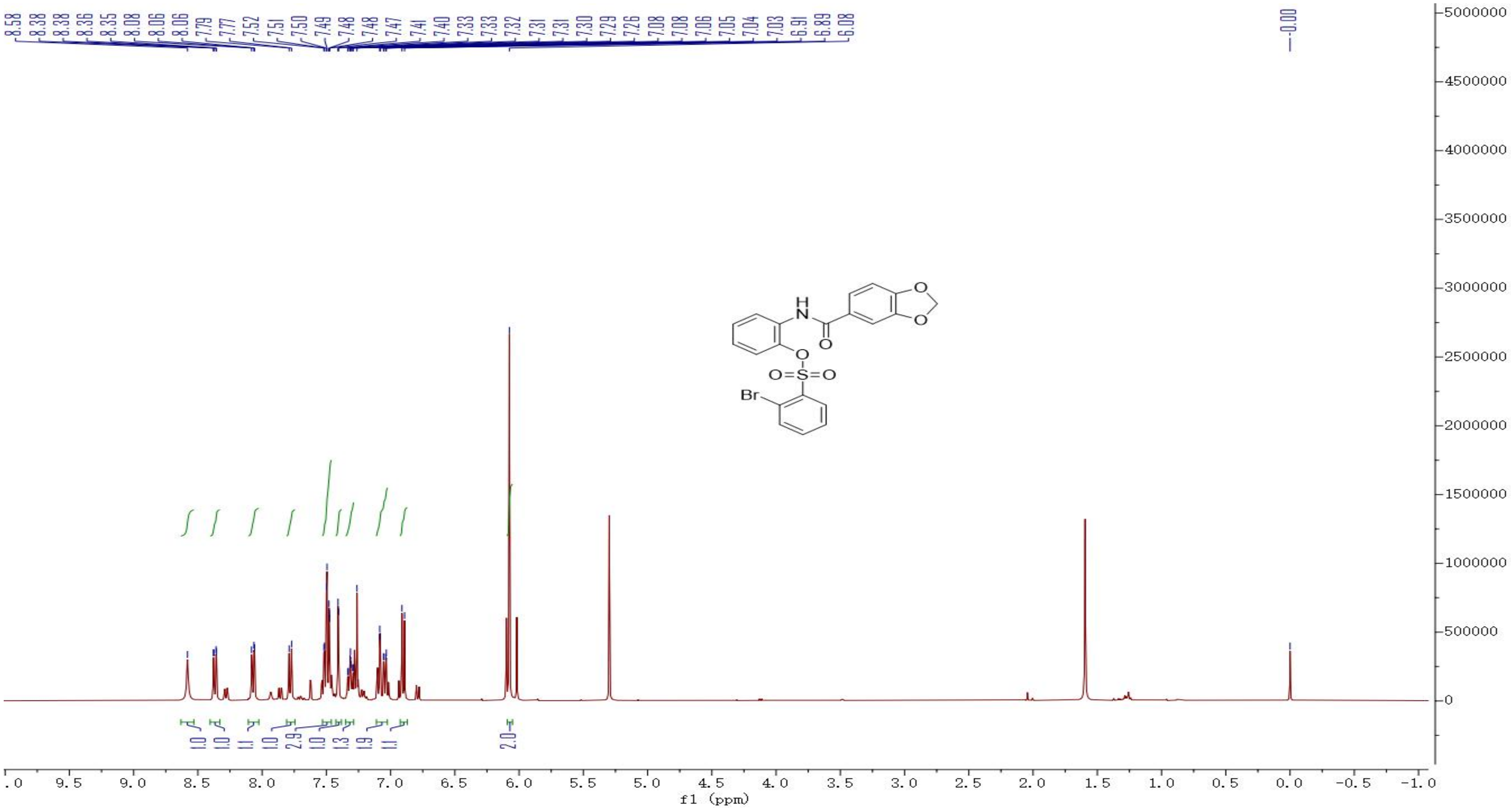

^{1}H NMR of Compound **4d**

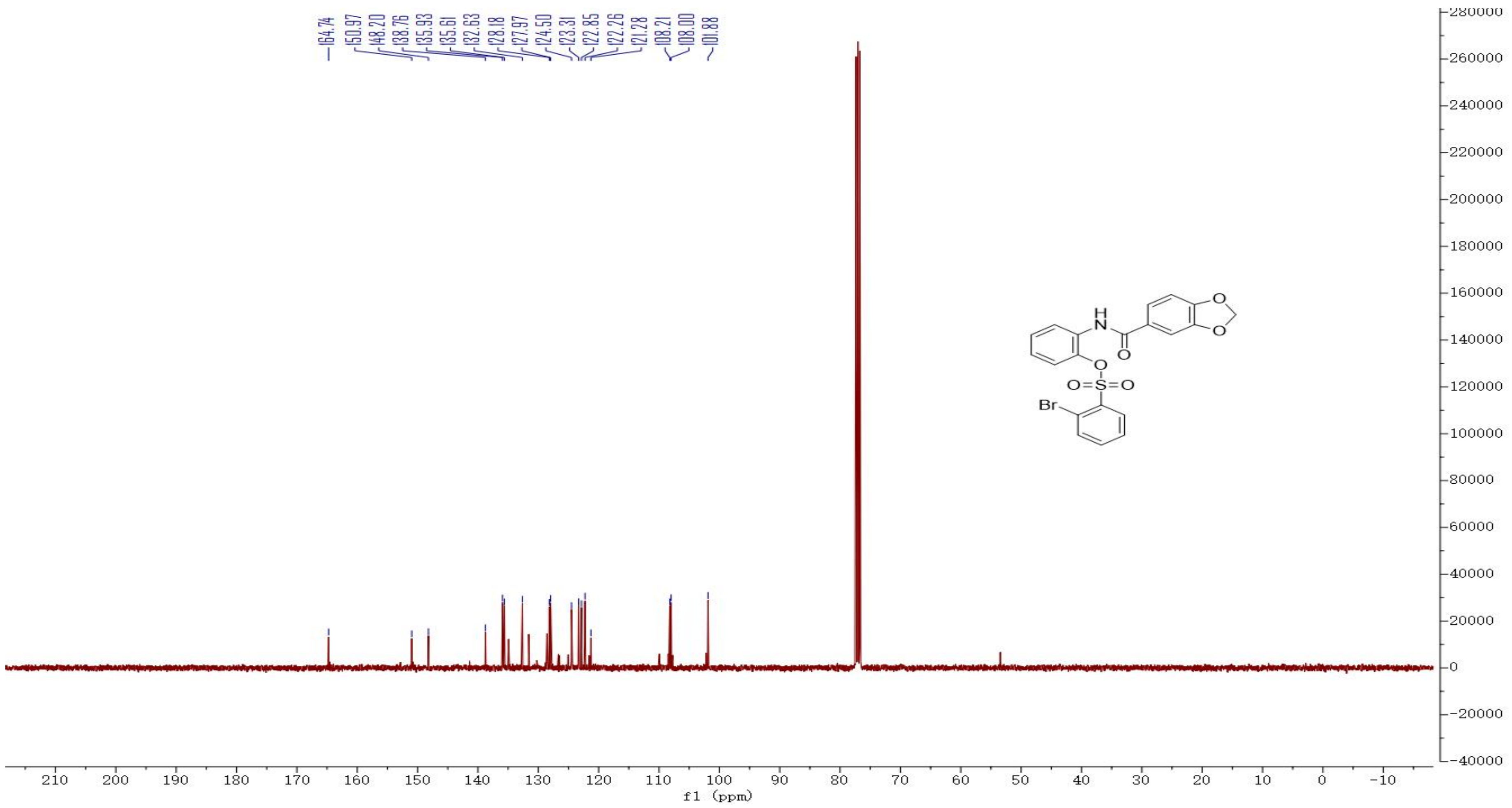

^{13}C NMR of Compound **4d**

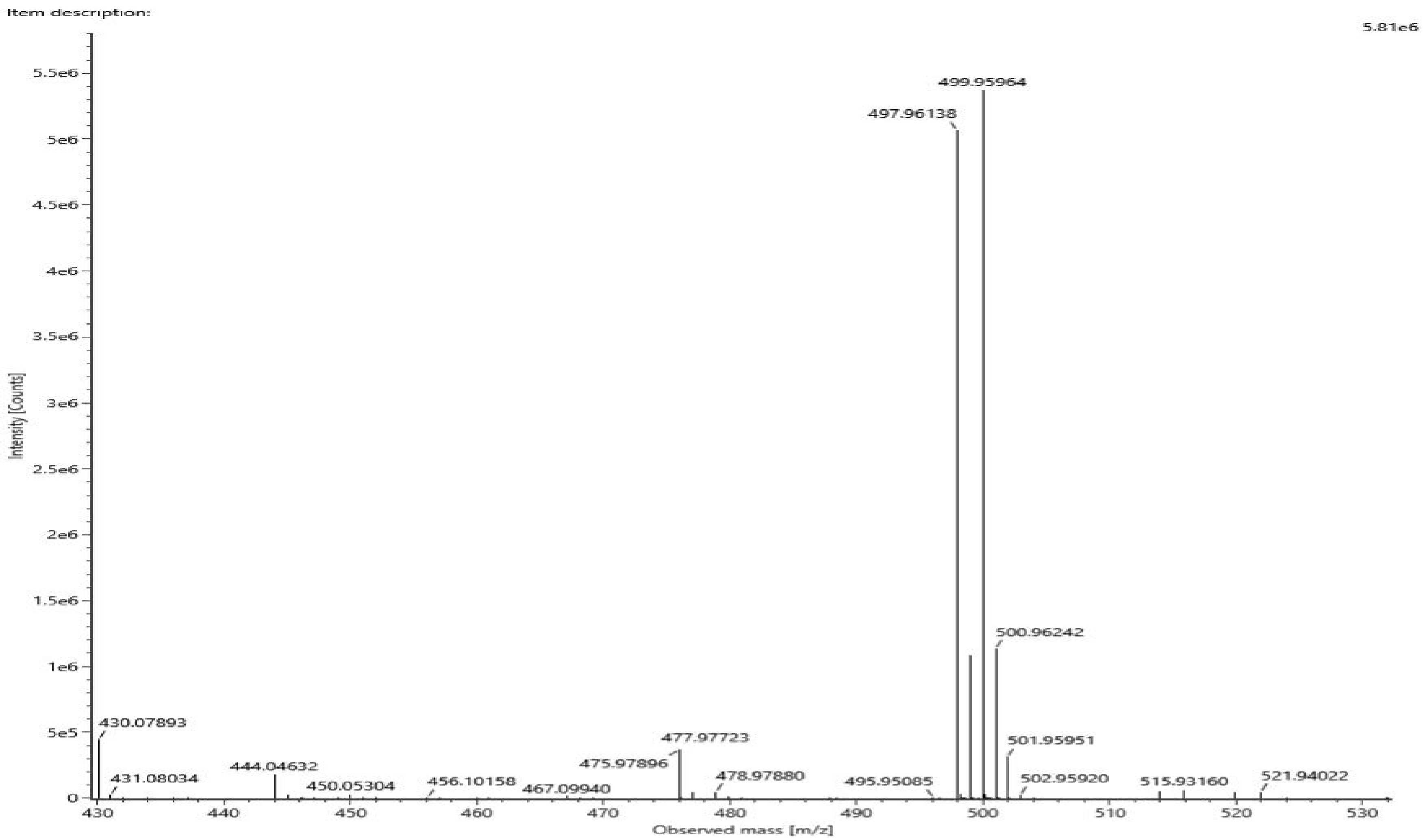

HRMS of Compound **4d**

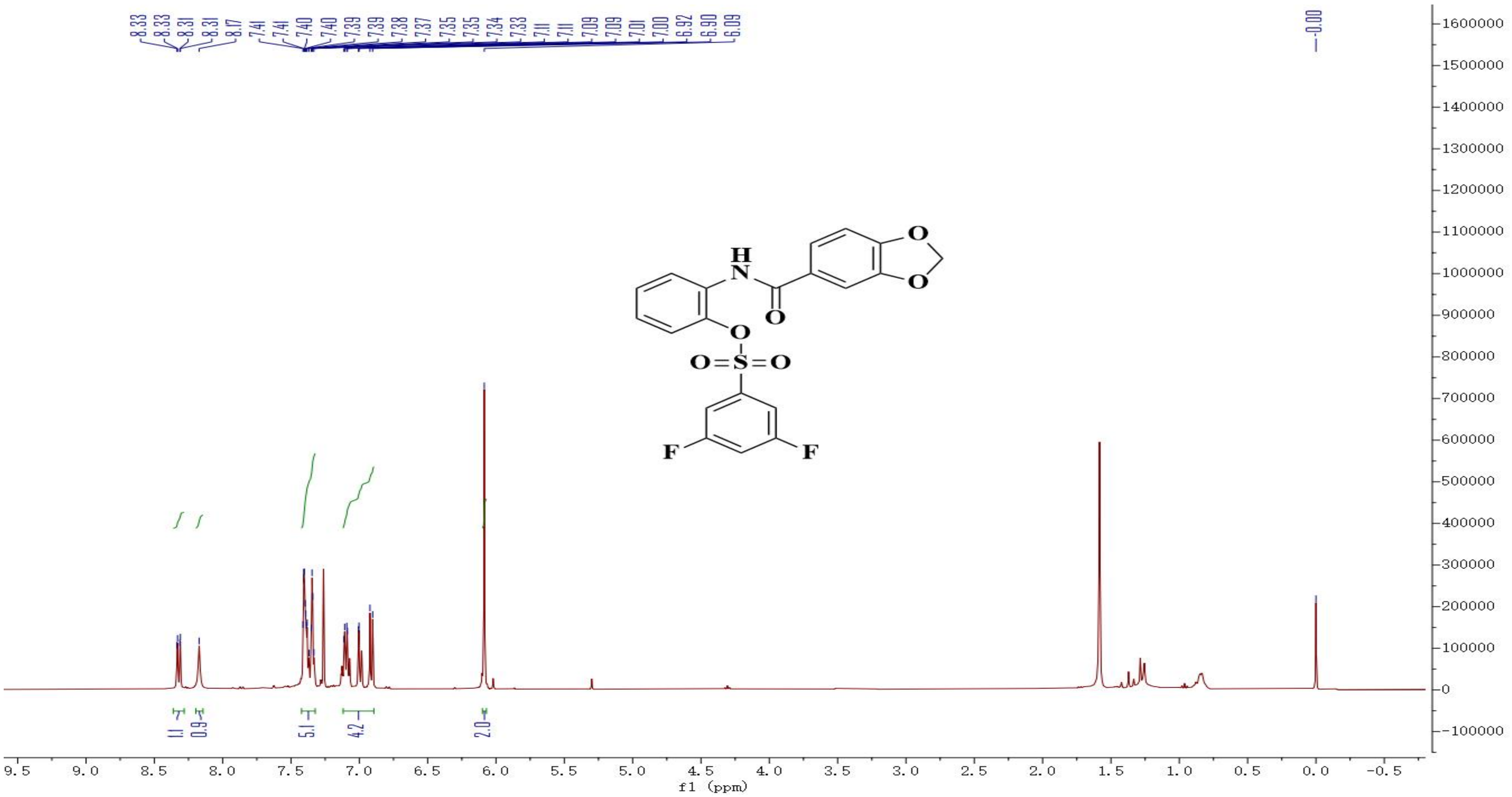

^{1}H NMR of Compound **4e**

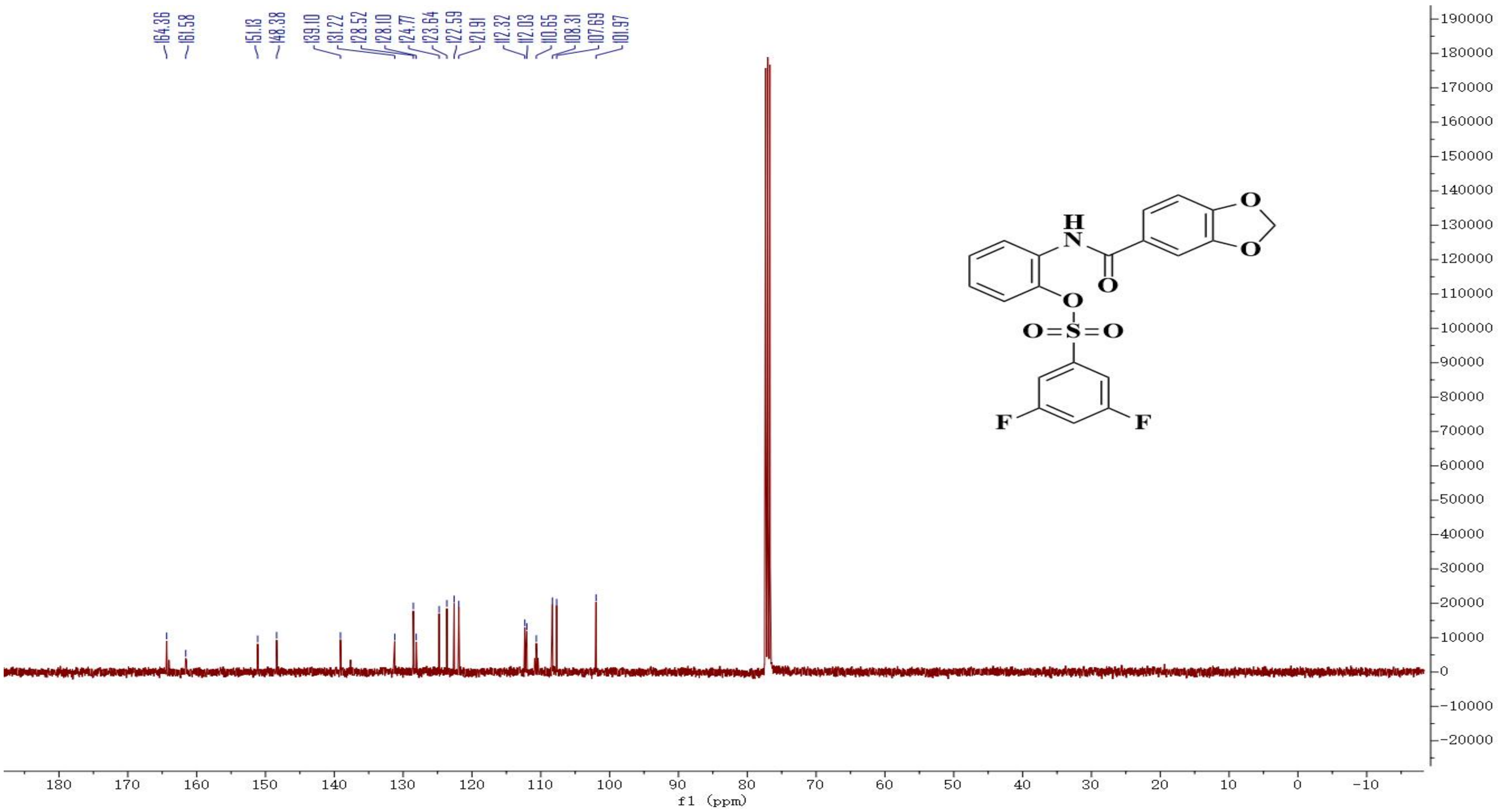

^{13}C NMR of Compound **4e**

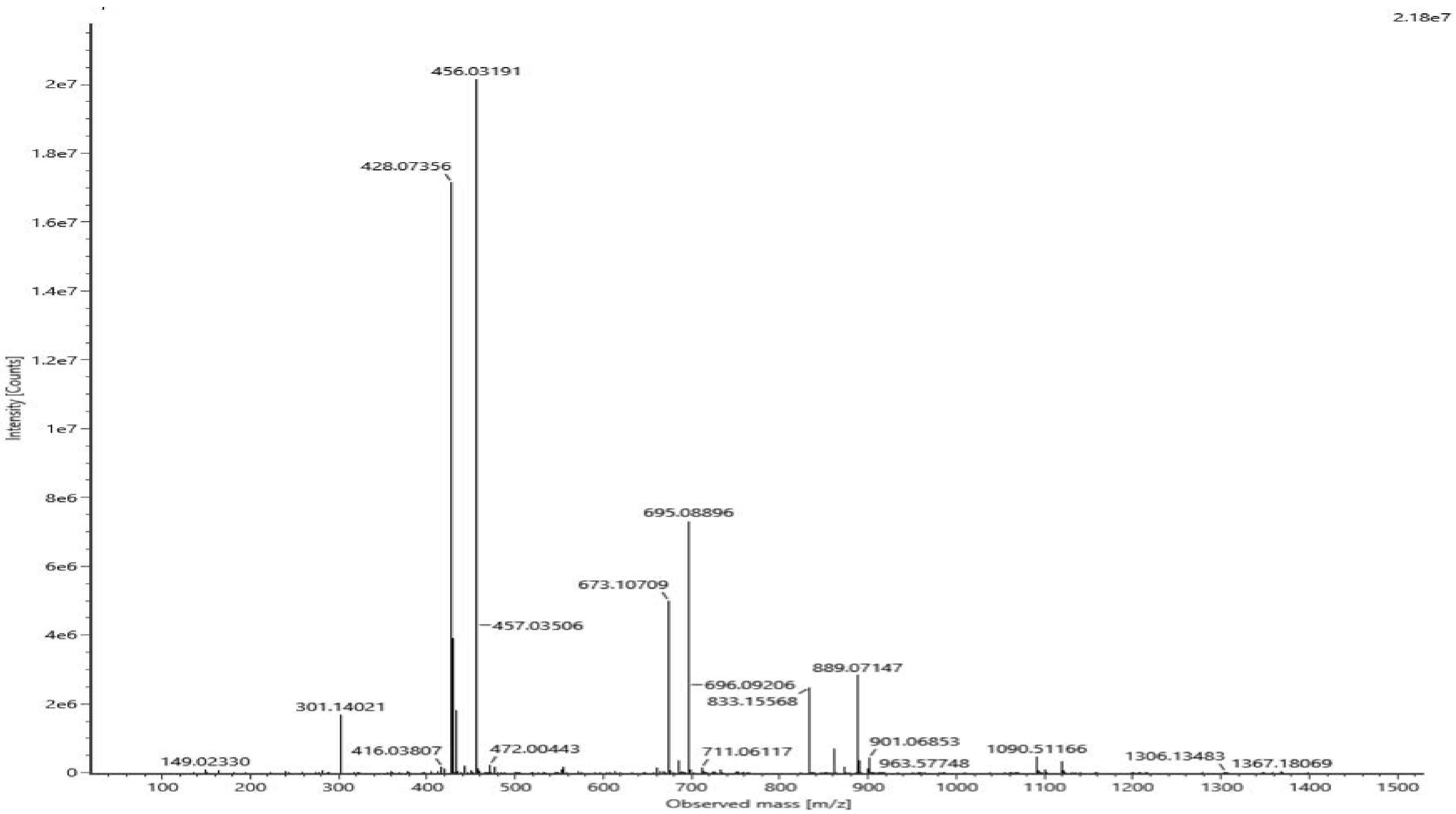

2.18e7
456.03191
428.07356
457.03506
695.08896
673.10709
696.09206
833.15568
889.07147
301.14021
416.03807
472.00443
711.06117
901.06853
963.57748
1090.51166
1306.13483
1367.18069
149.02330
Intensity [Counts]
Observed mass [m/z]

HRMS of Compound **4e**

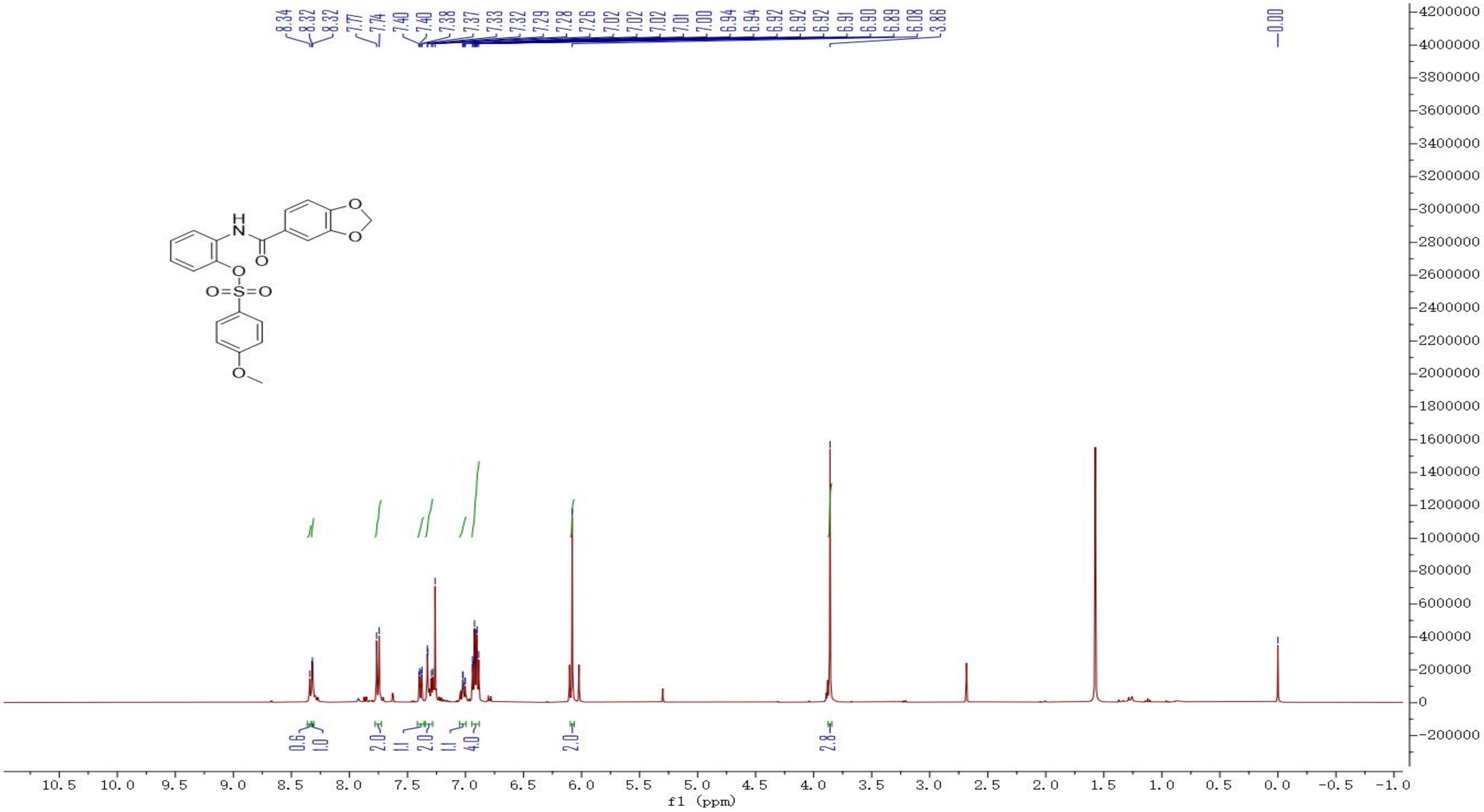

^{1}H NMR of Compound **4f**

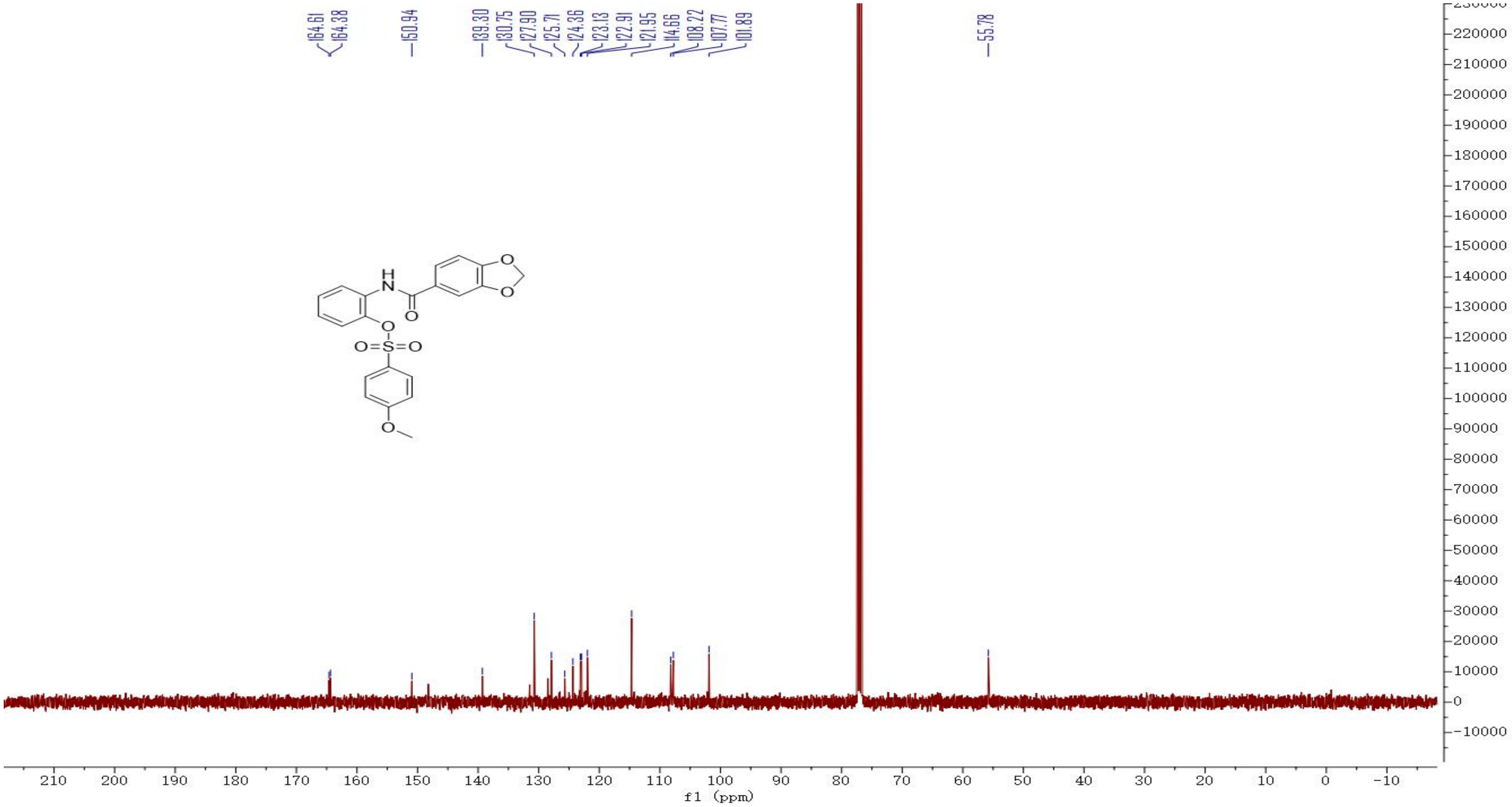

^{13}C NMR of Compound **4f**

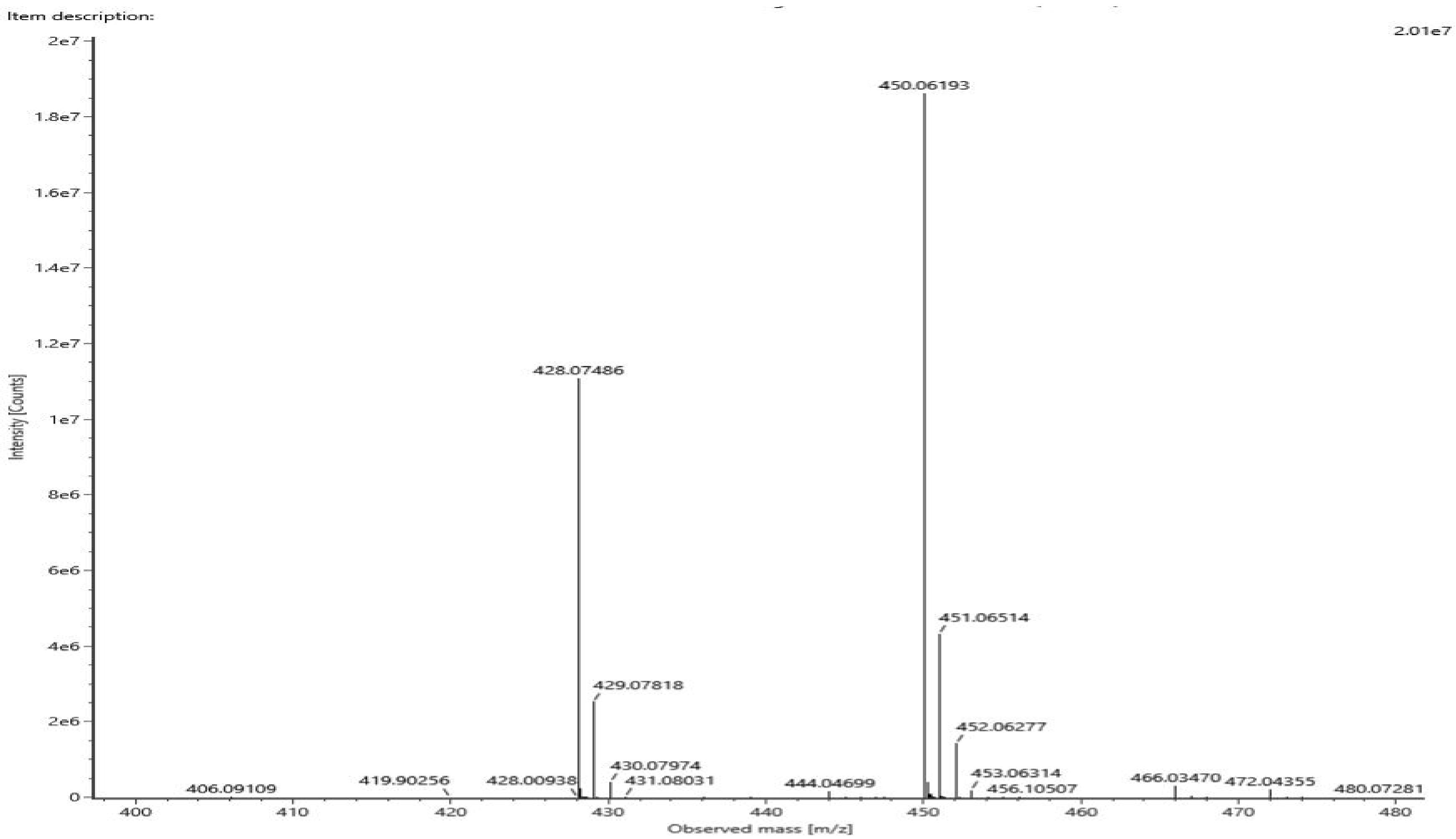

HRMS of Compound **4f**

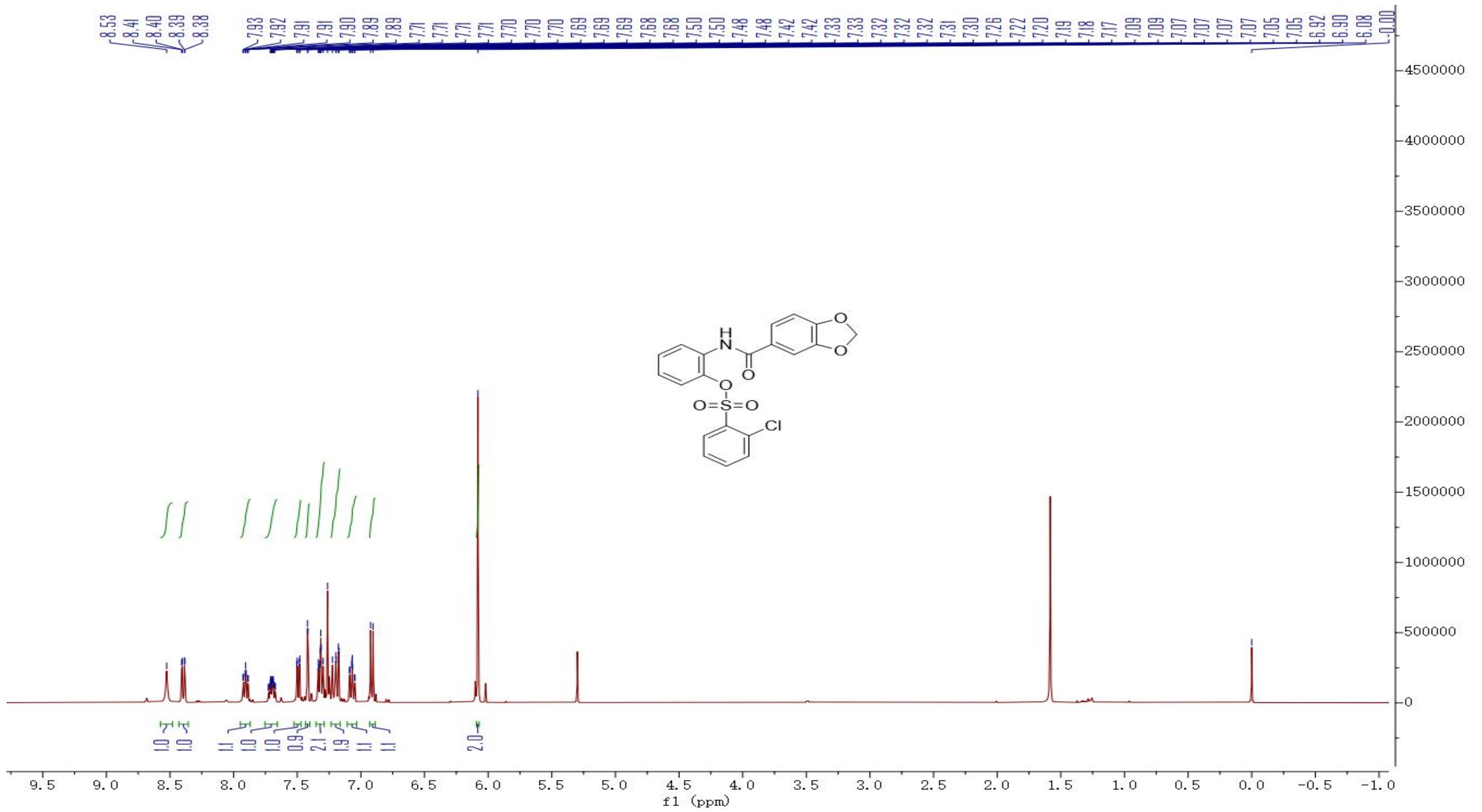

^{1}H NMR of Compound **4g**

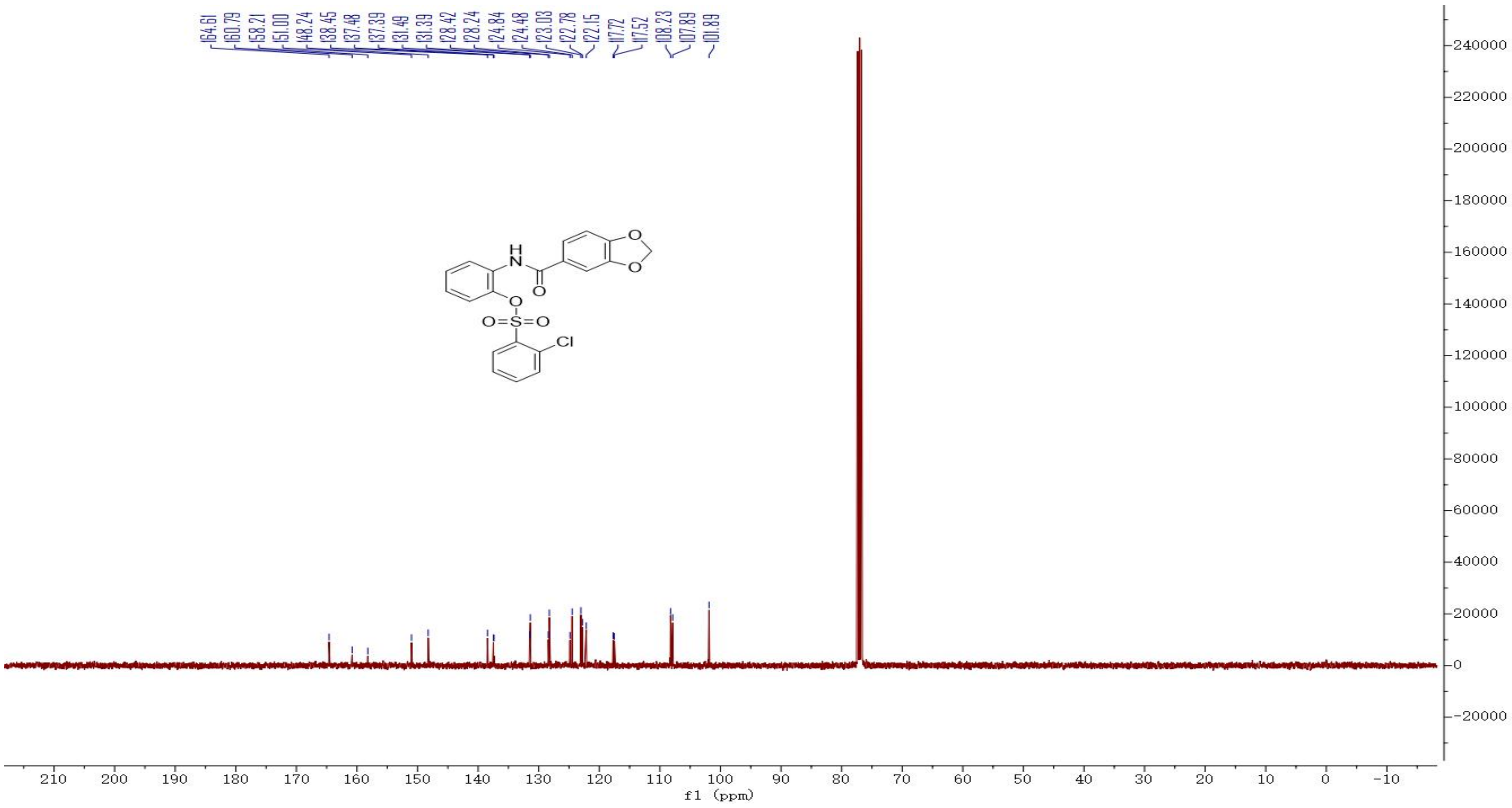

^{13}C NMR of Compound **4g**

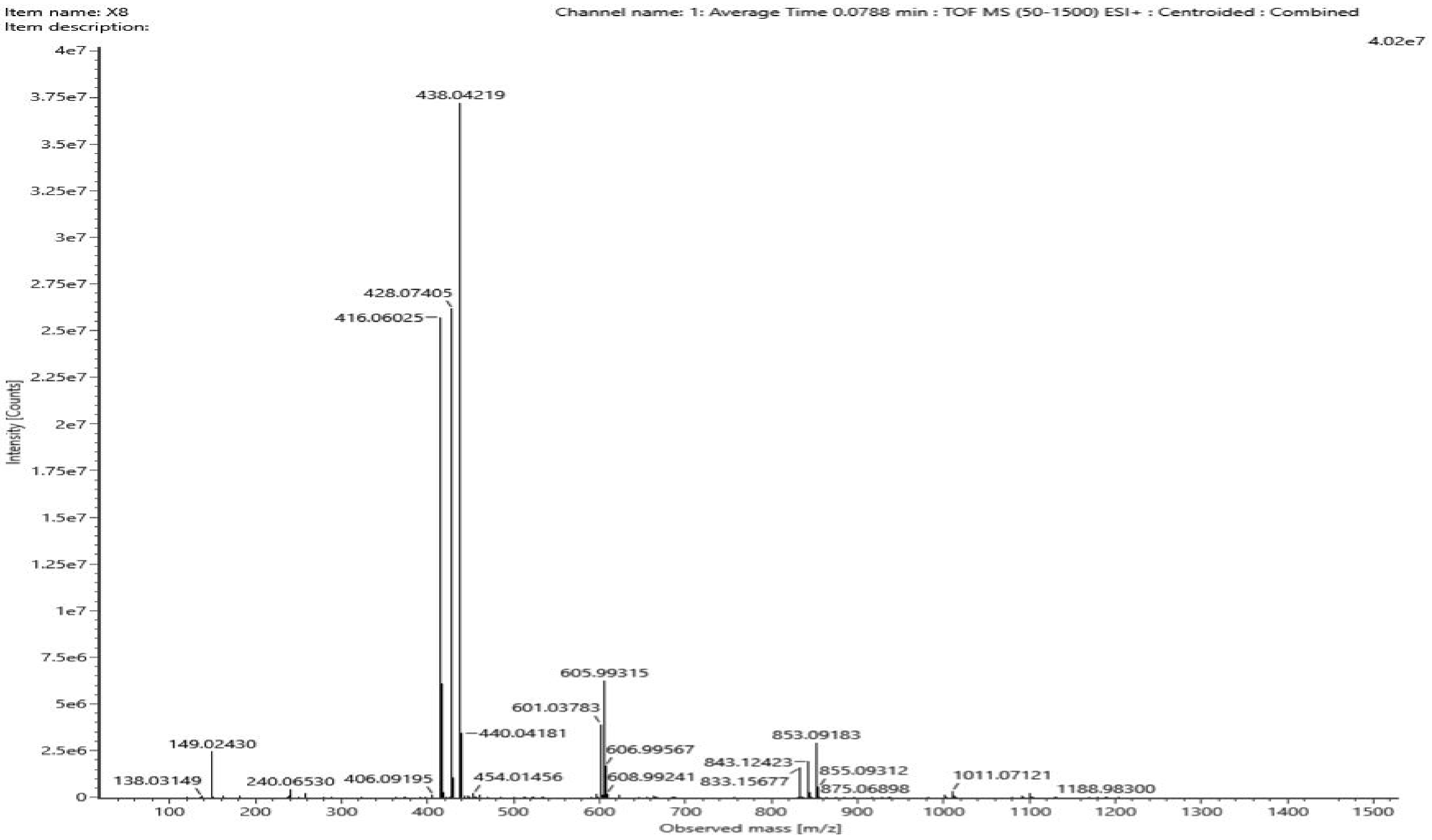

HRMS of Compound **4g**

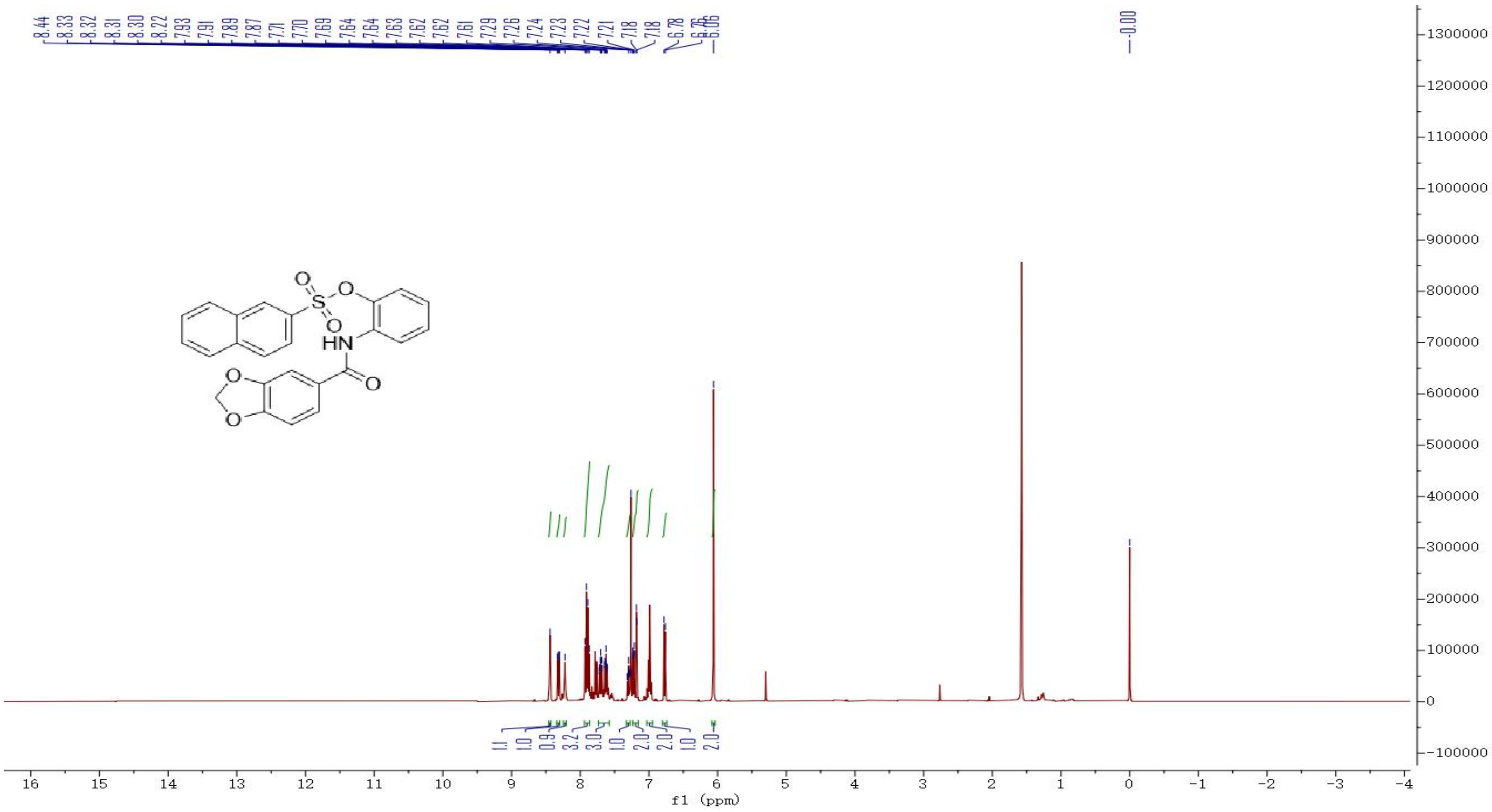

^{1}H NMR of Compound **4h**

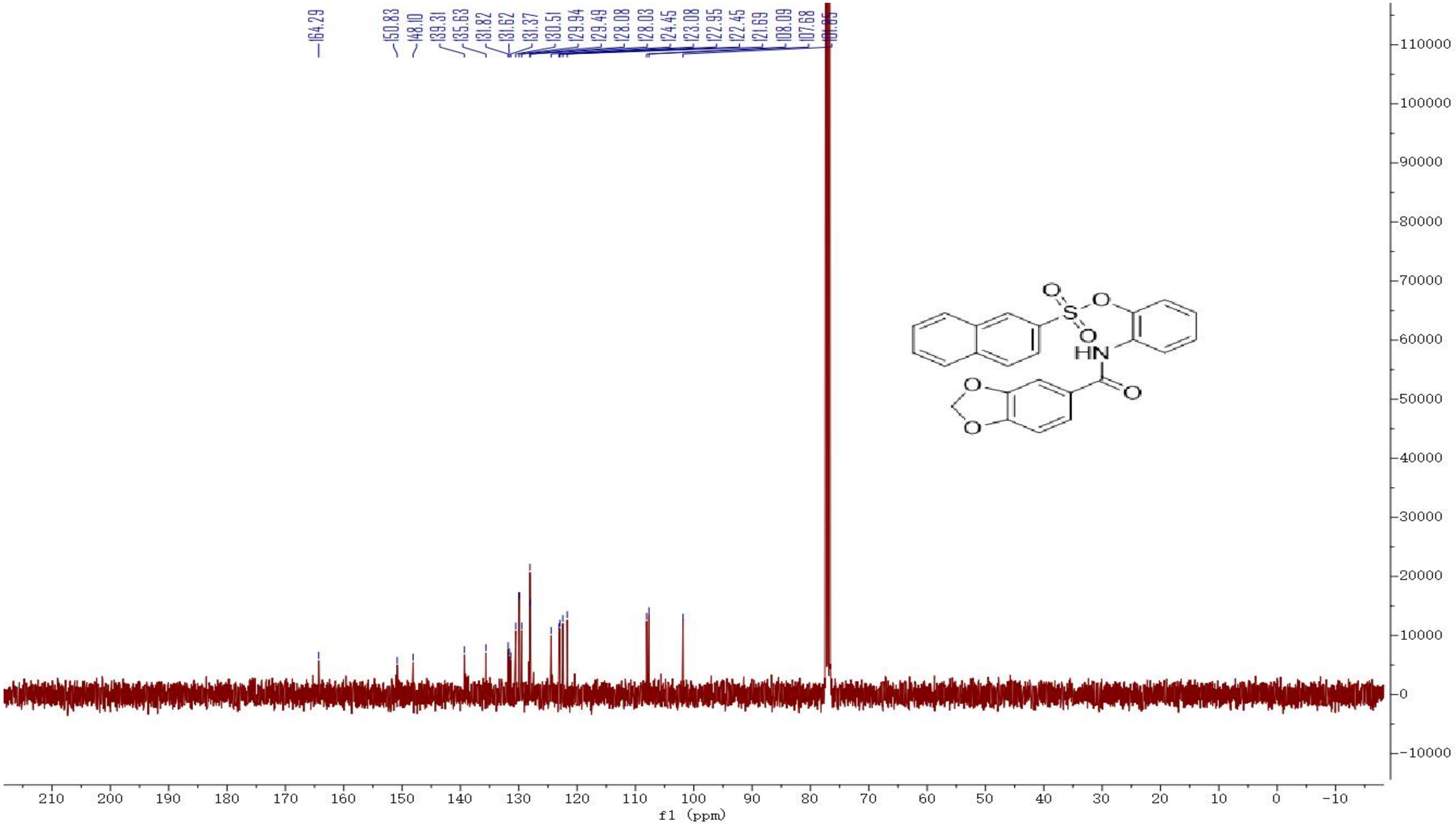

^{13}C NMR of Compound **4h**

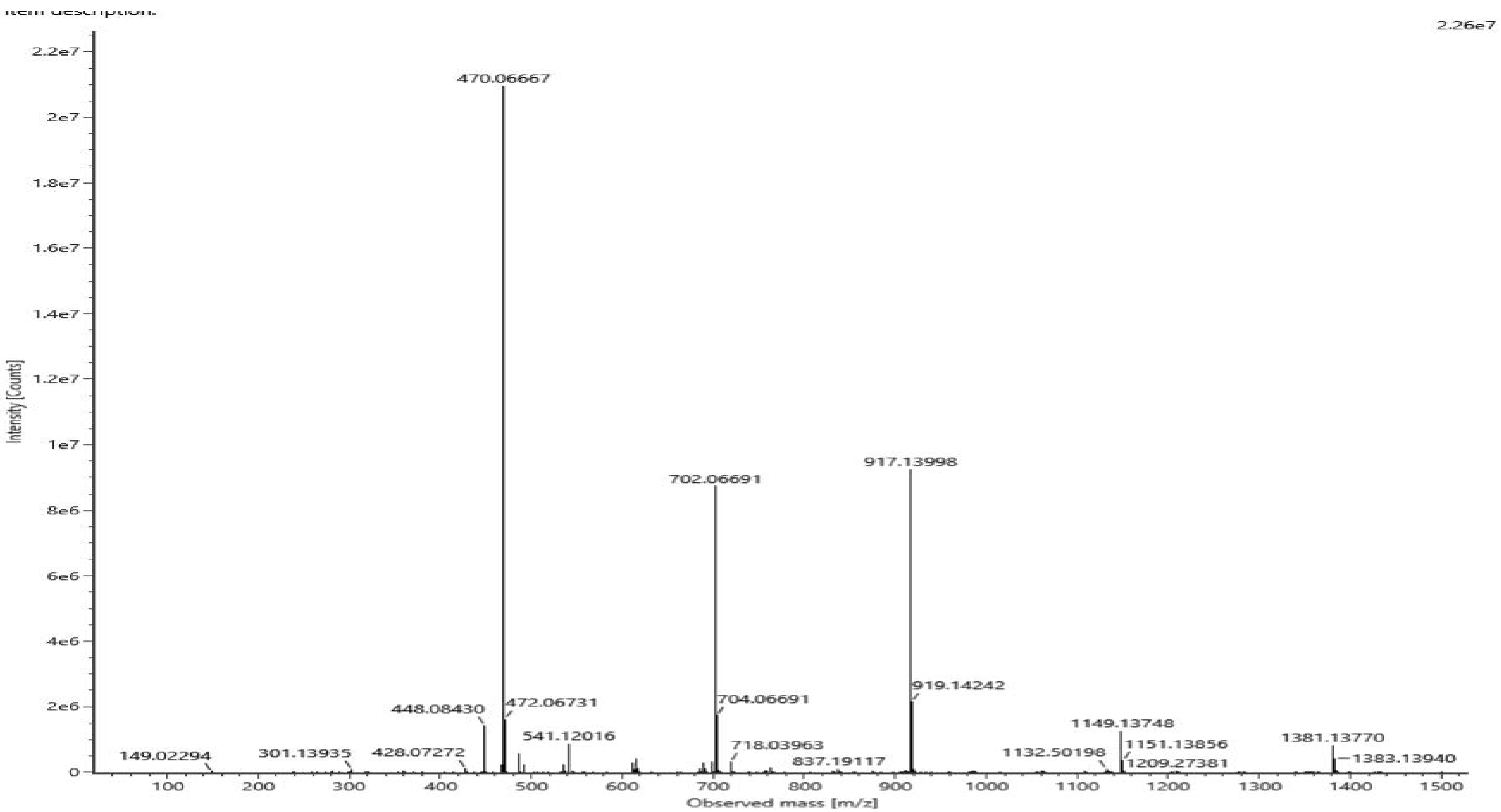

HRMS of Compound **4h**

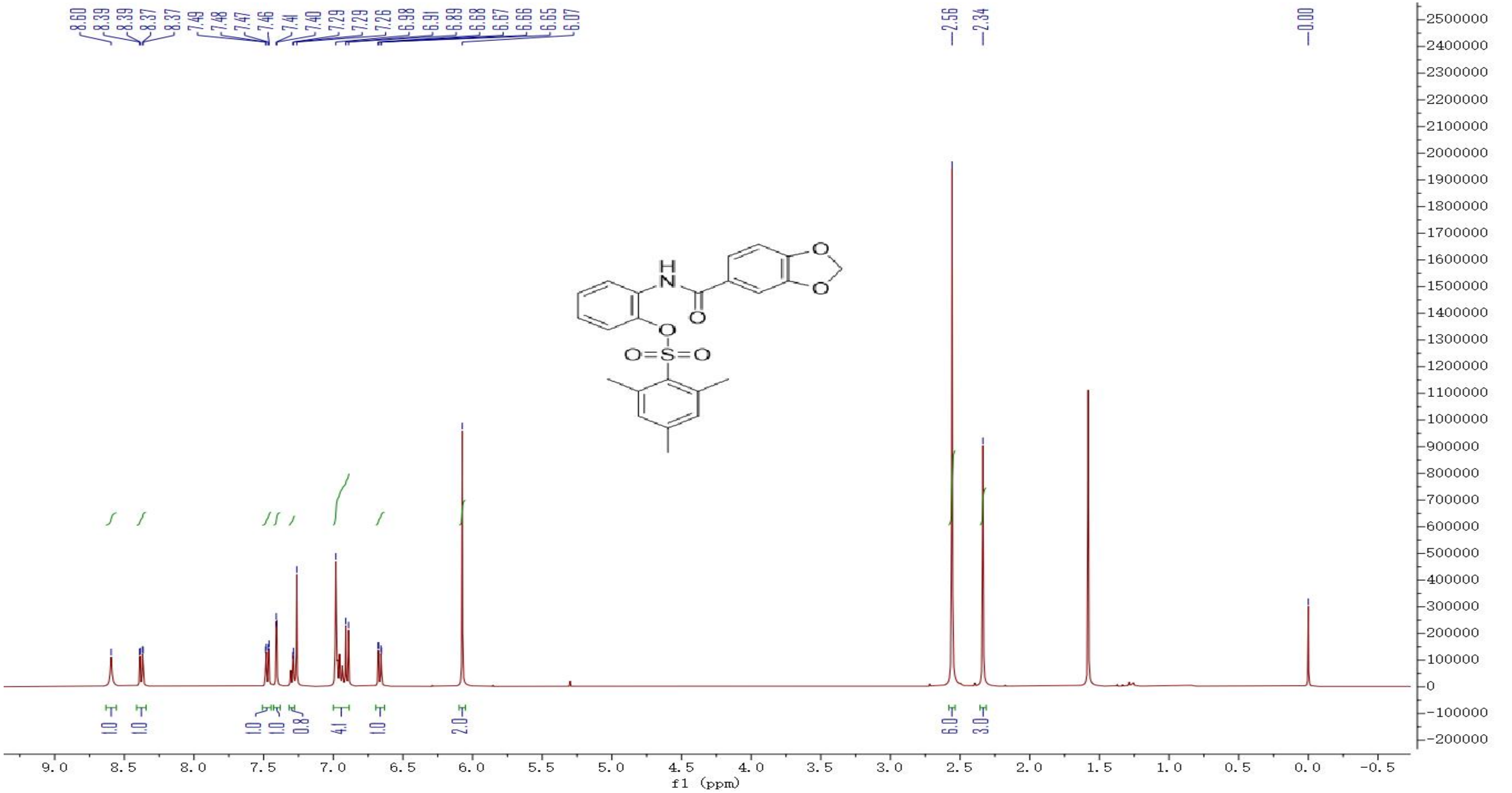

^{1}H NMR of Compound **4i**

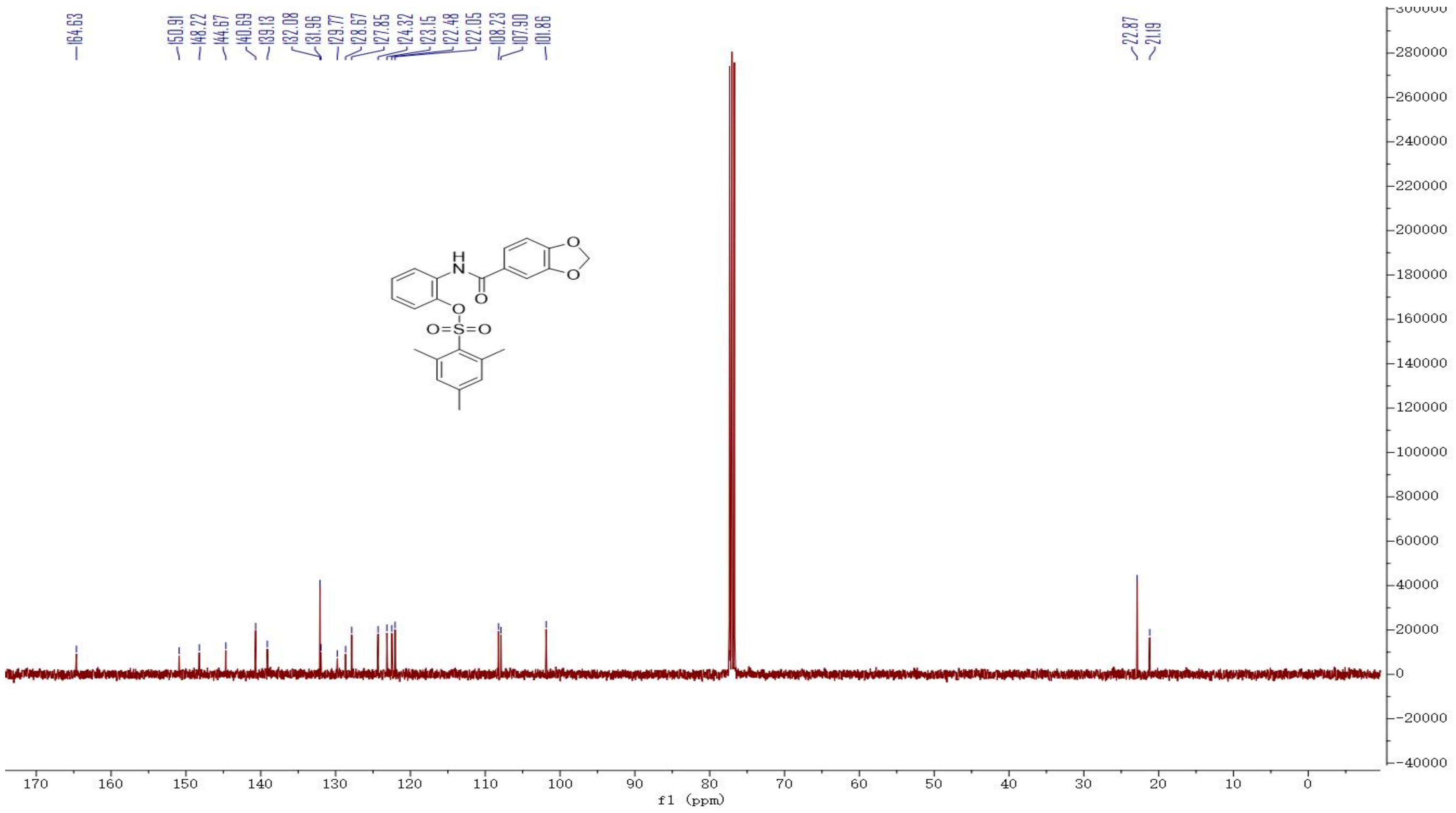

^{13}C NMR of Compound **4i**

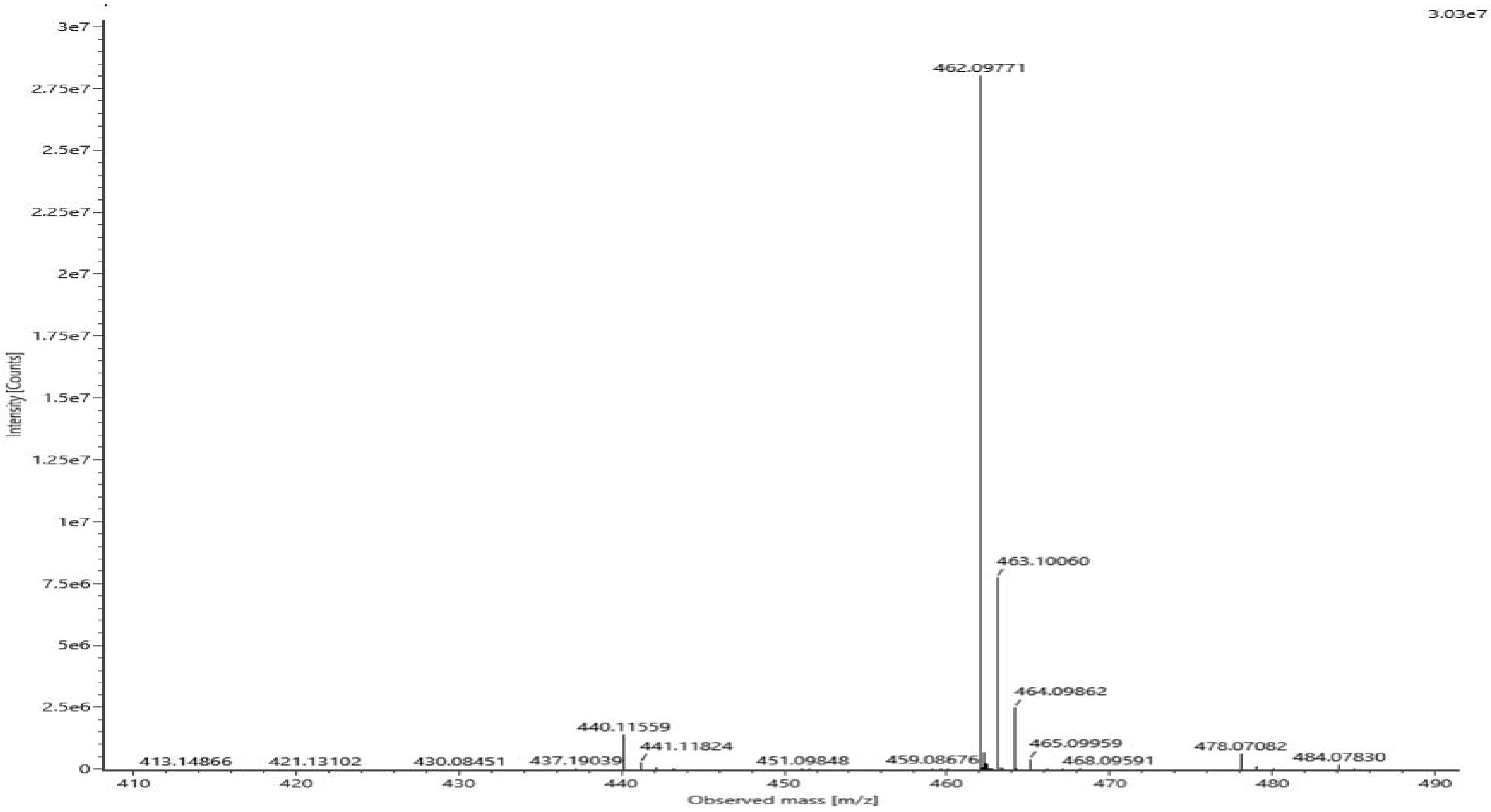

HRMS of Compound **4i**

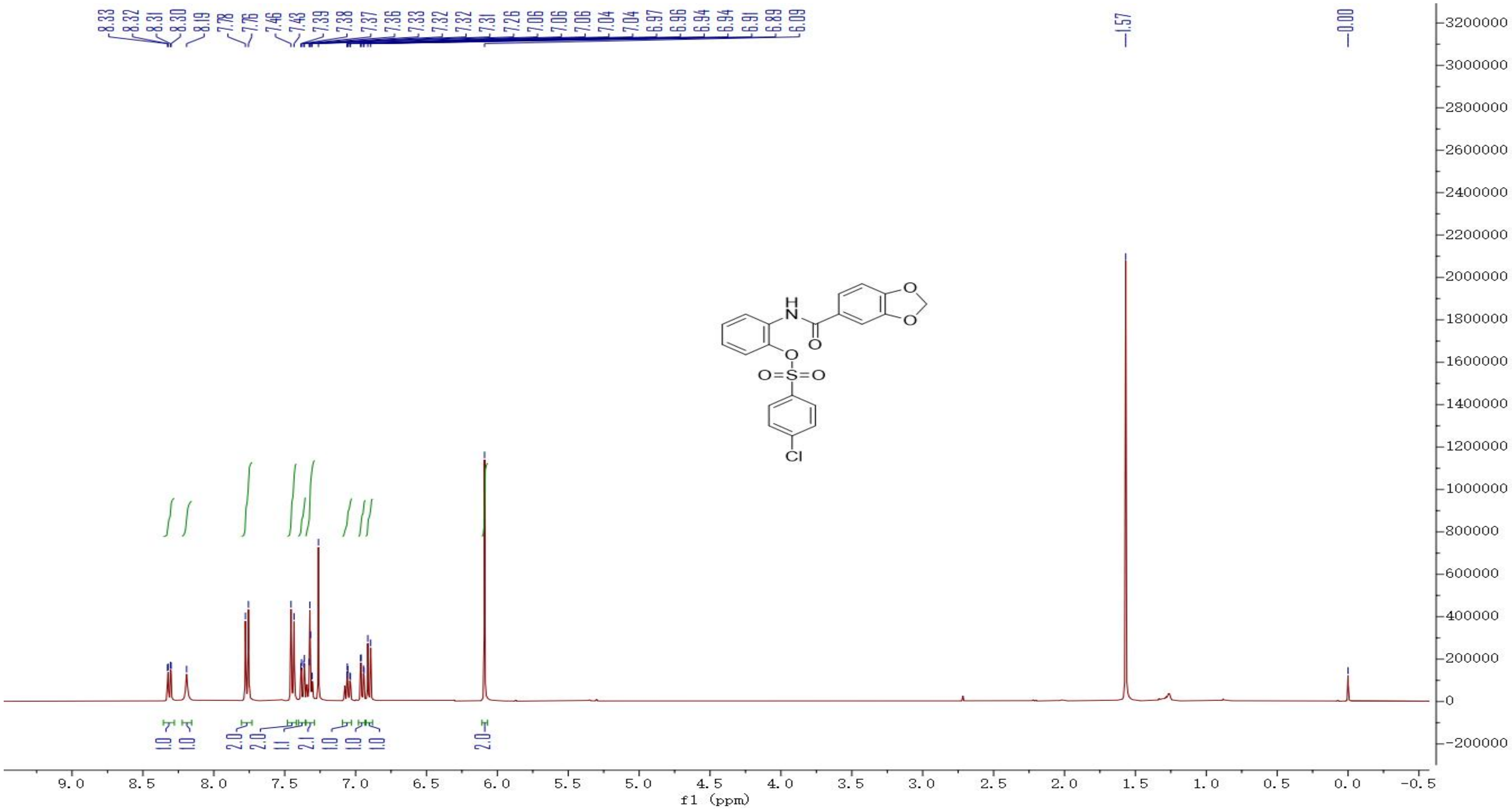

^{1}H NMR of Compound **4j**

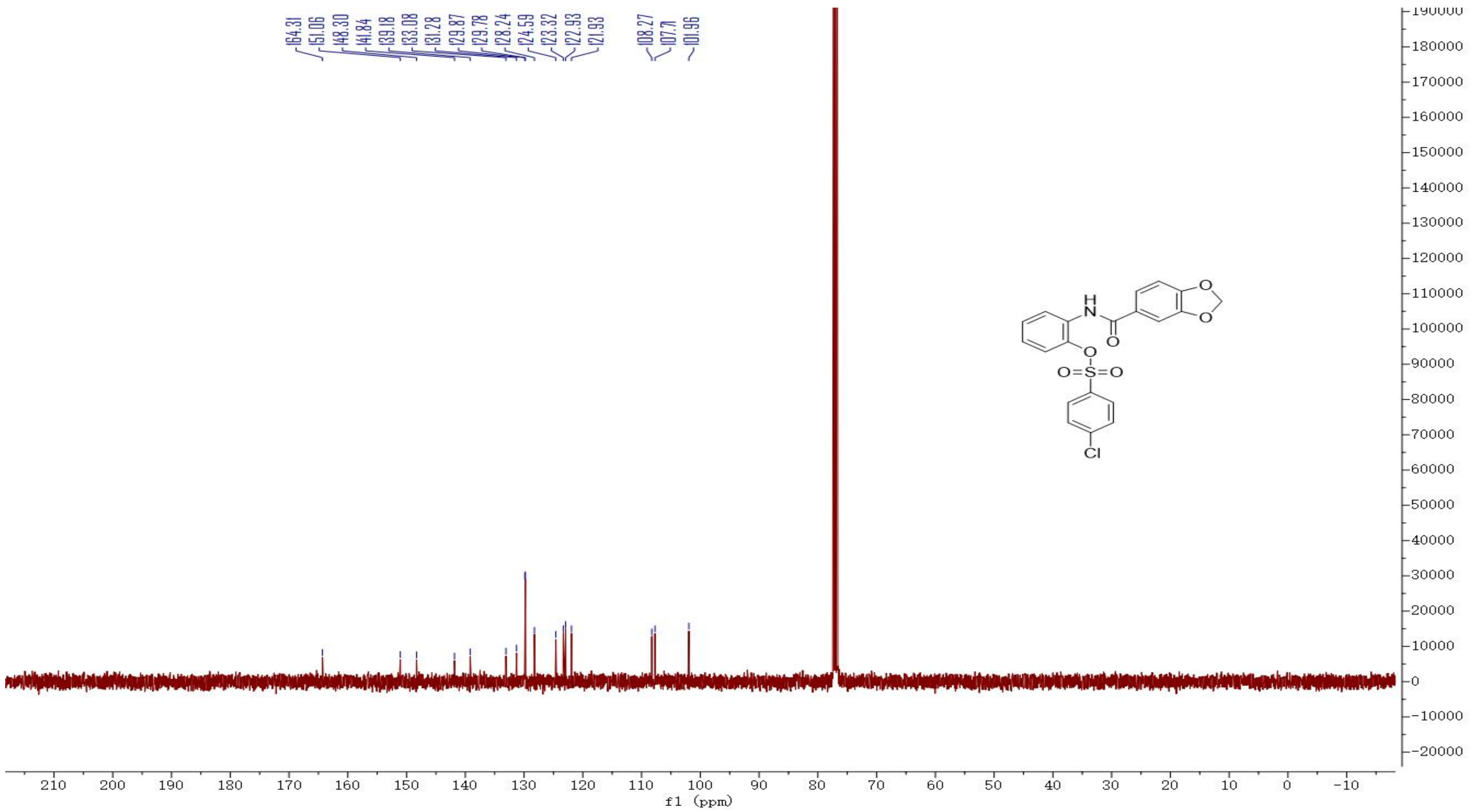

^{13}C NMR of Compound **4j**

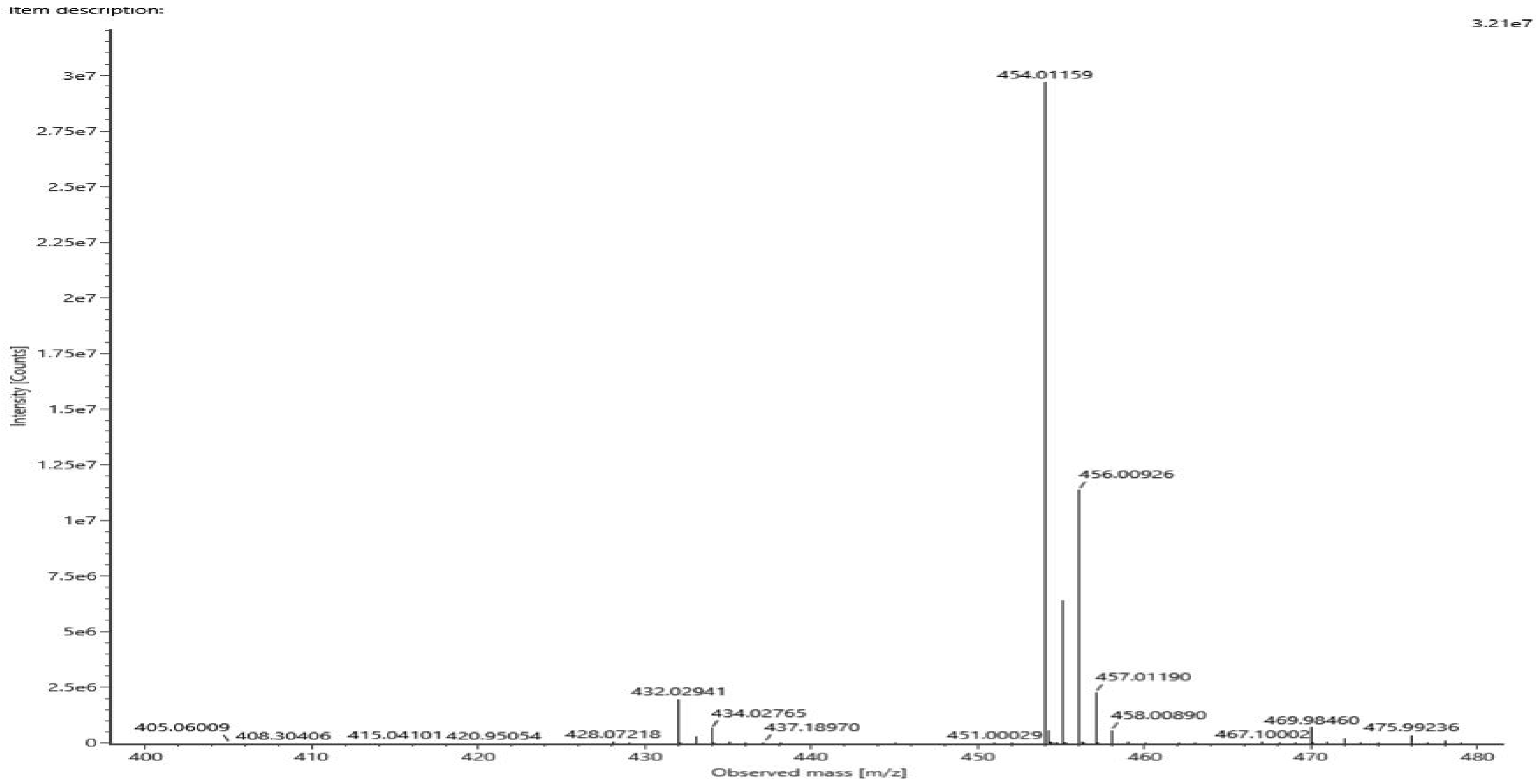

HRMS of Compound **4j**

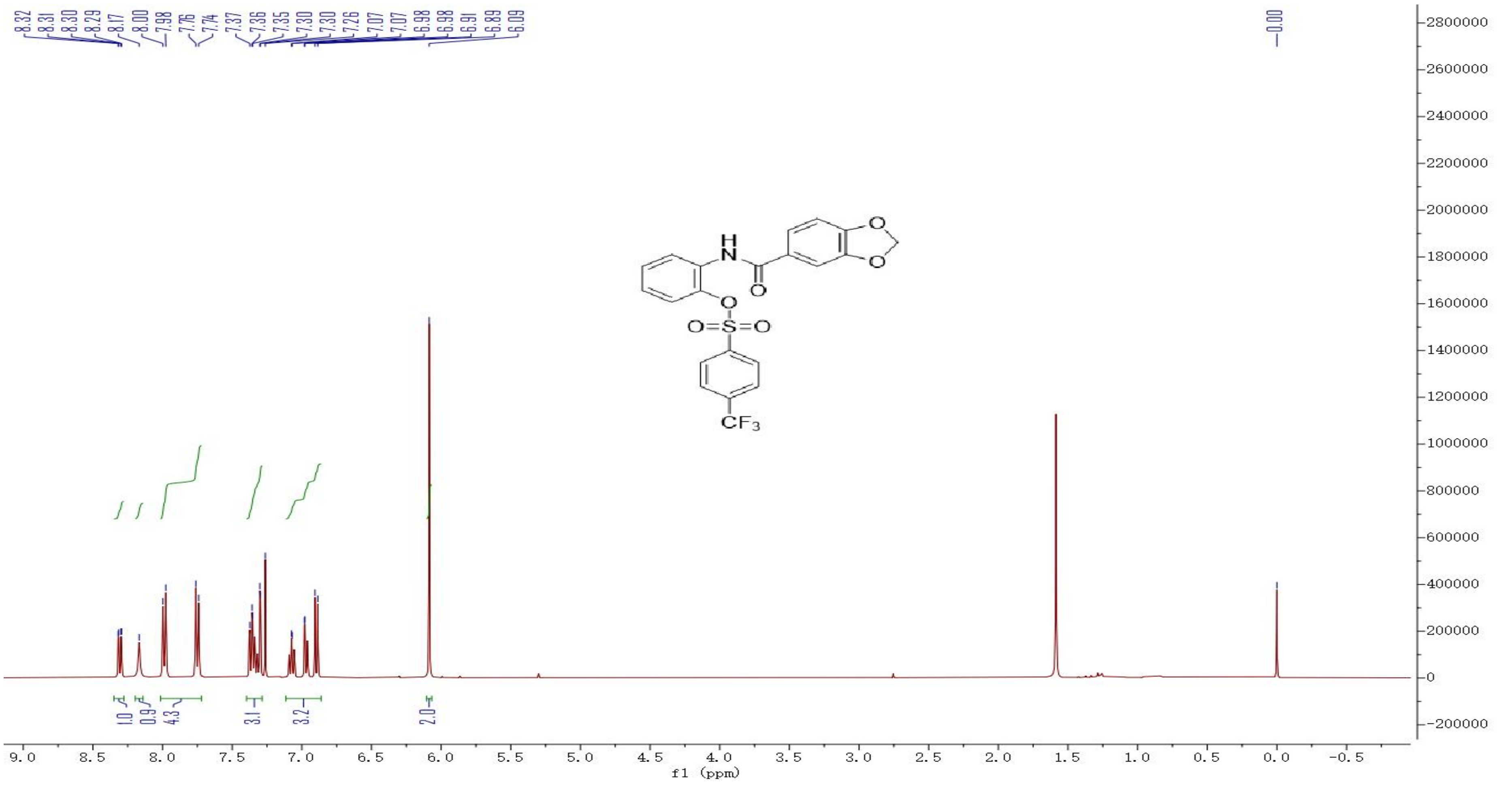

^{1}H NMR of Compound **4k**

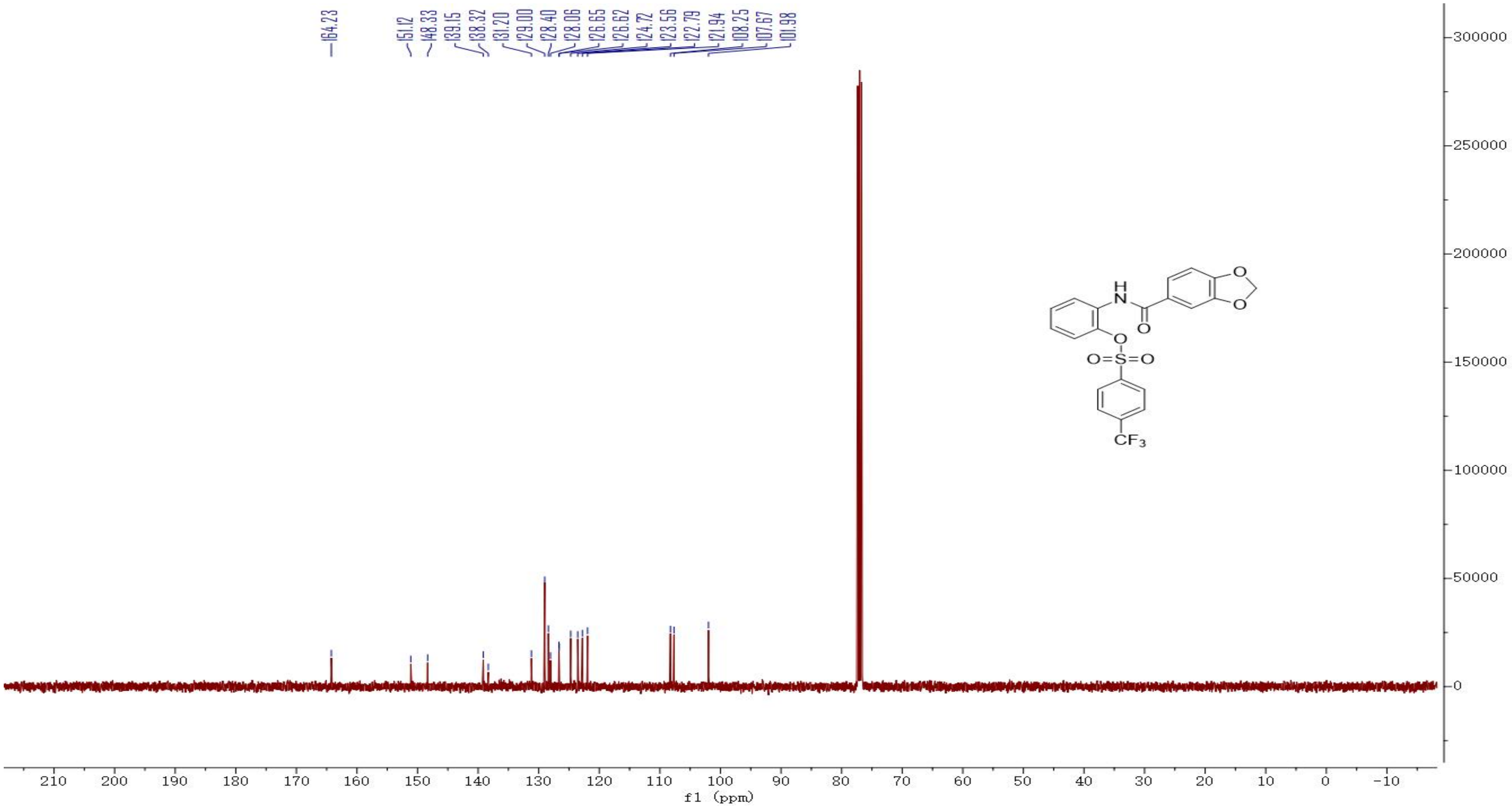

^{13}C NMR of Compound **4k**

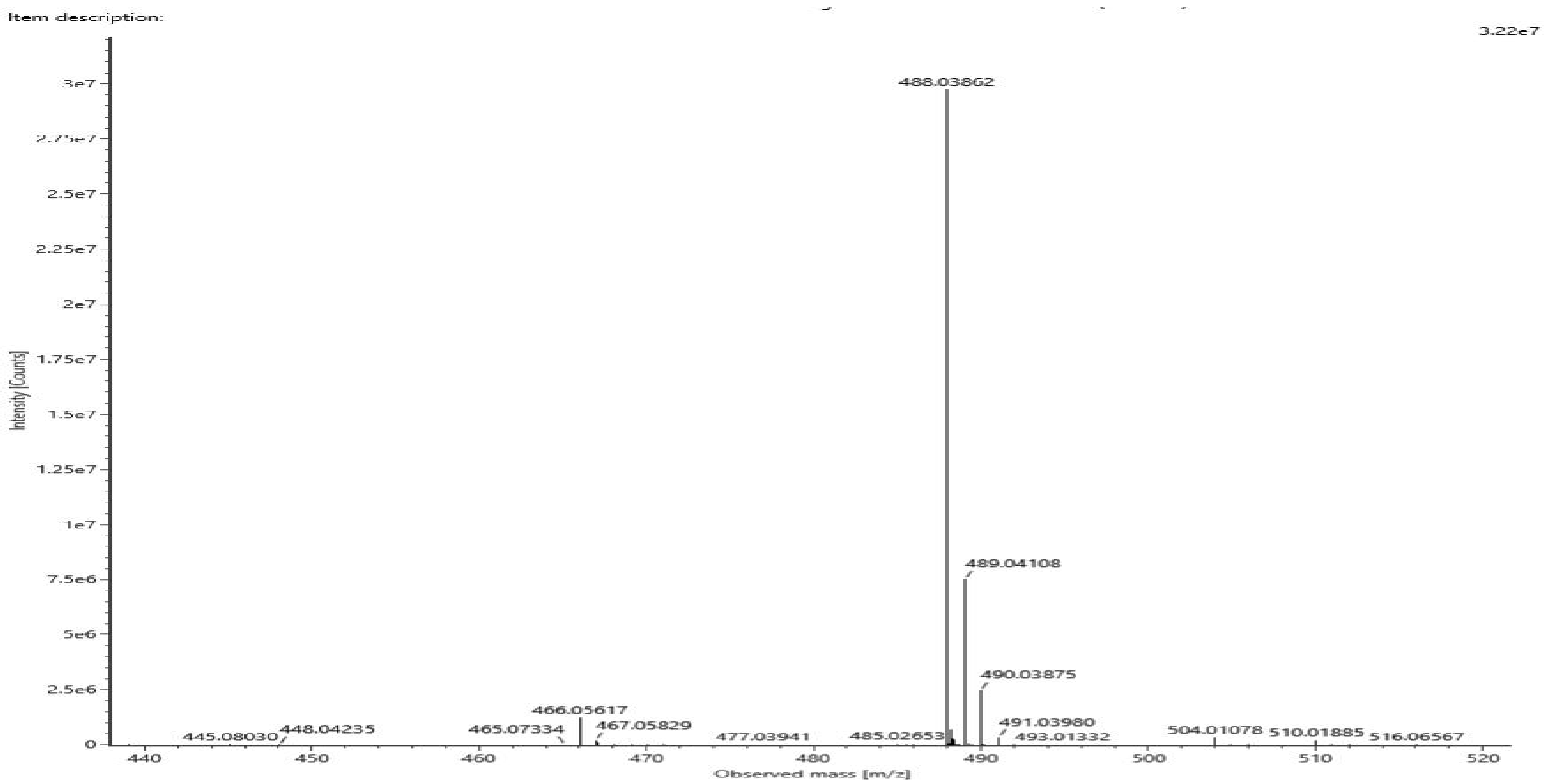

HRMS of Compound **4k**

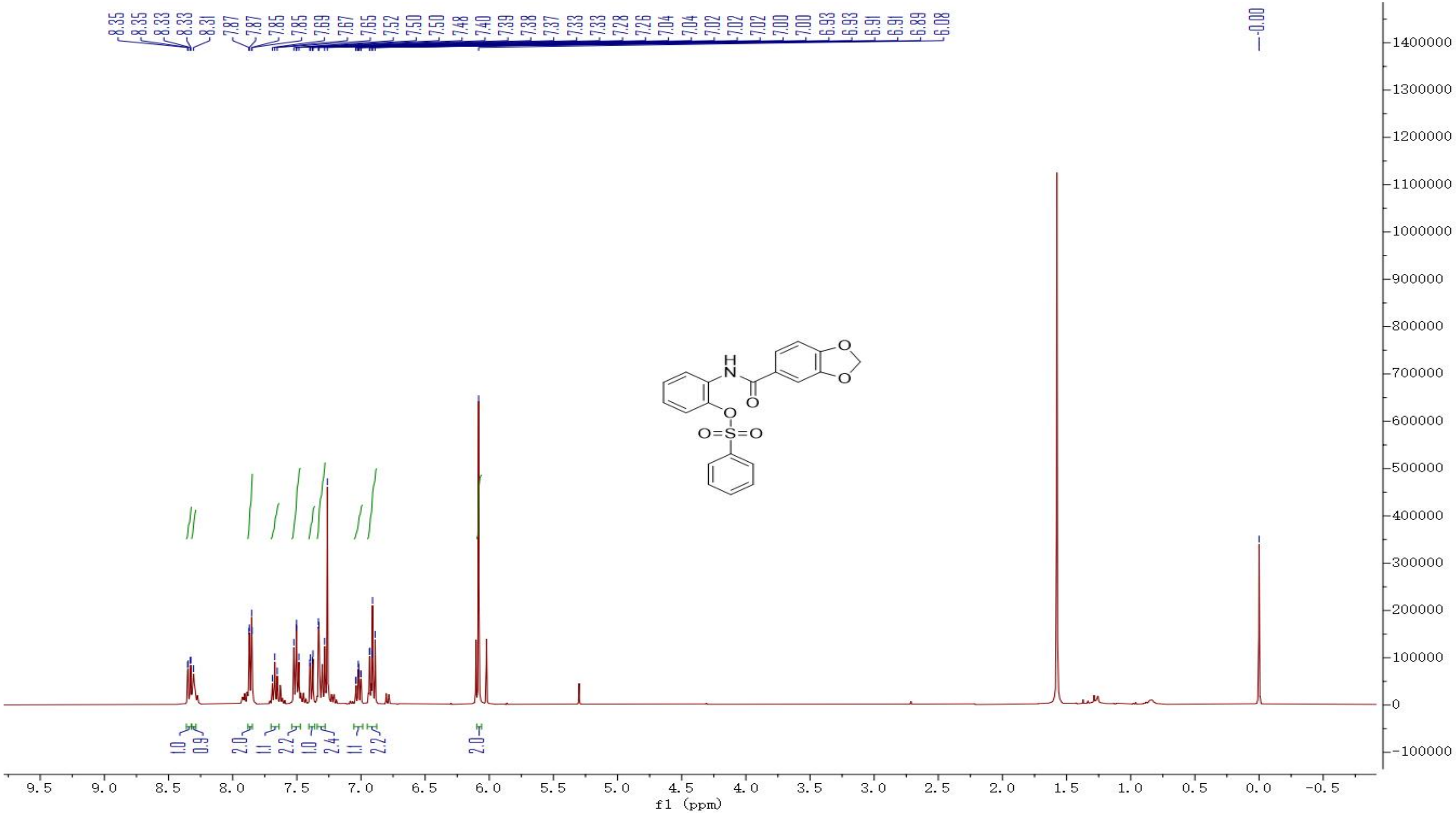

^{1}H NMR of Compound **4l**

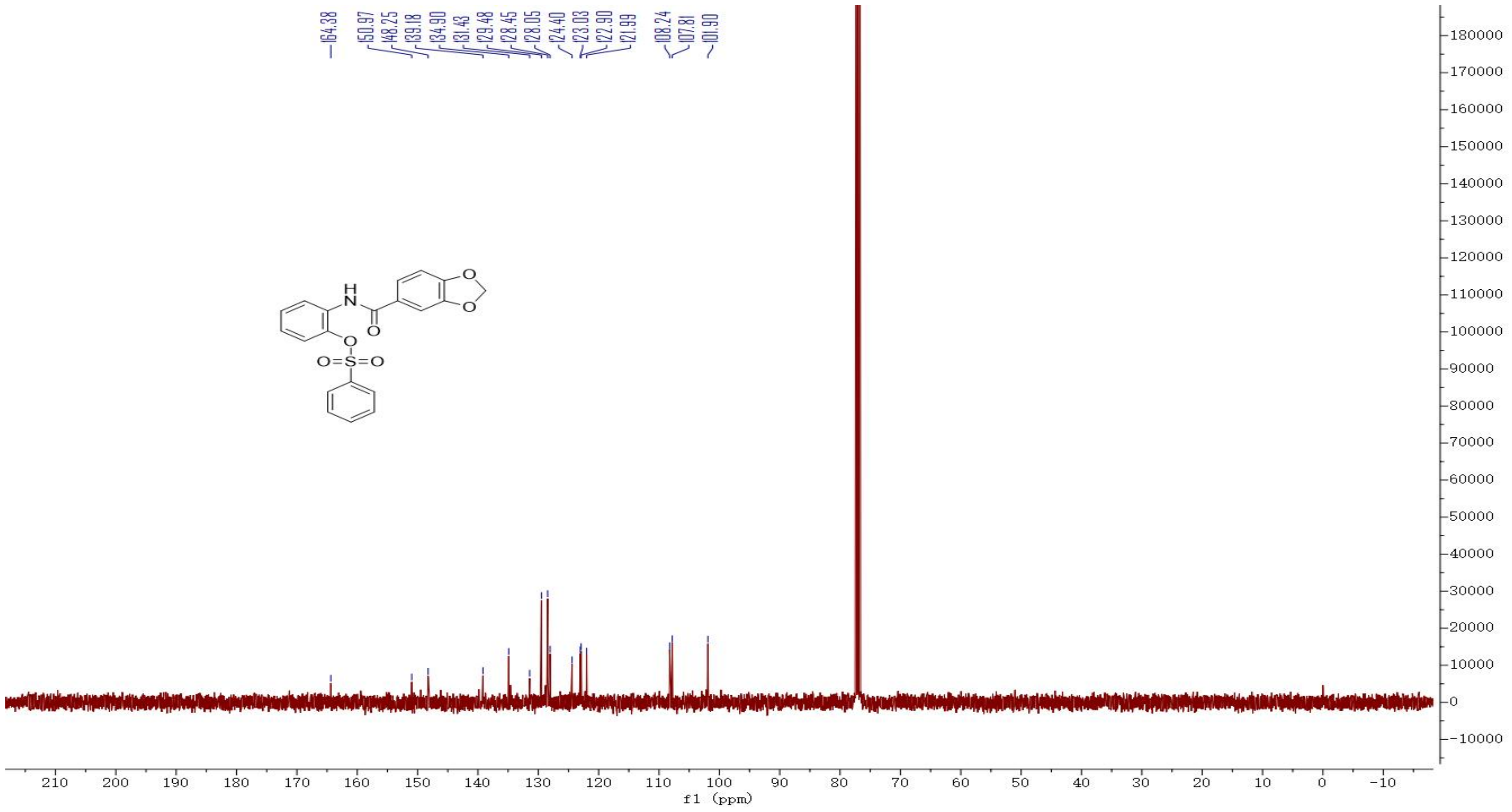

^{13}C NMR of Compound **4l**

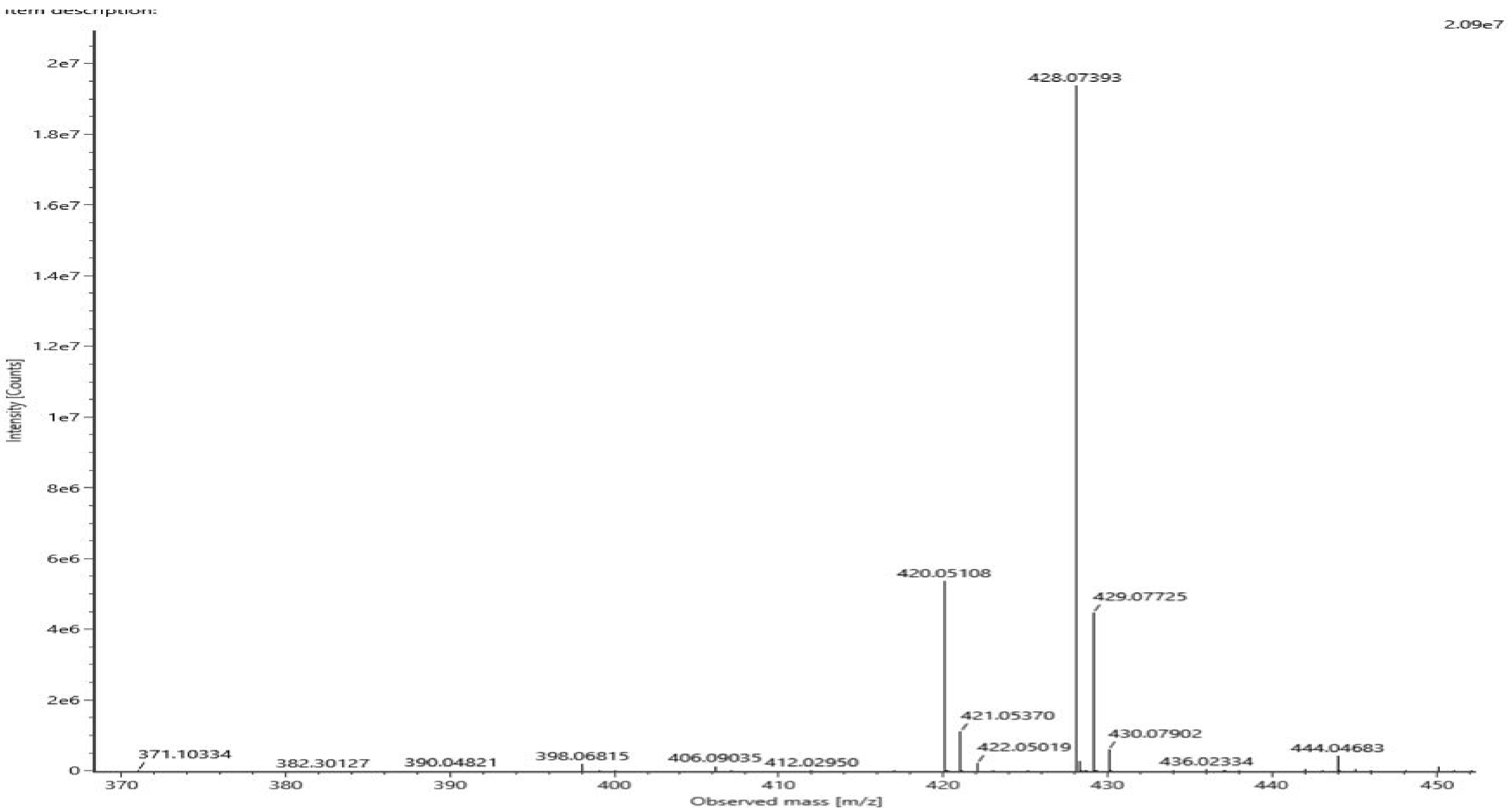

HRMS of Compound **4l**

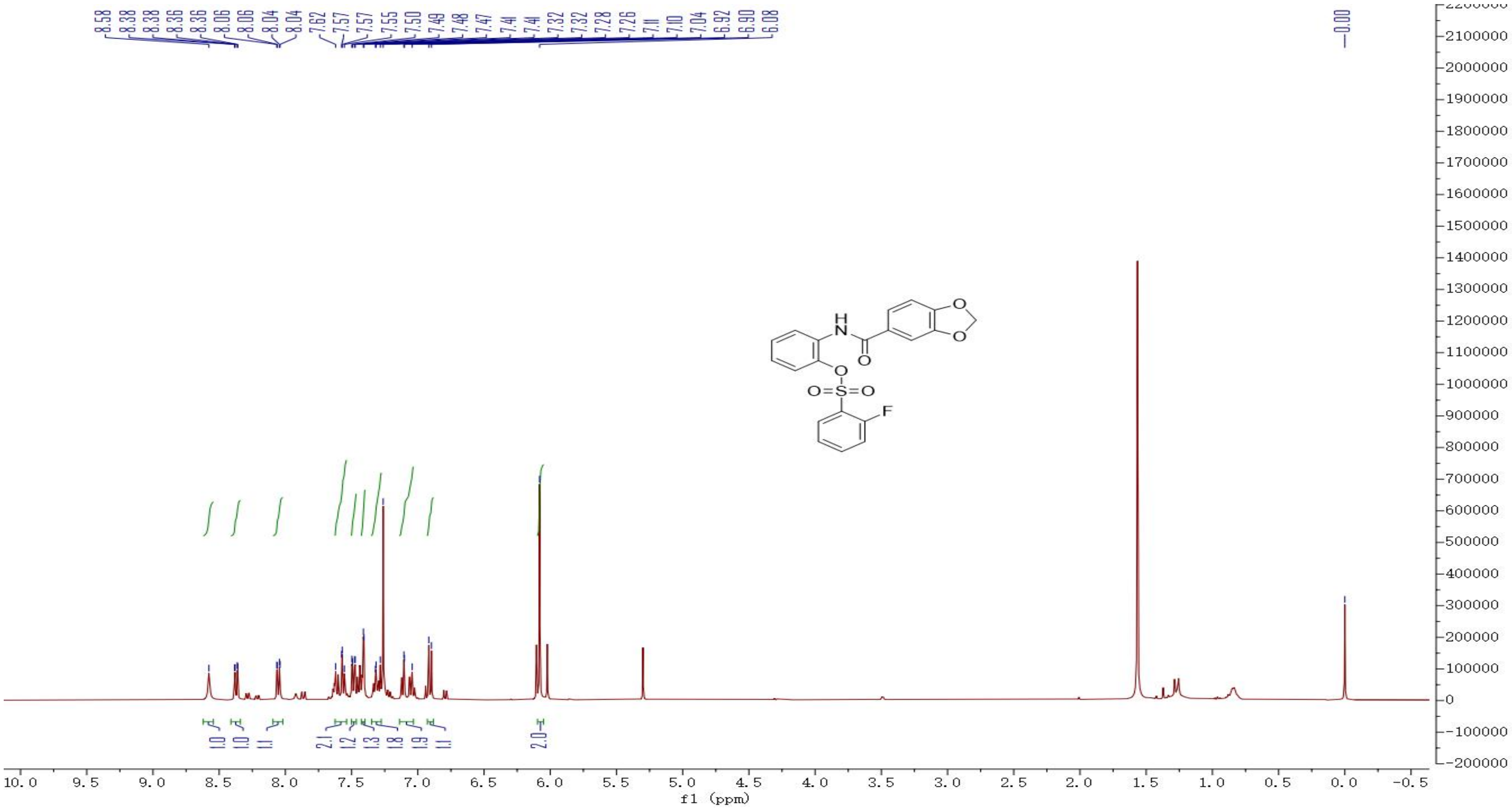

^{1}H NMR of Compound **4m**

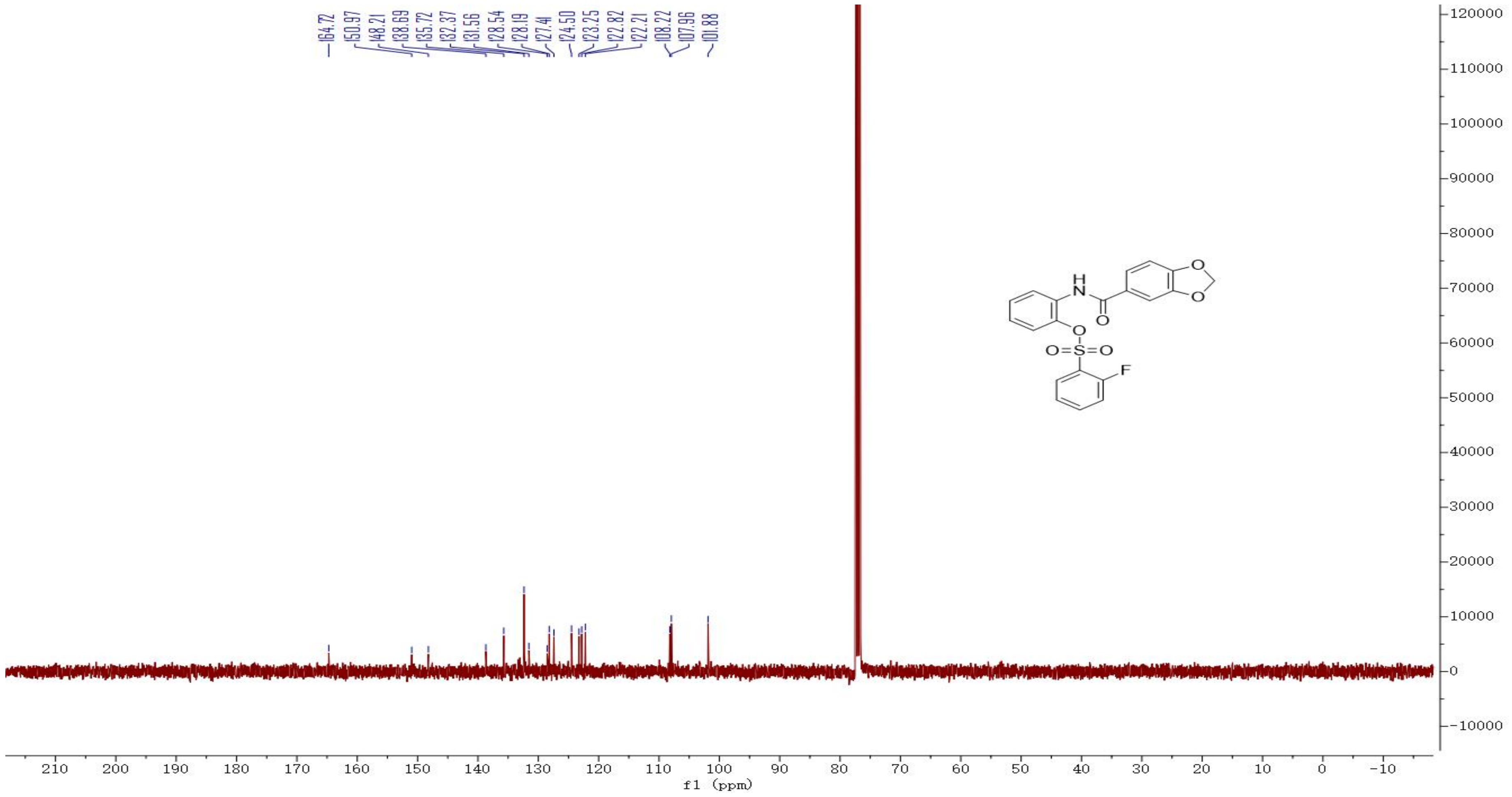

^{13}C NMR of Compound **4m**

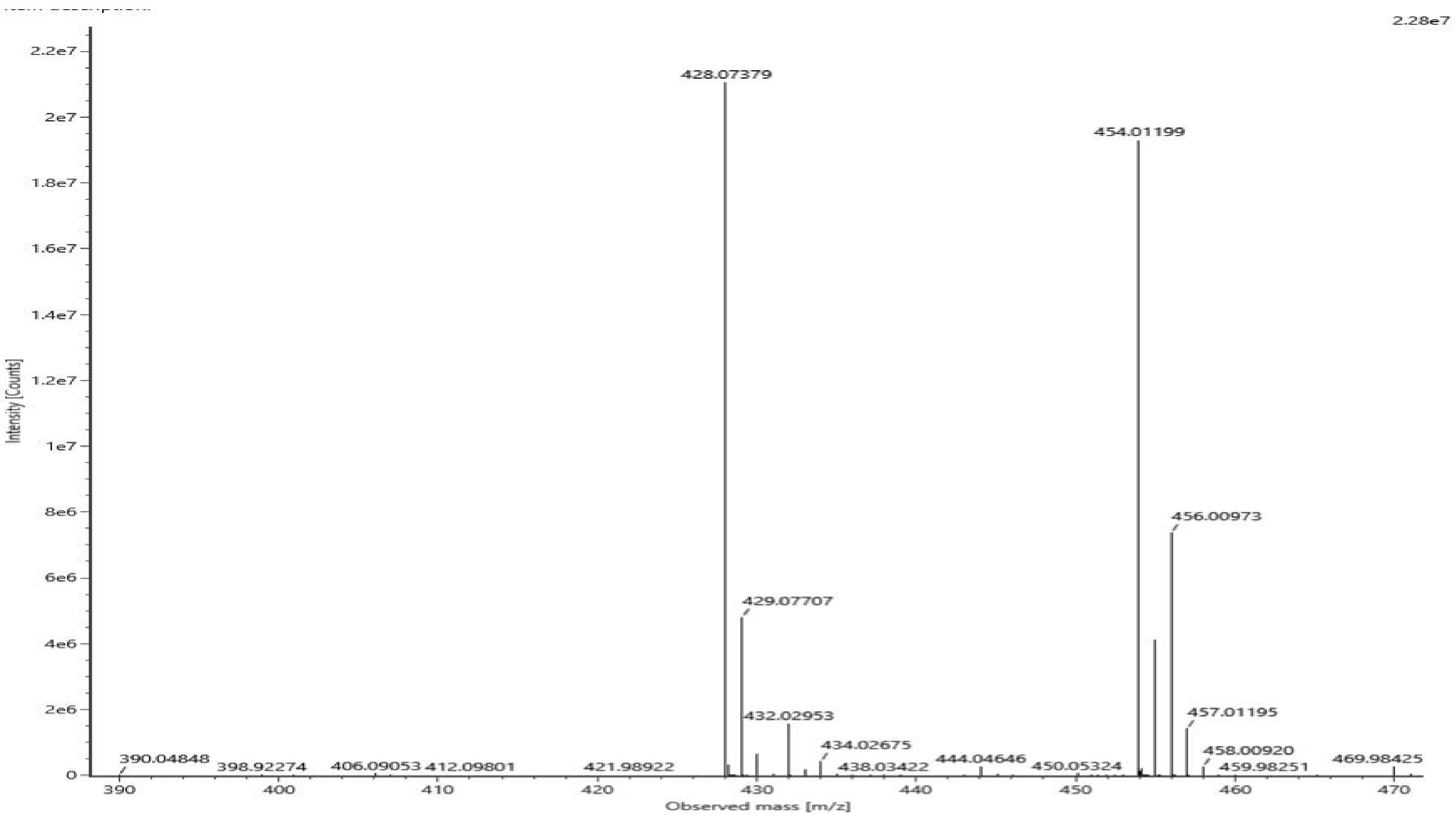

HRMS of Compound **4m**

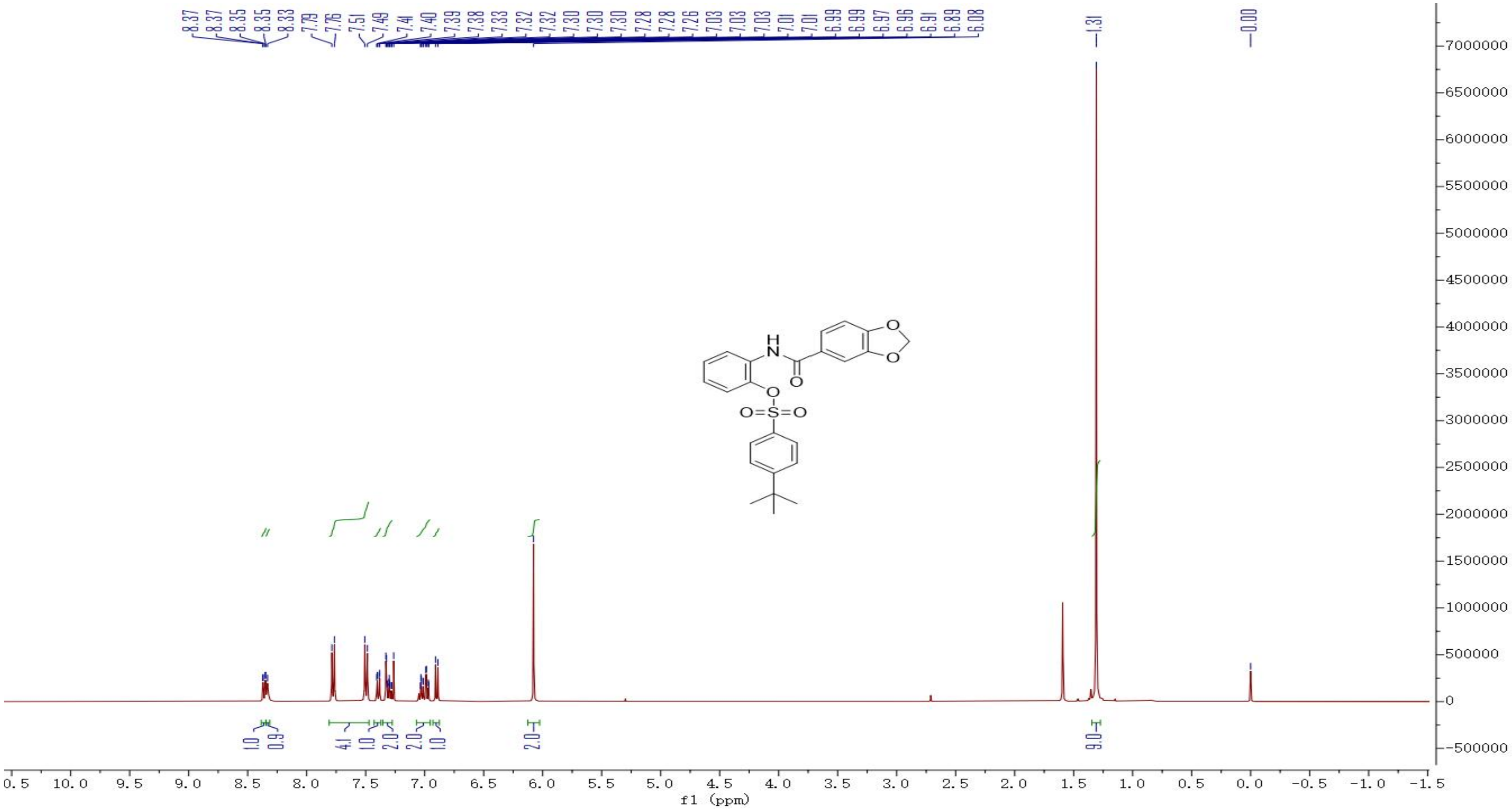

^{1}H NMR of Compound **4n**

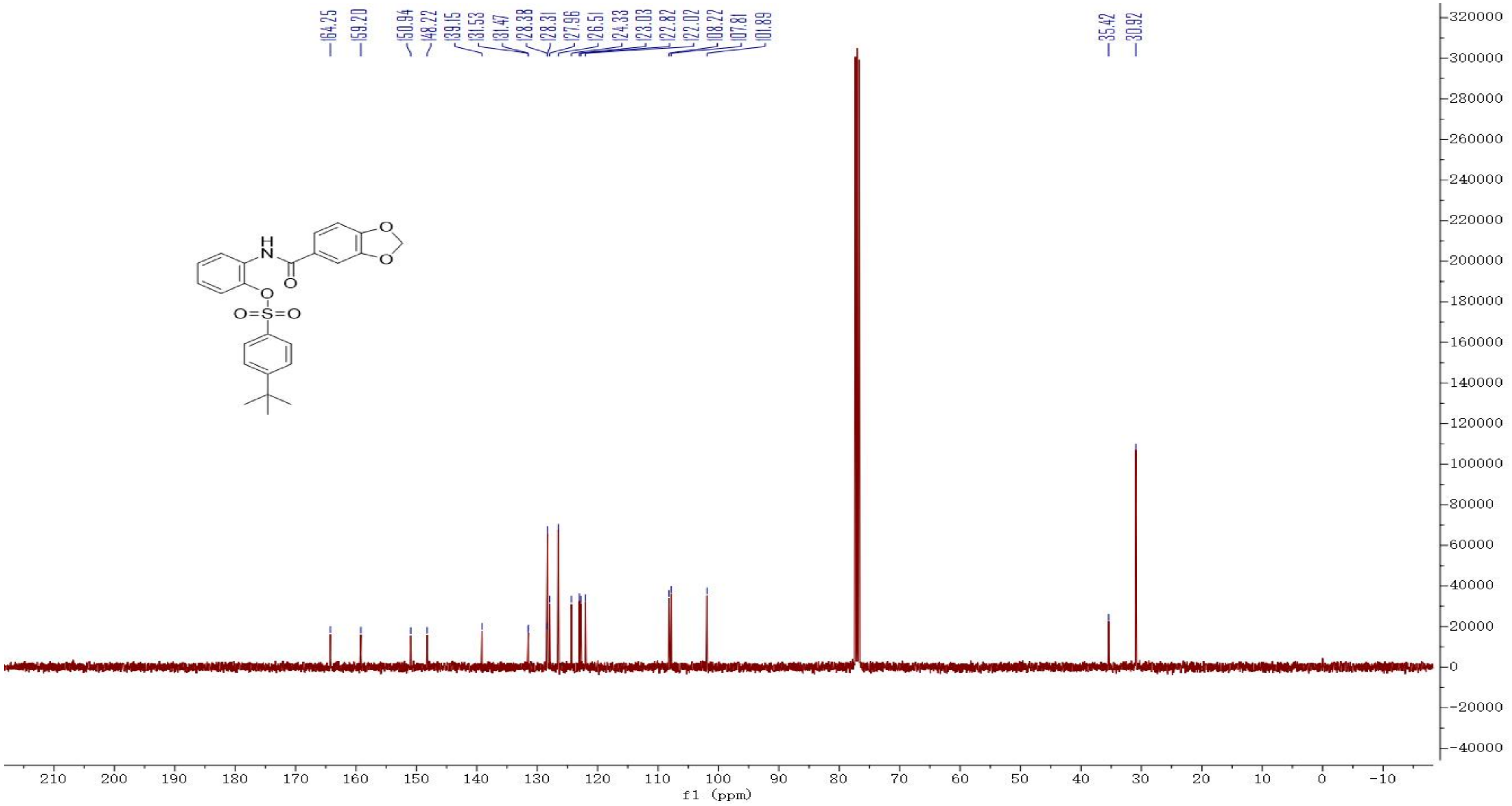

^{13}C NMR of Compound **4n**

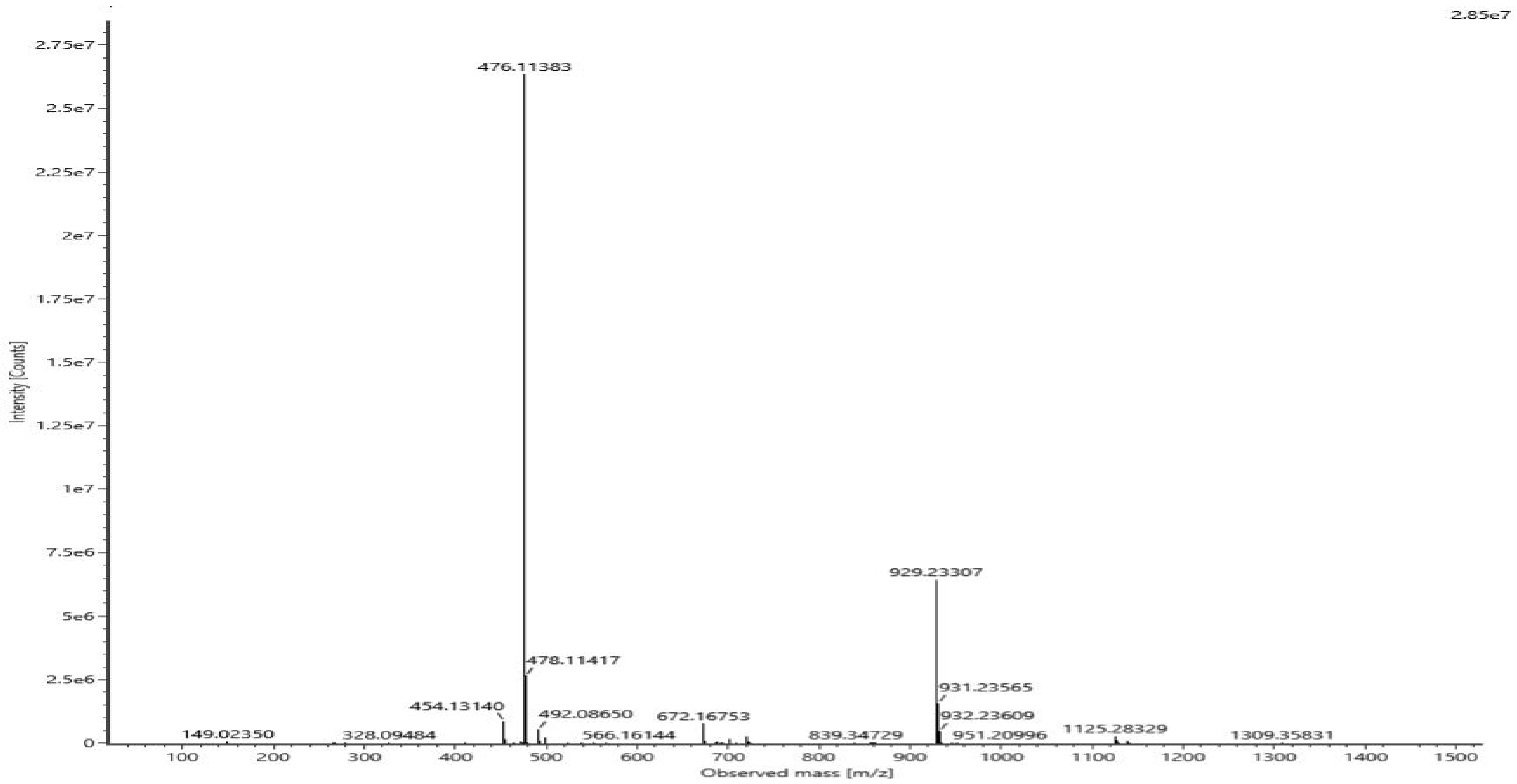

HRMS of Compound **4n**

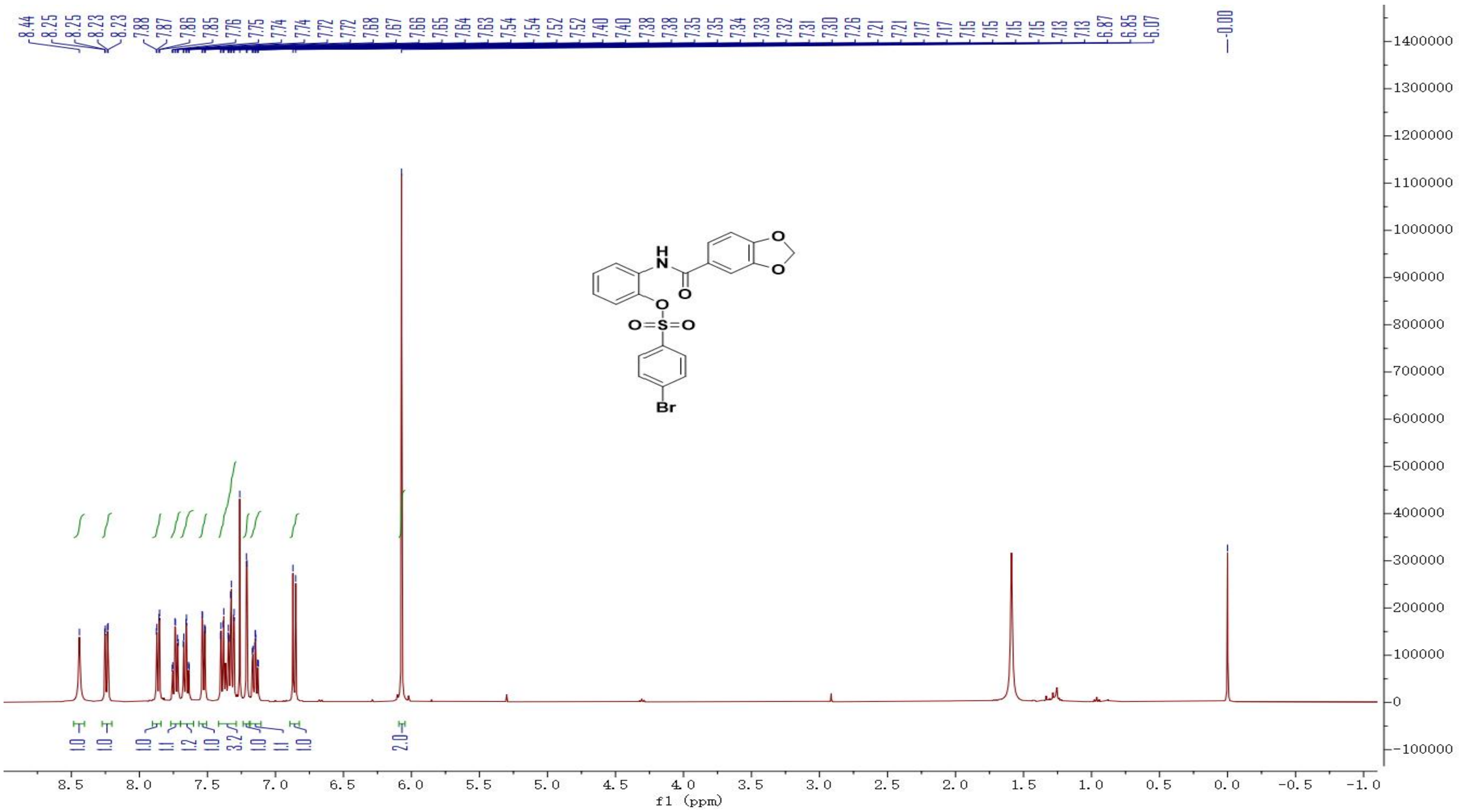

^{1}H NMR of Compound **4o**

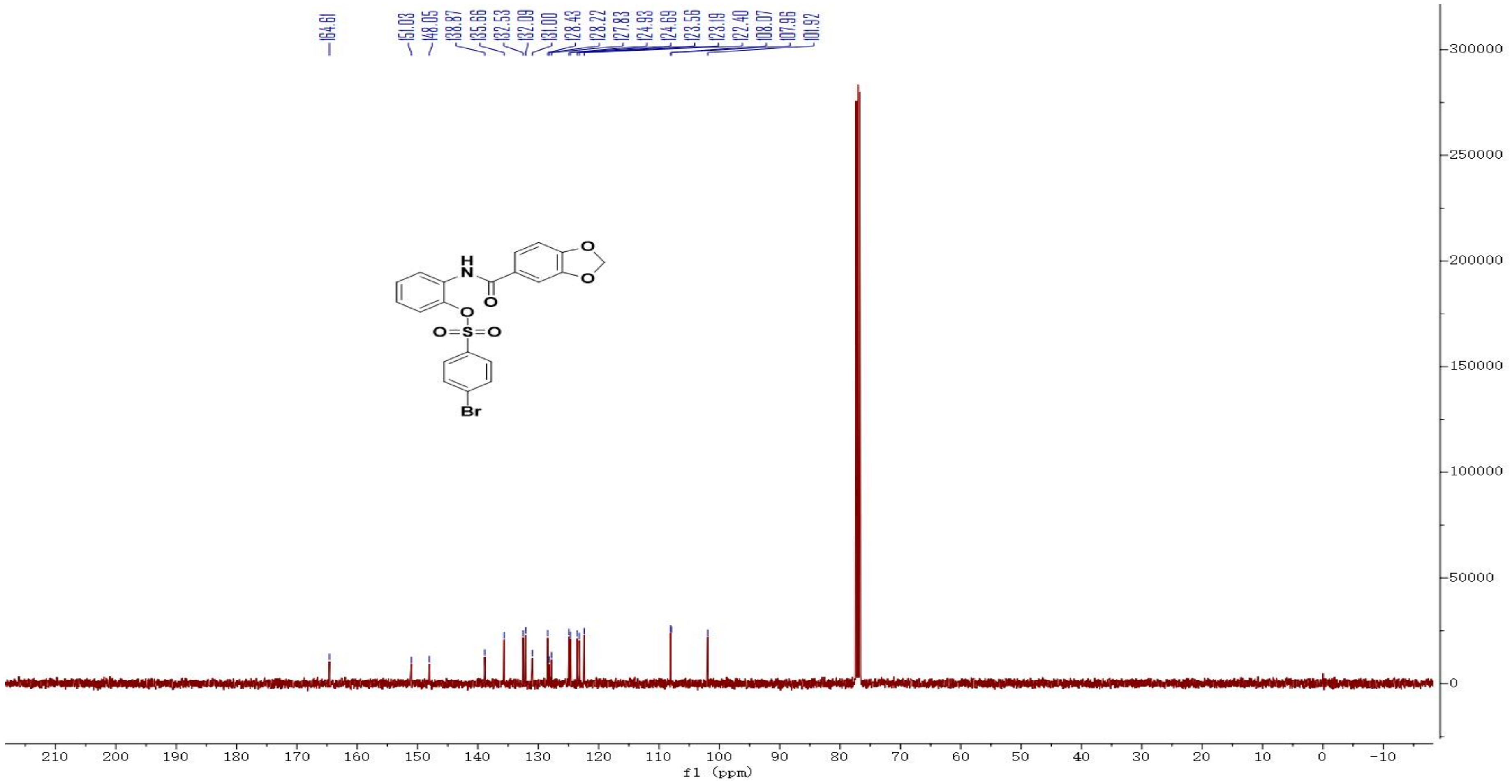

^{13}C NMR of Compound **4o**

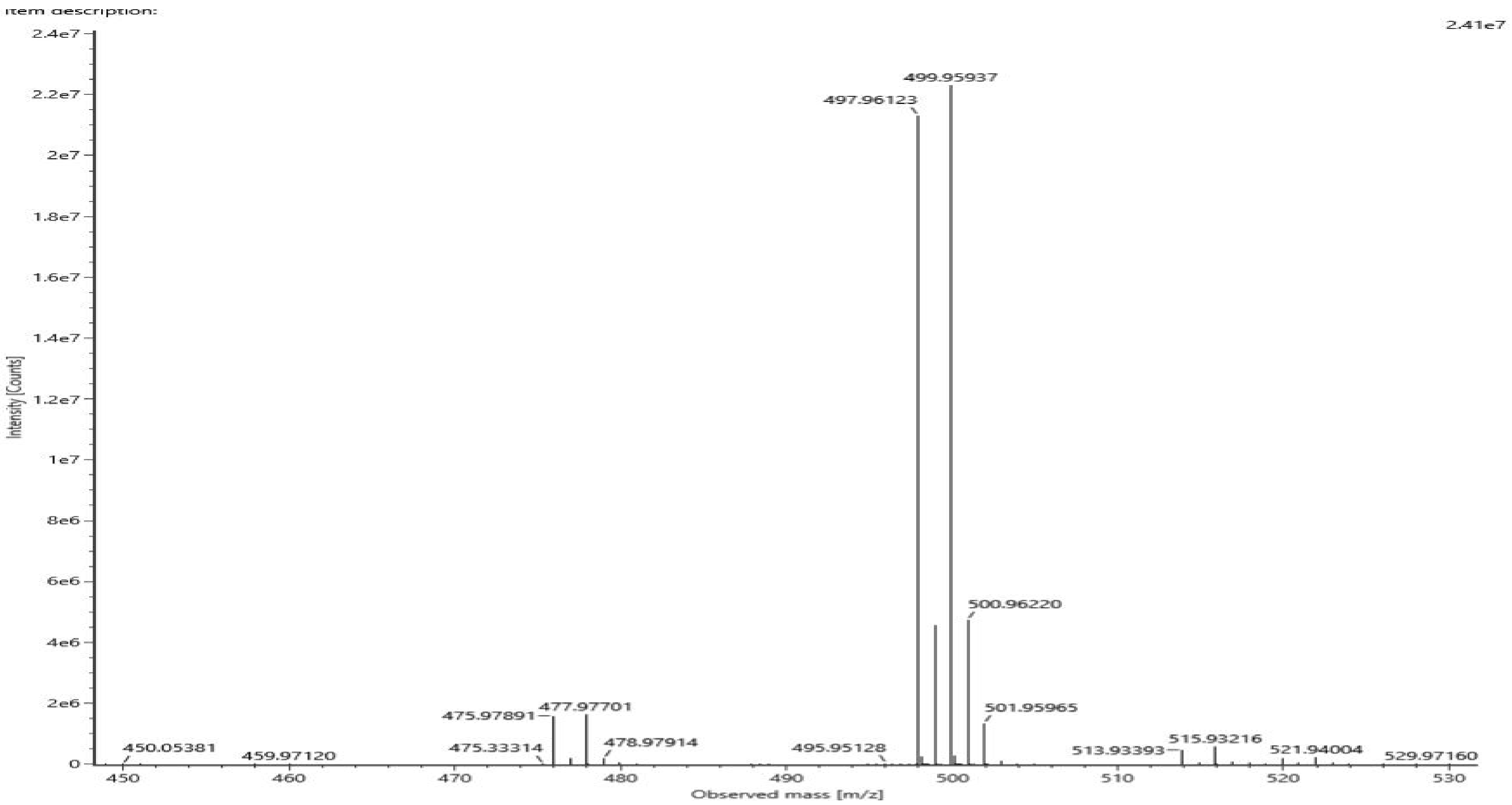

HRMS of Compound **4o**

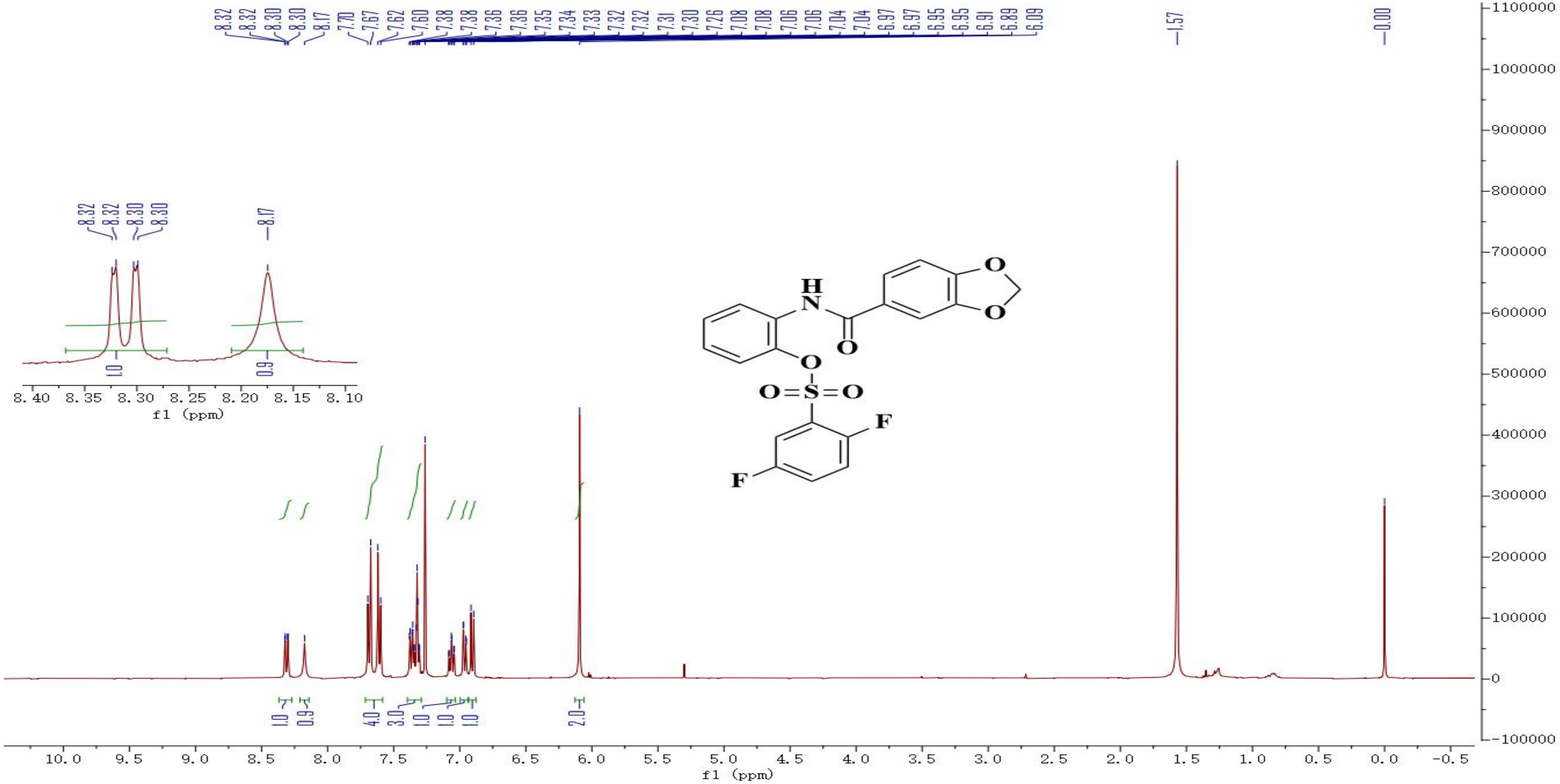

^{1}H NMR of Compound **4p**

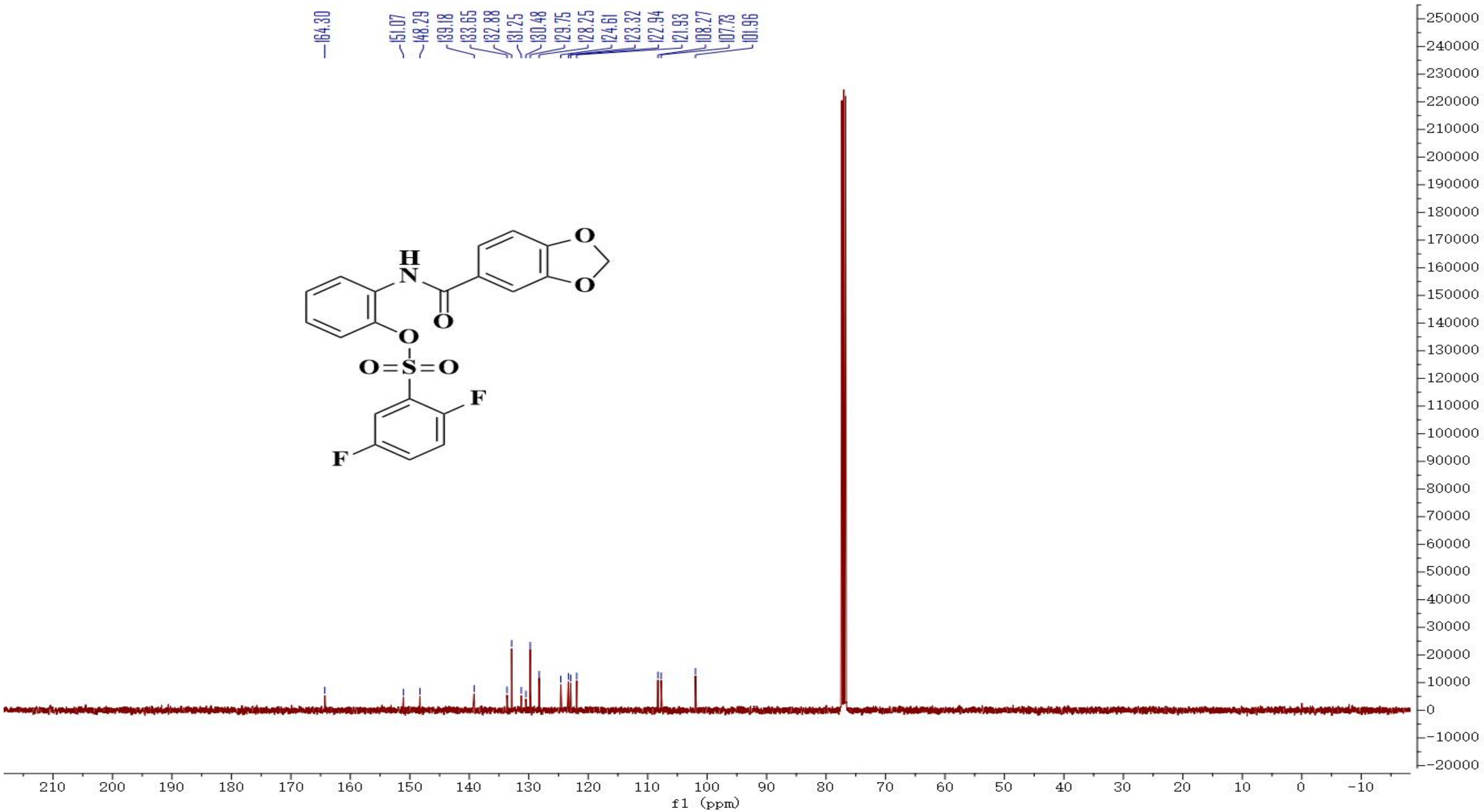

^{13}C NMR of Compound **4p**

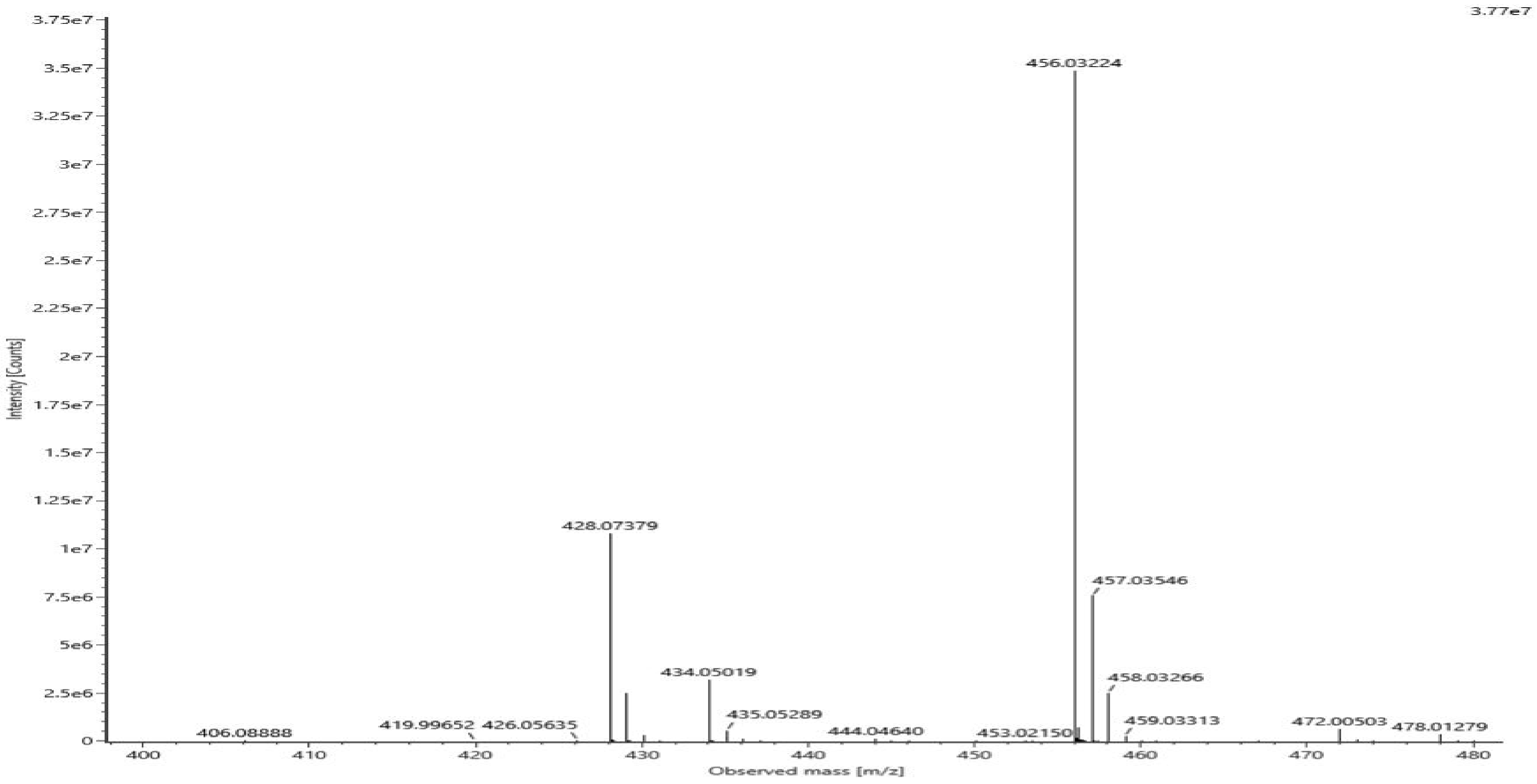

HRMS of Compound **4p**

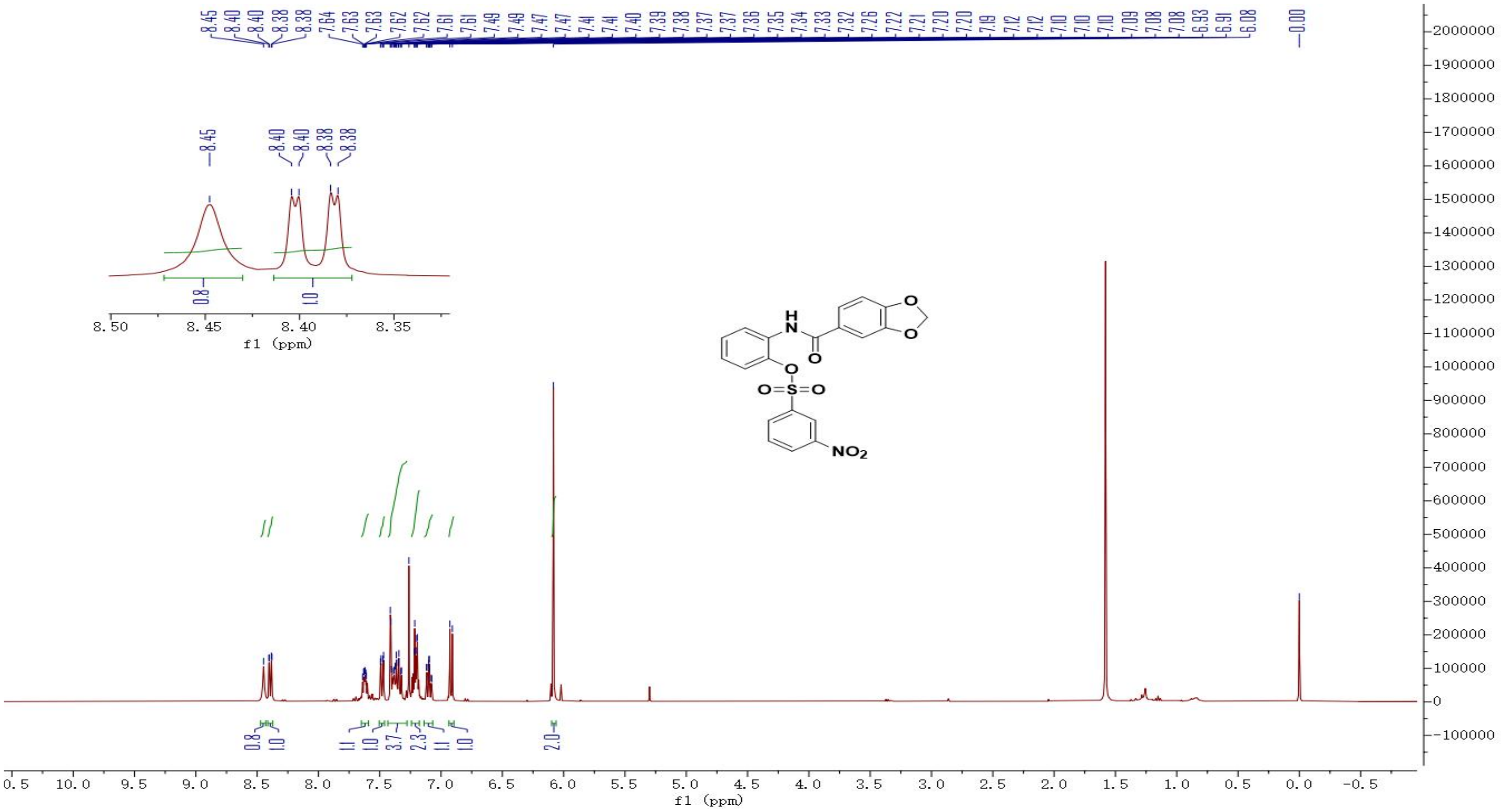

^{1}H NMR of Compound **4q**

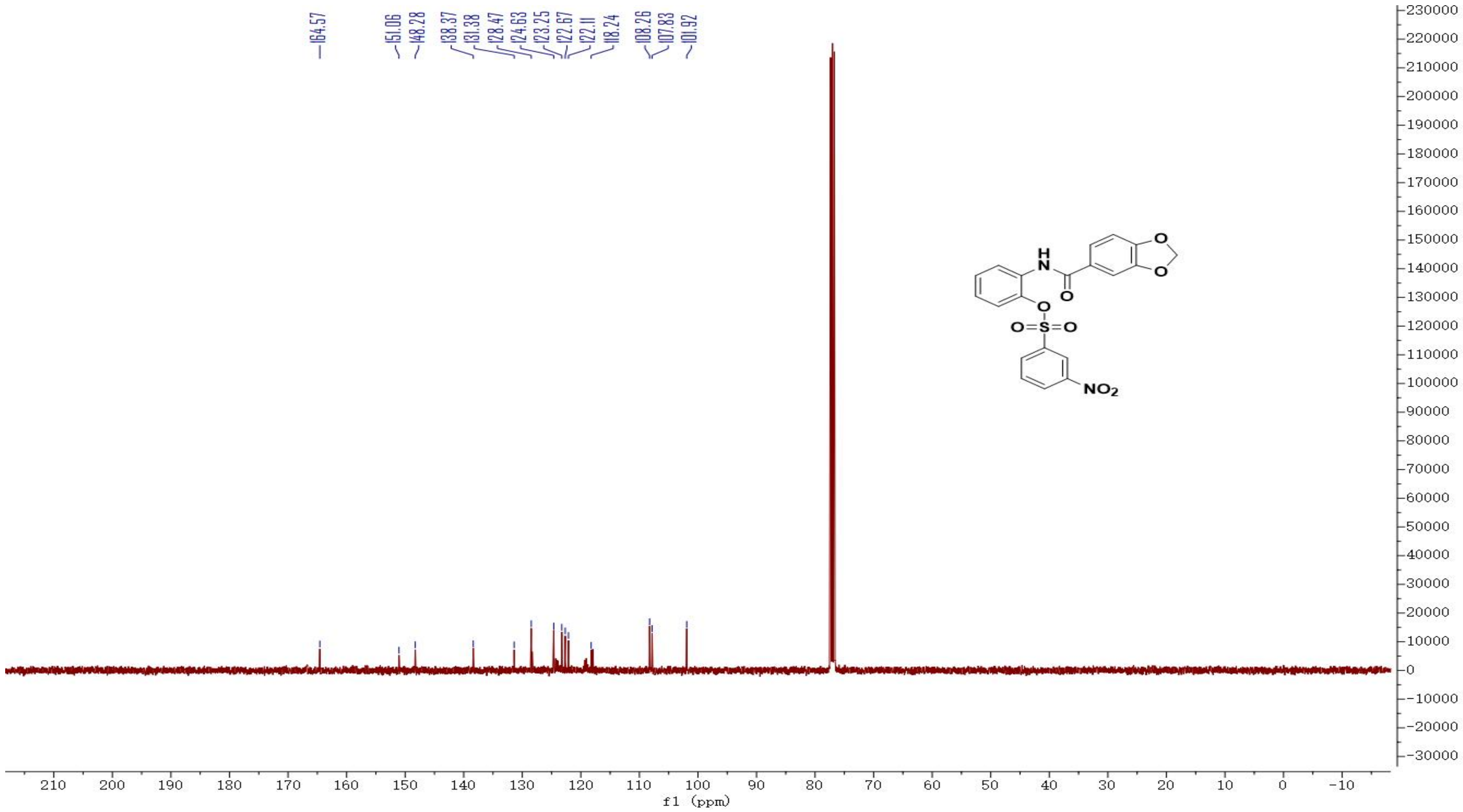

^{13}C NMR of Compound **4q**

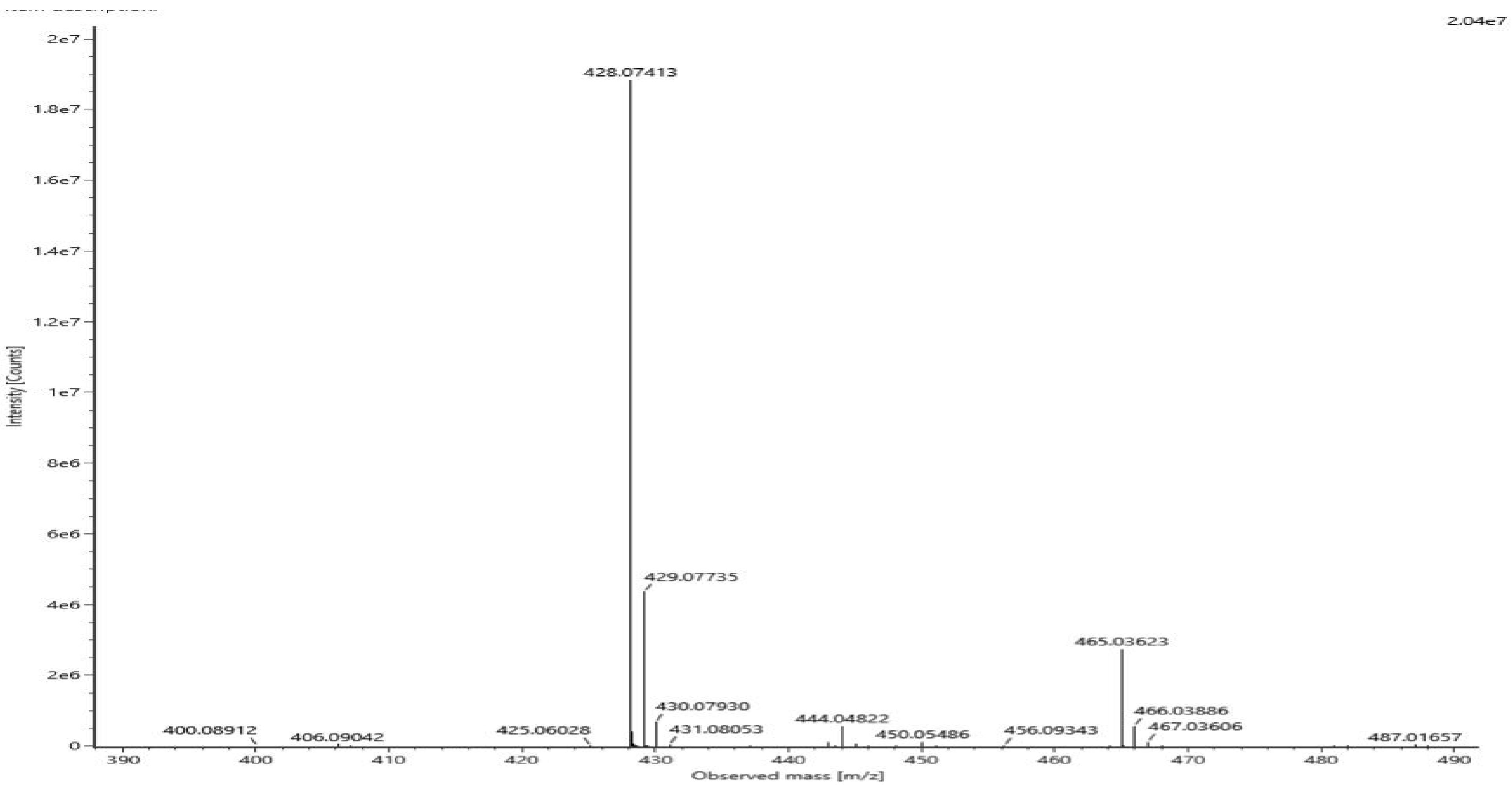

HRMS of Compound **4q**

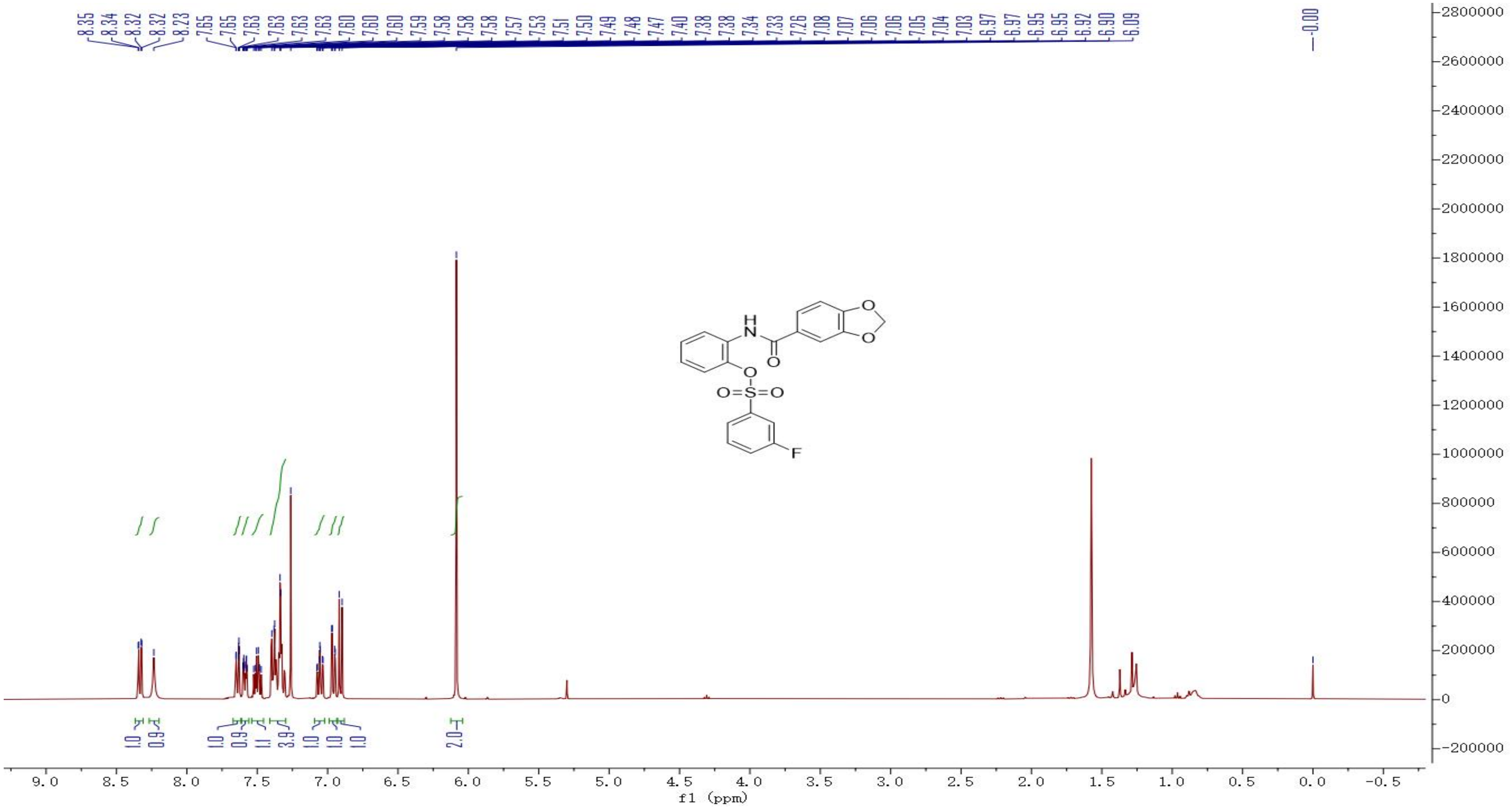

^{1}H NMR of Compound **4r**

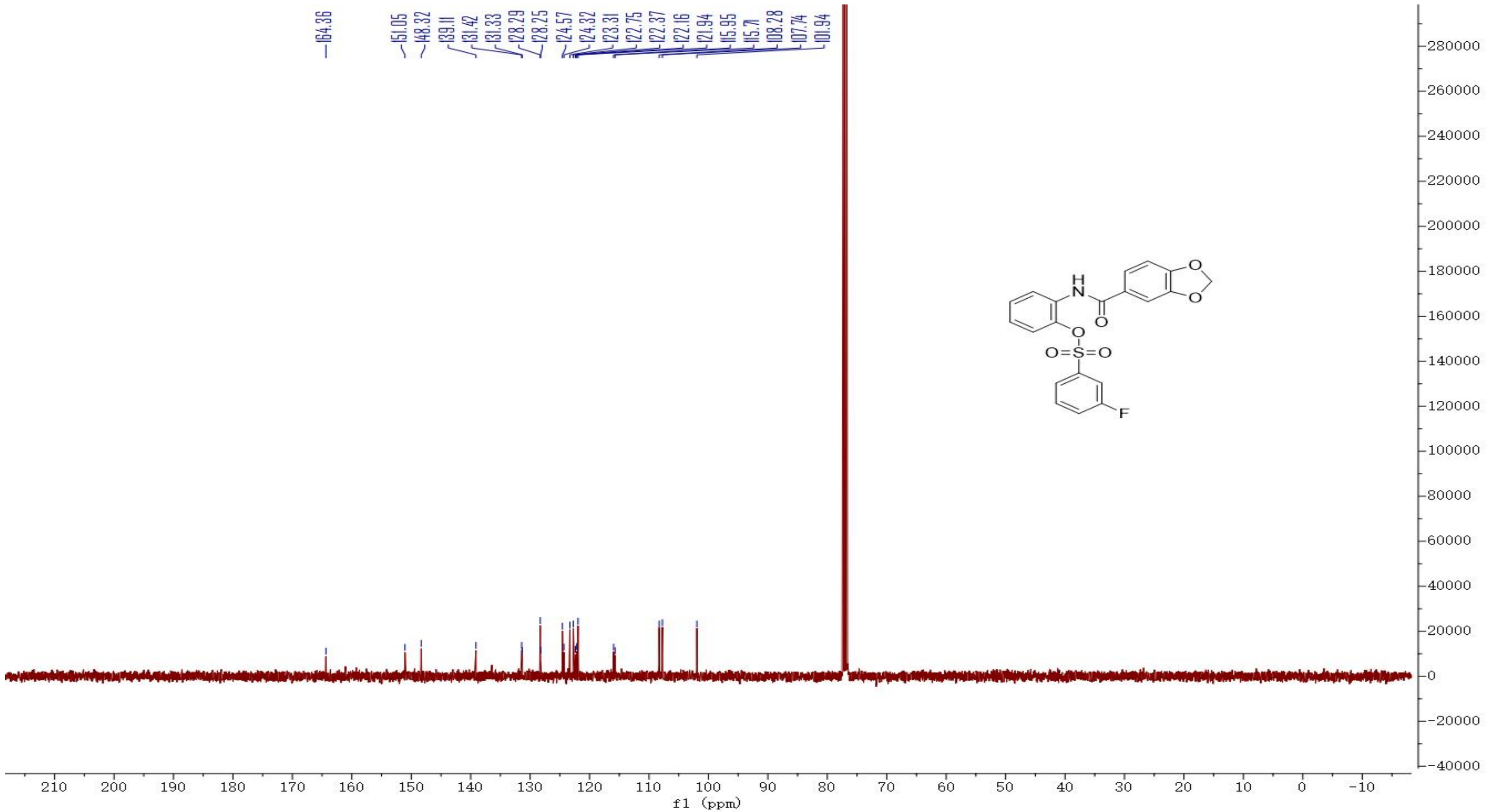

^{13}C NMR of Compound **4r**

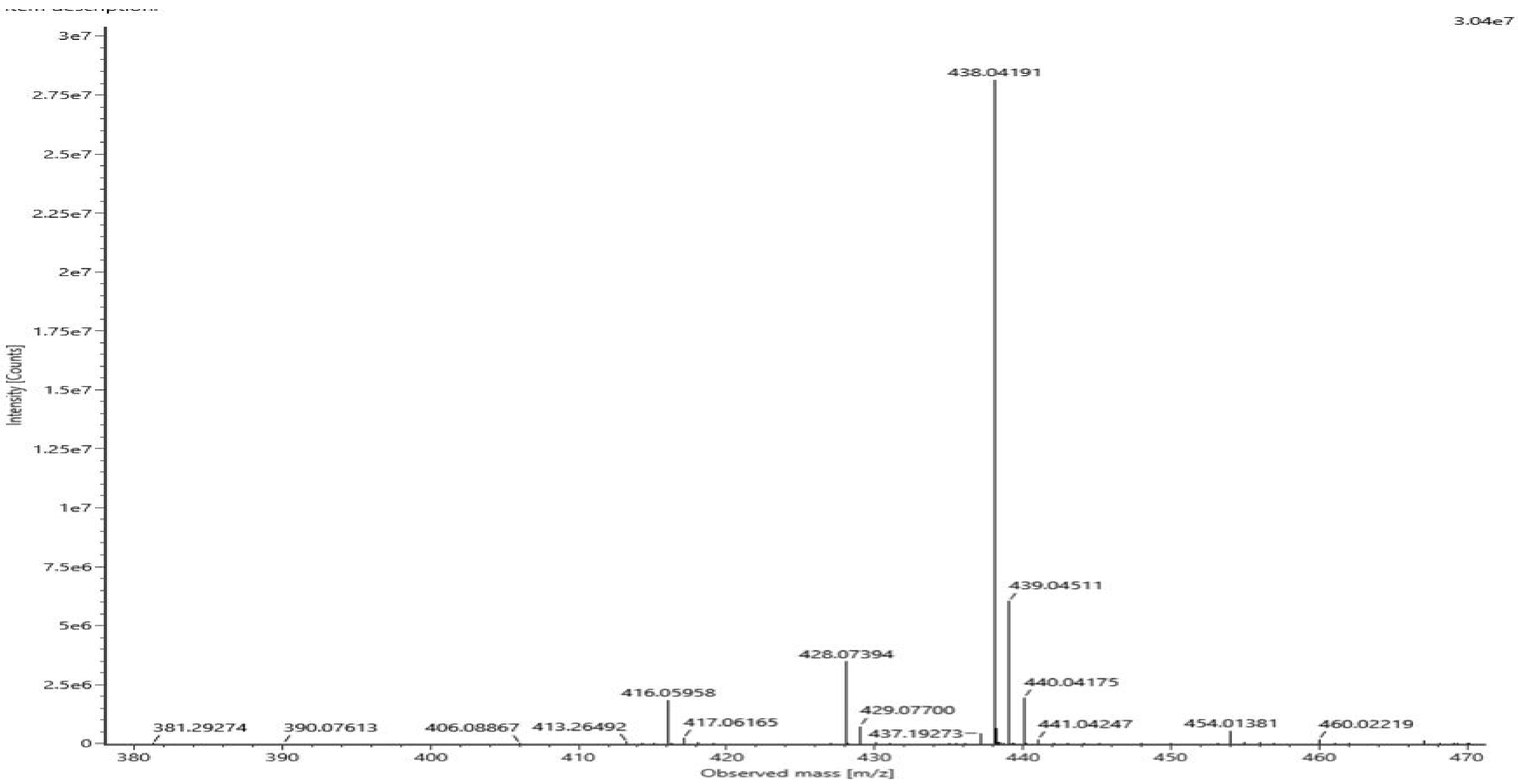

HRMS of Compound **4r**

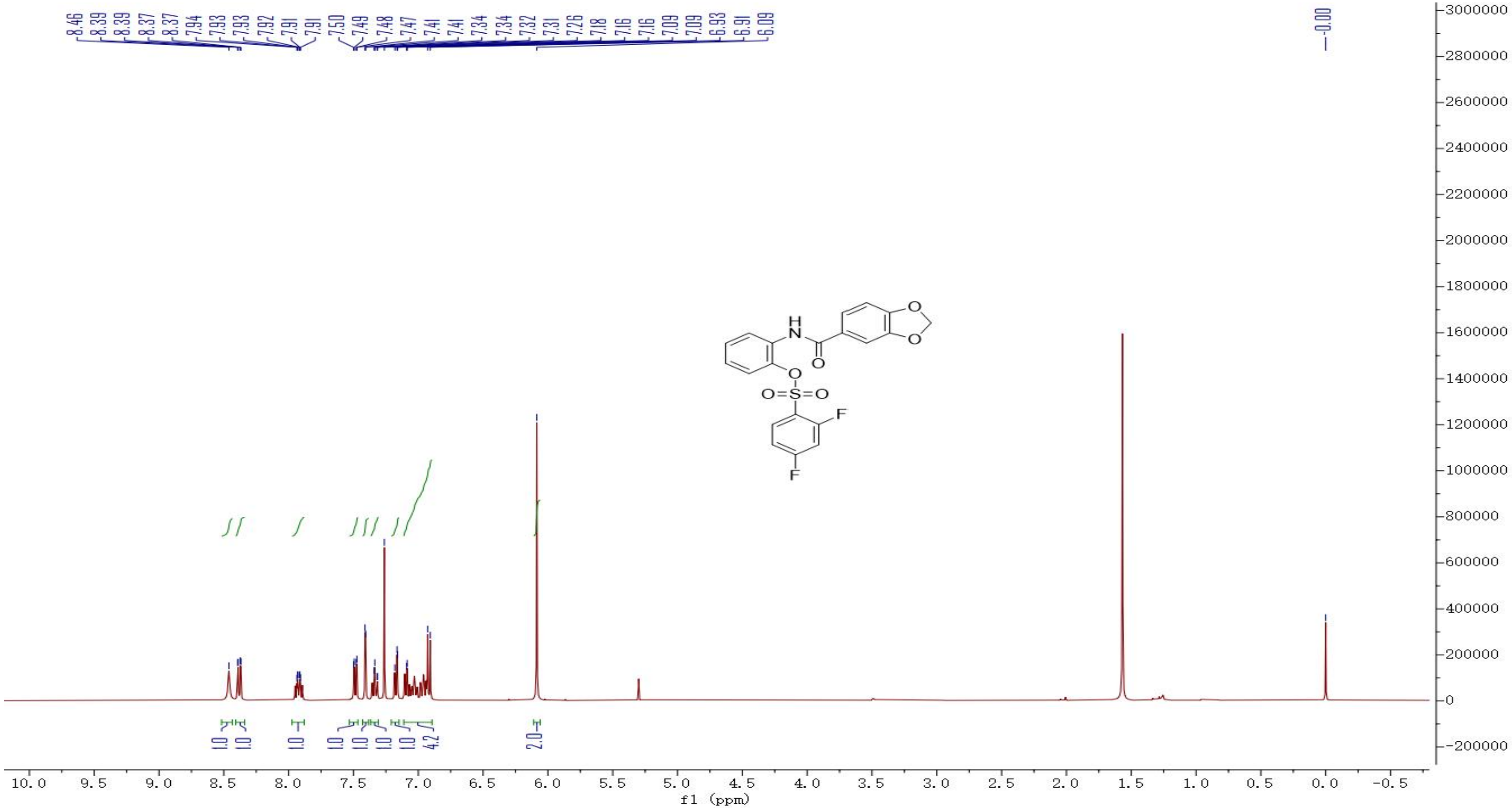

^{1}H NMR of Compound **4s**

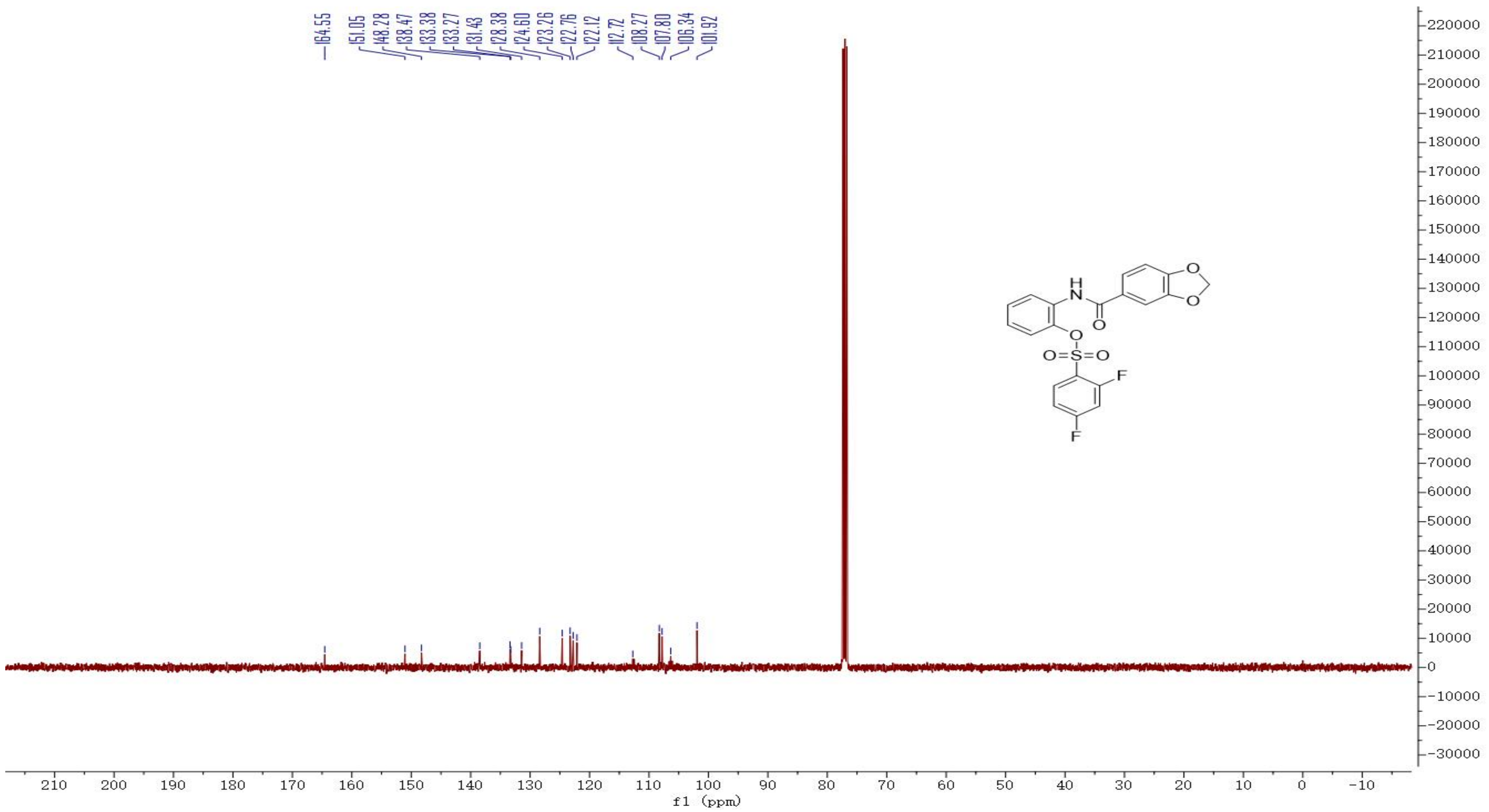

^{13}C NMR of Compound **4s**

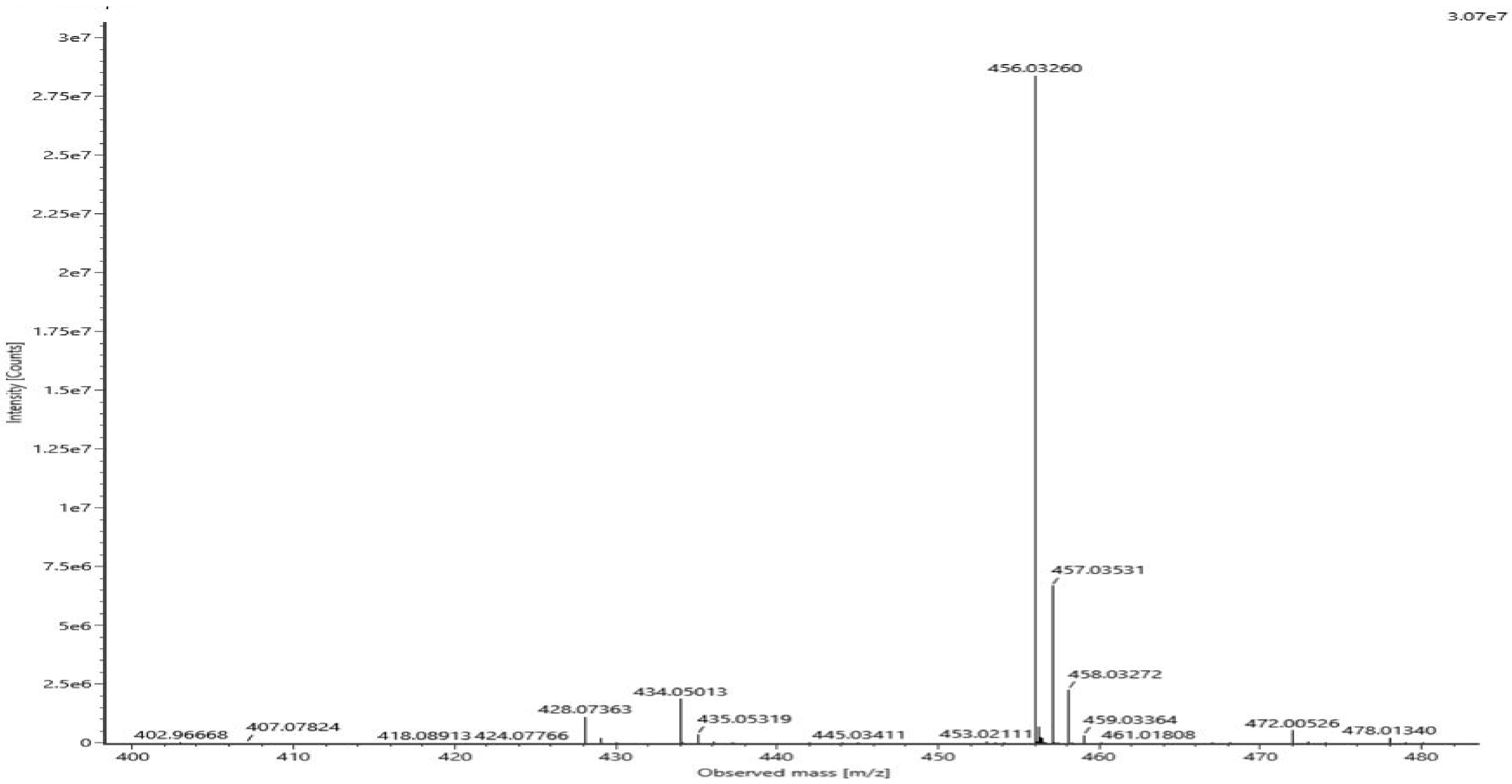

HRMS of Compound **4s**

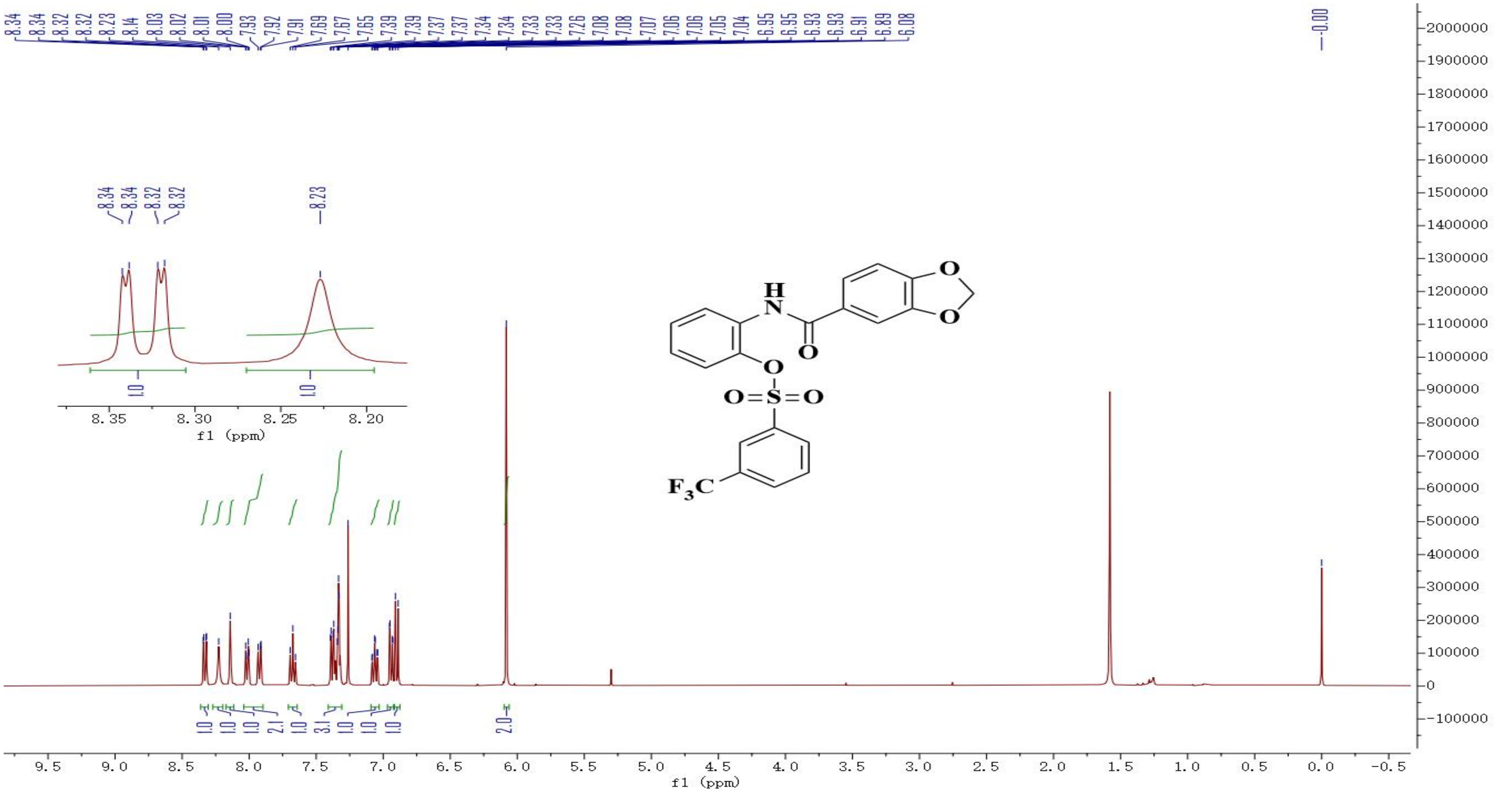

^{1}H NMR of Compound **4t**

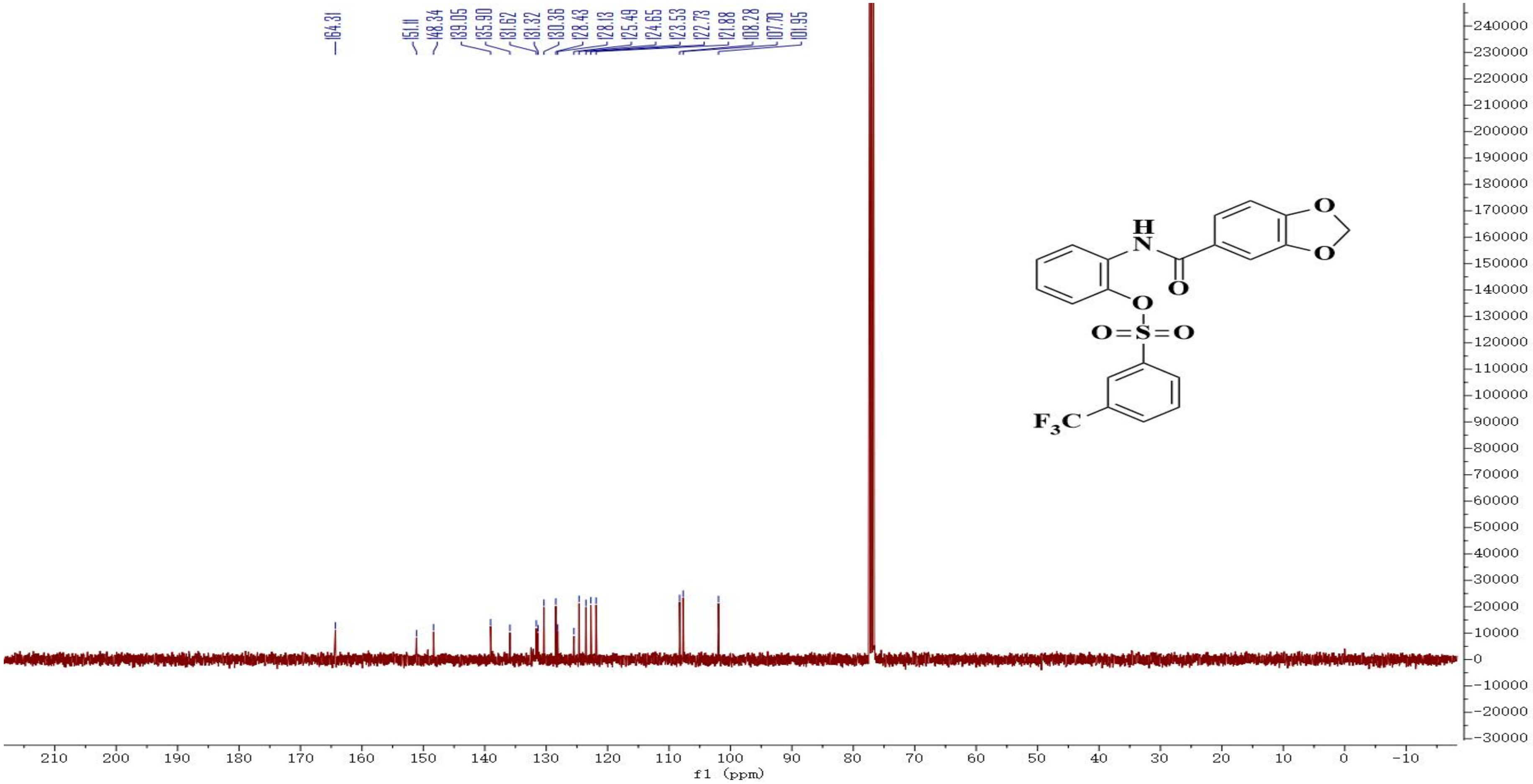

^{13}C NMR of Compound **4t**

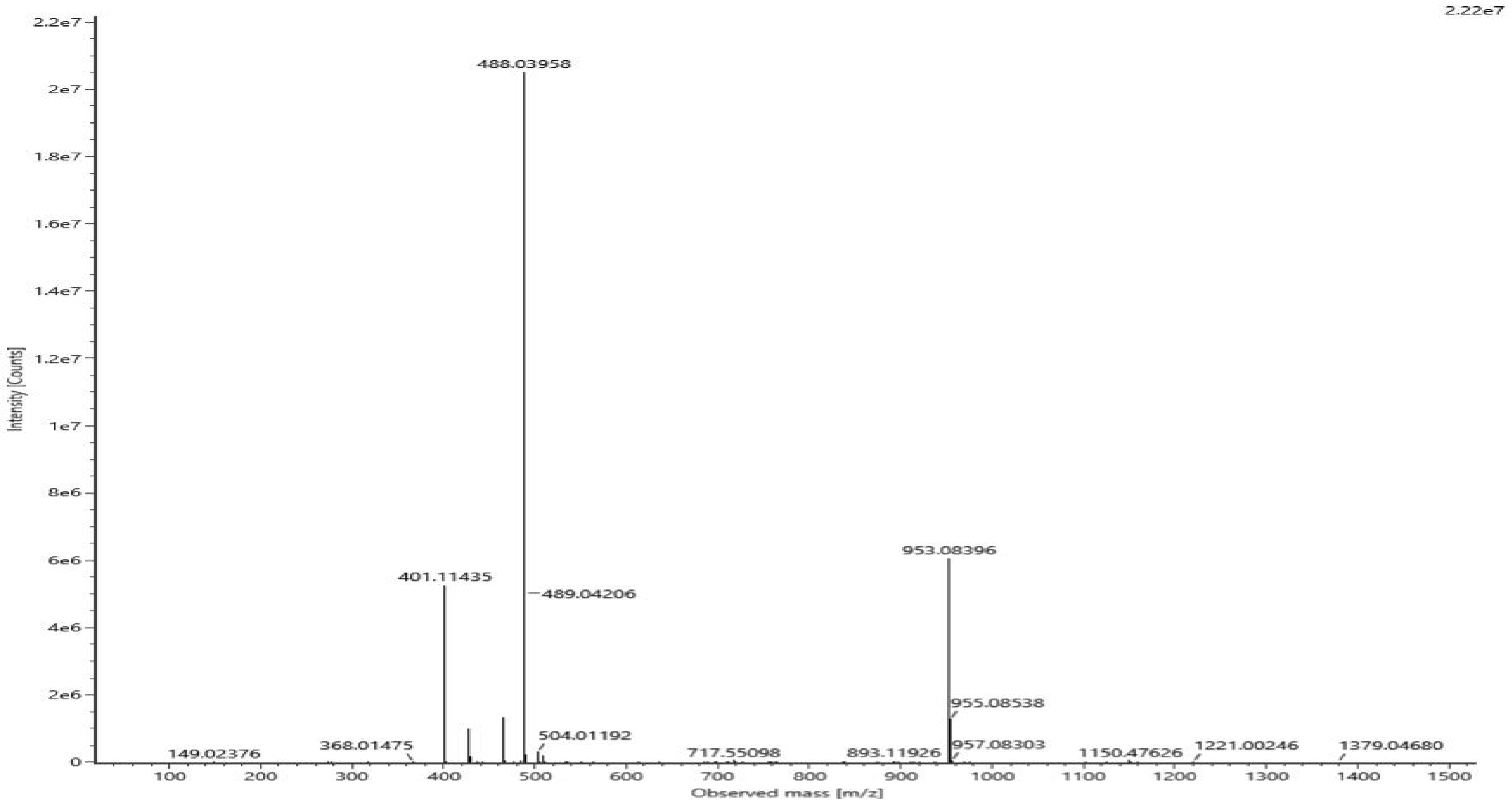

HRMS of Compound **4t**

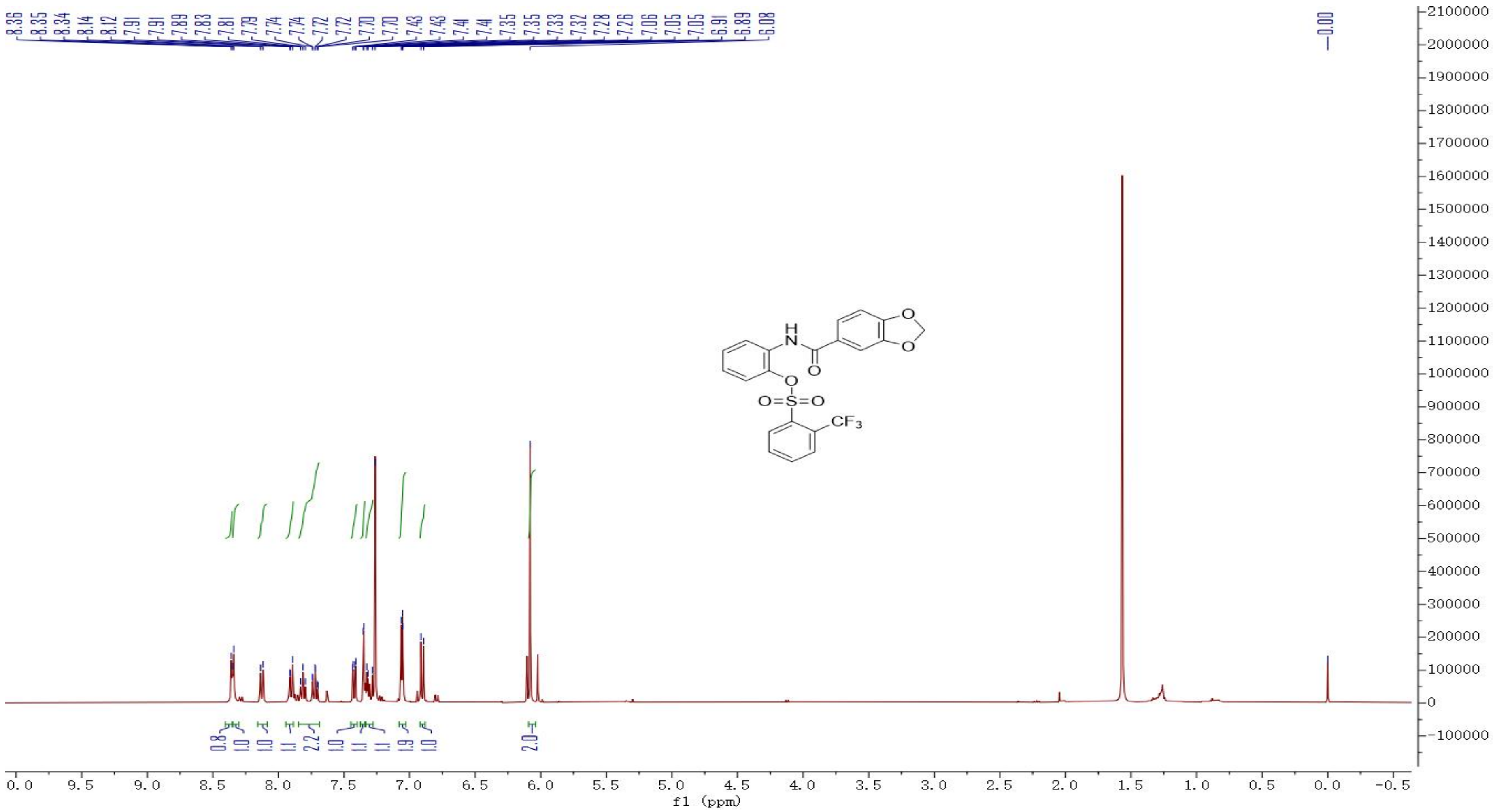

^{1}H NMR of Compound **4u**

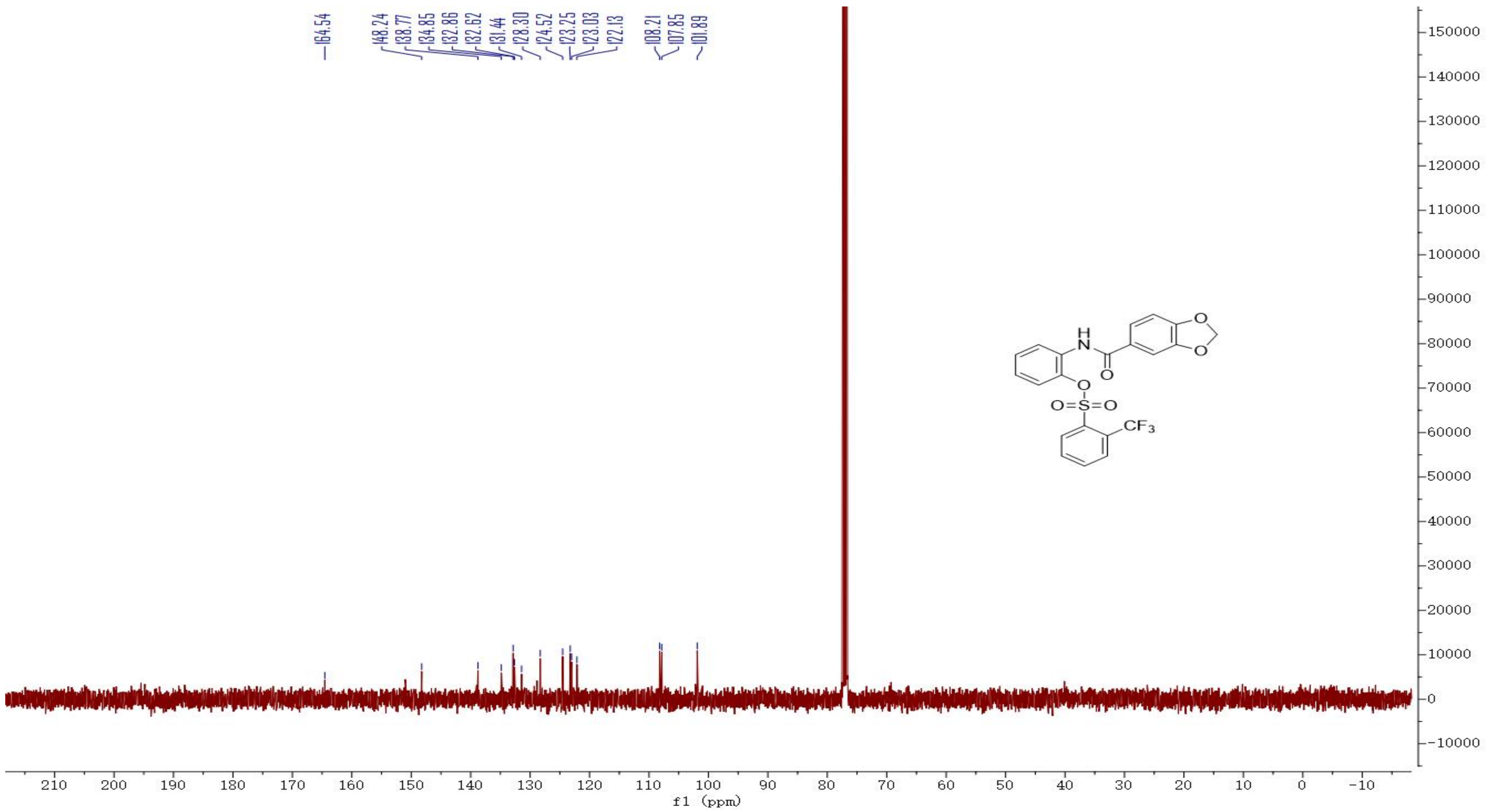

^{13}C NMR of Compound **4u**

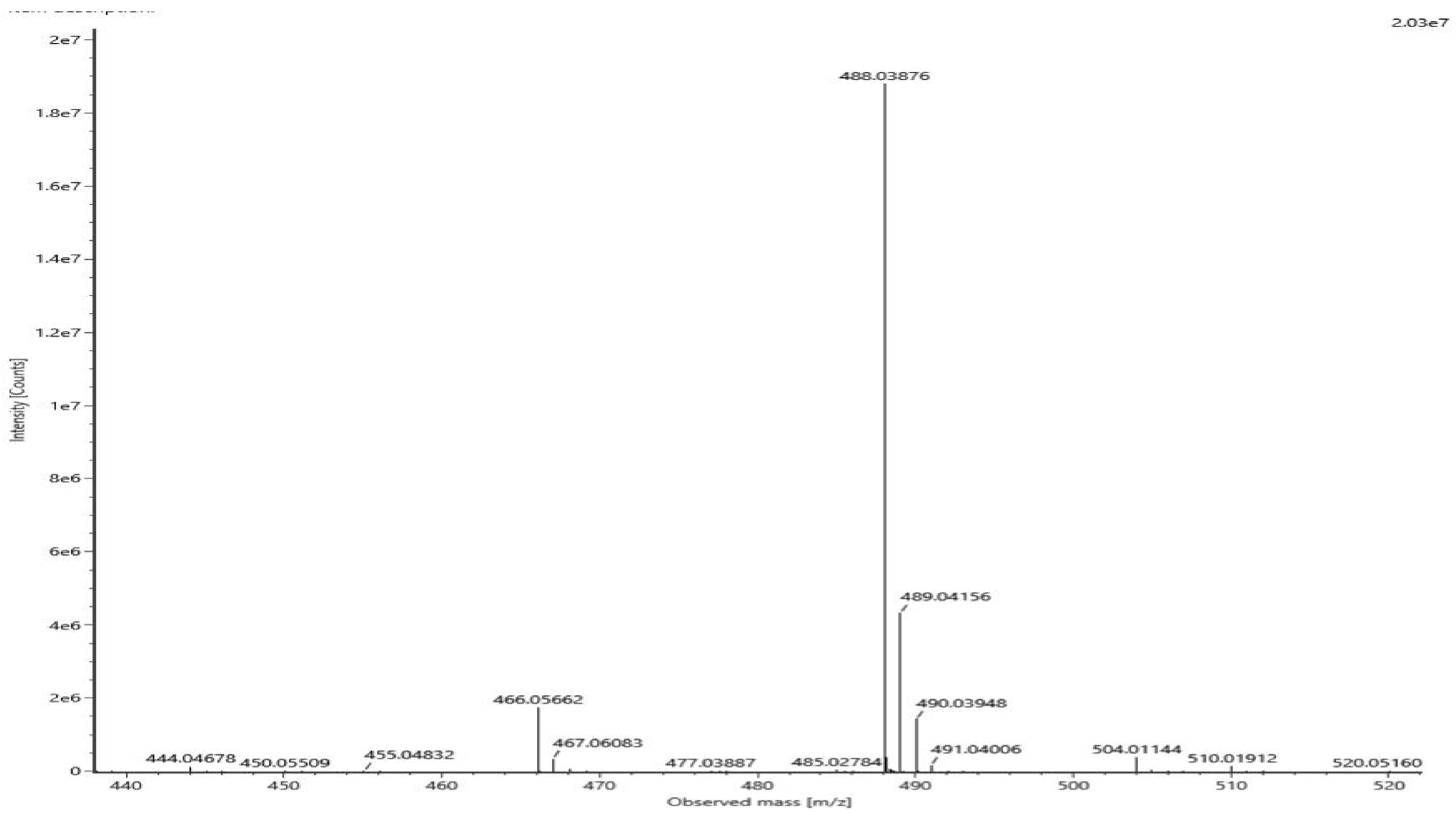

HRMS of Compound **4u**

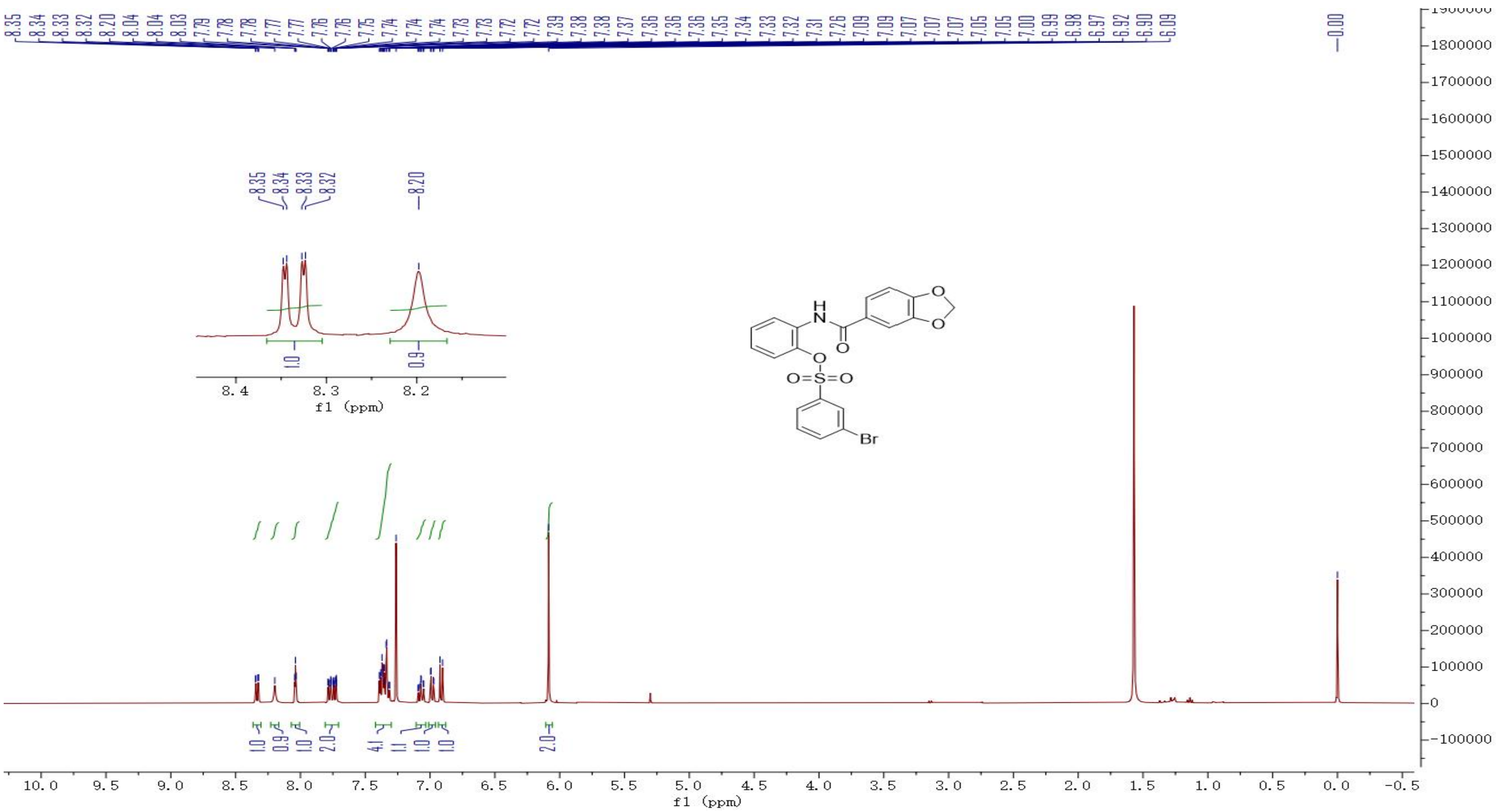

^{1}H NMR of Compound **4v**

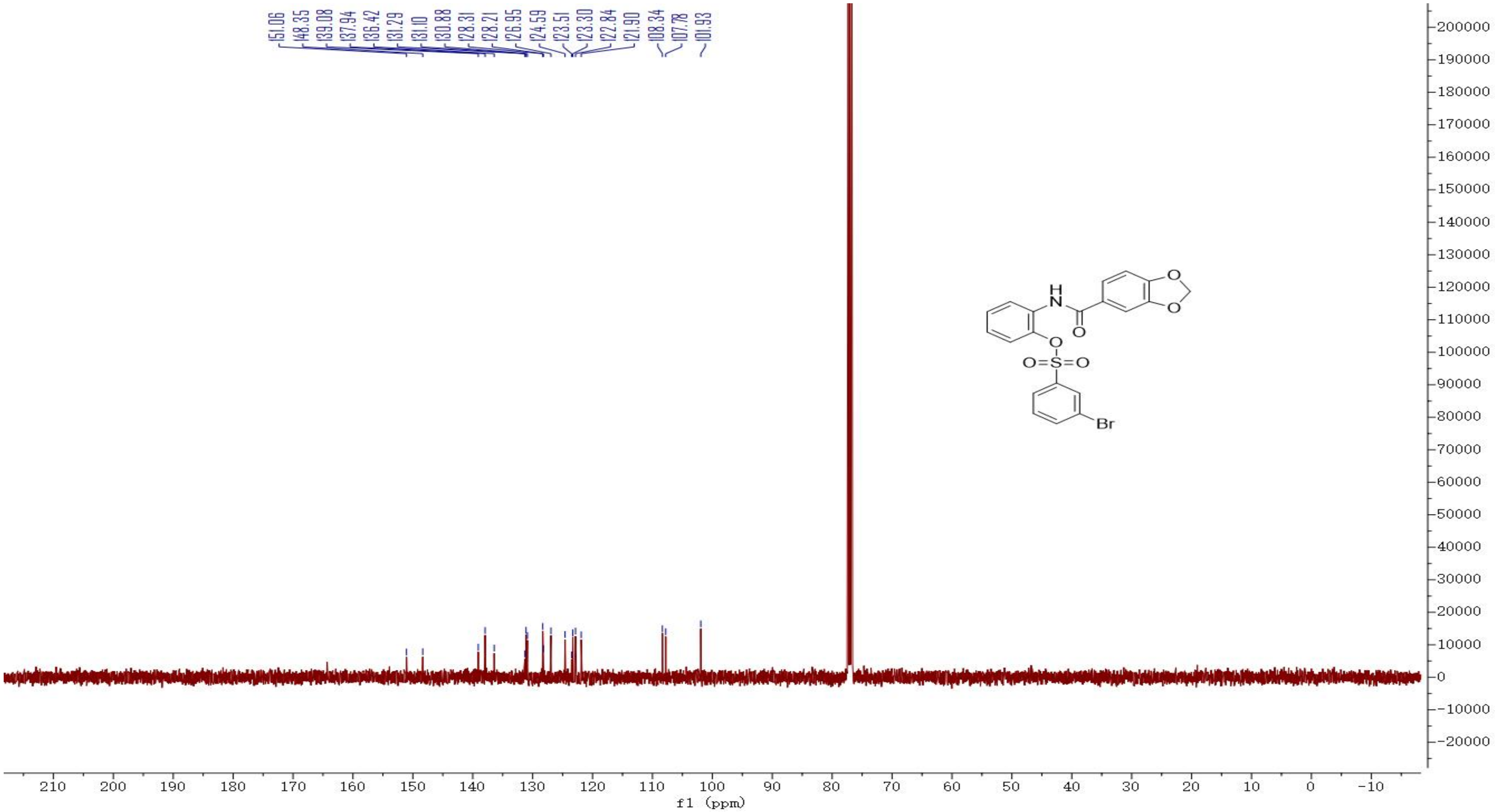

^{13}C NMR of Compound **4v**

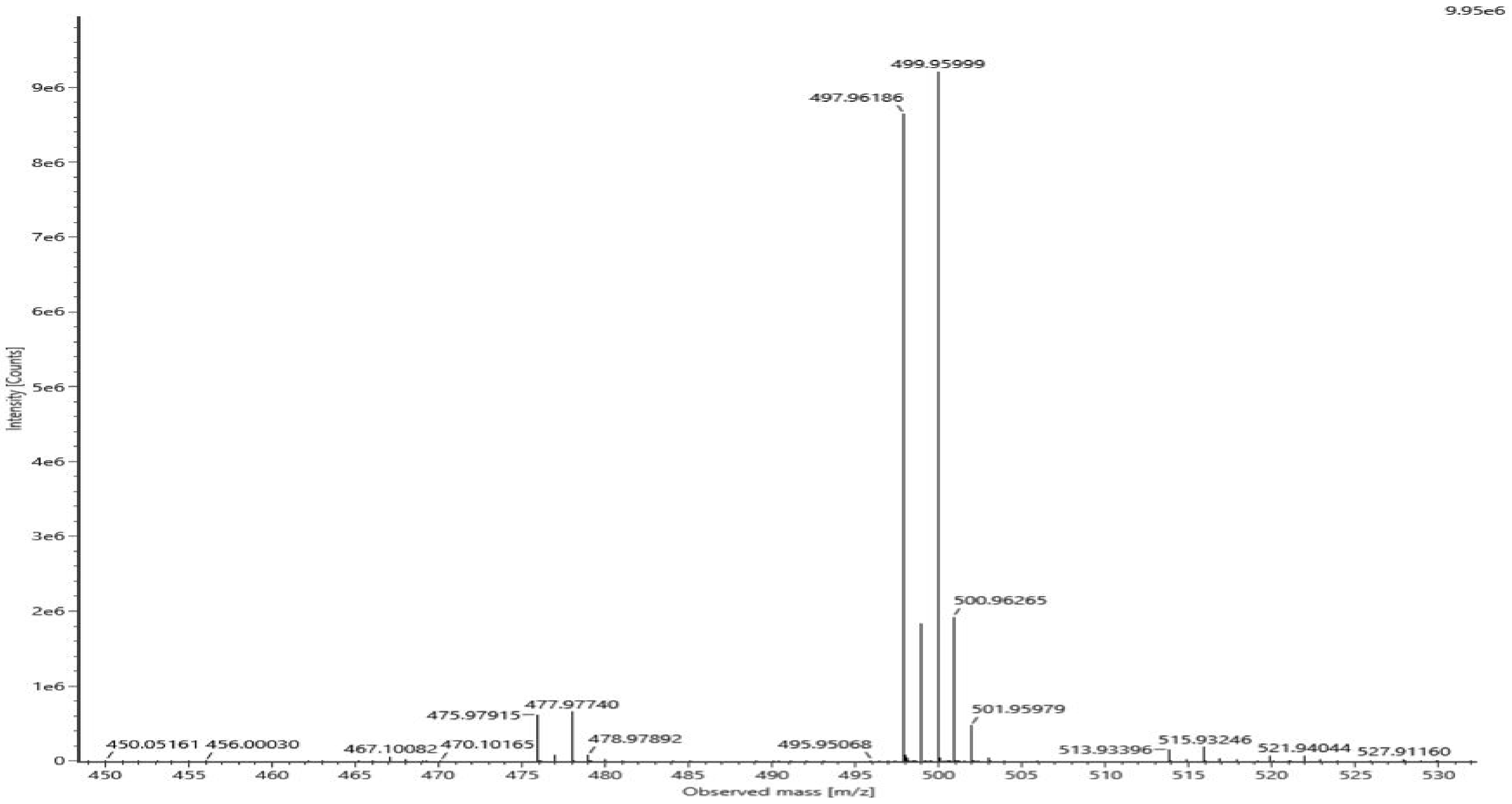

HRMS of Compound **4v**

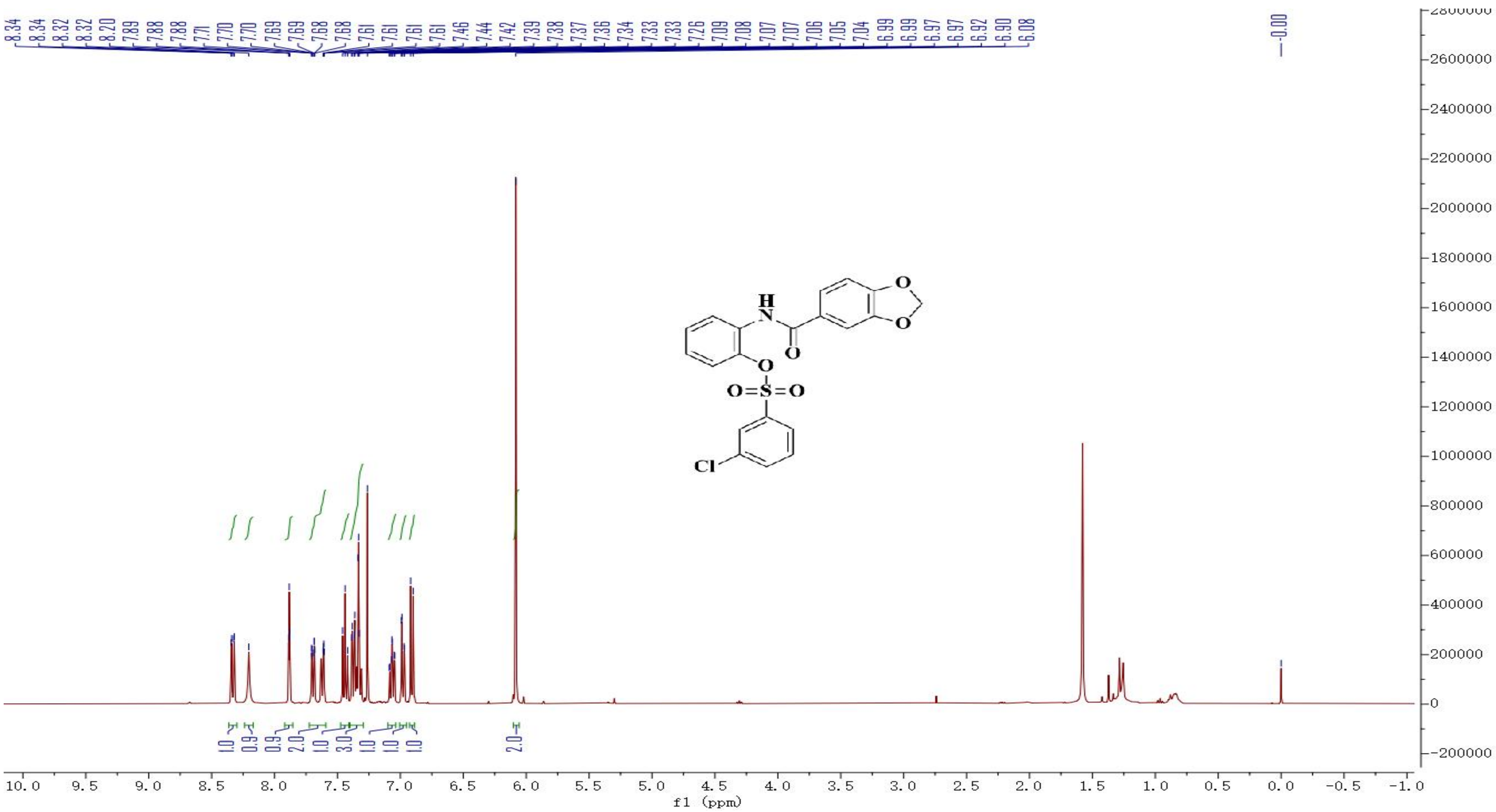

^{1}H NMR of Compound **4w**

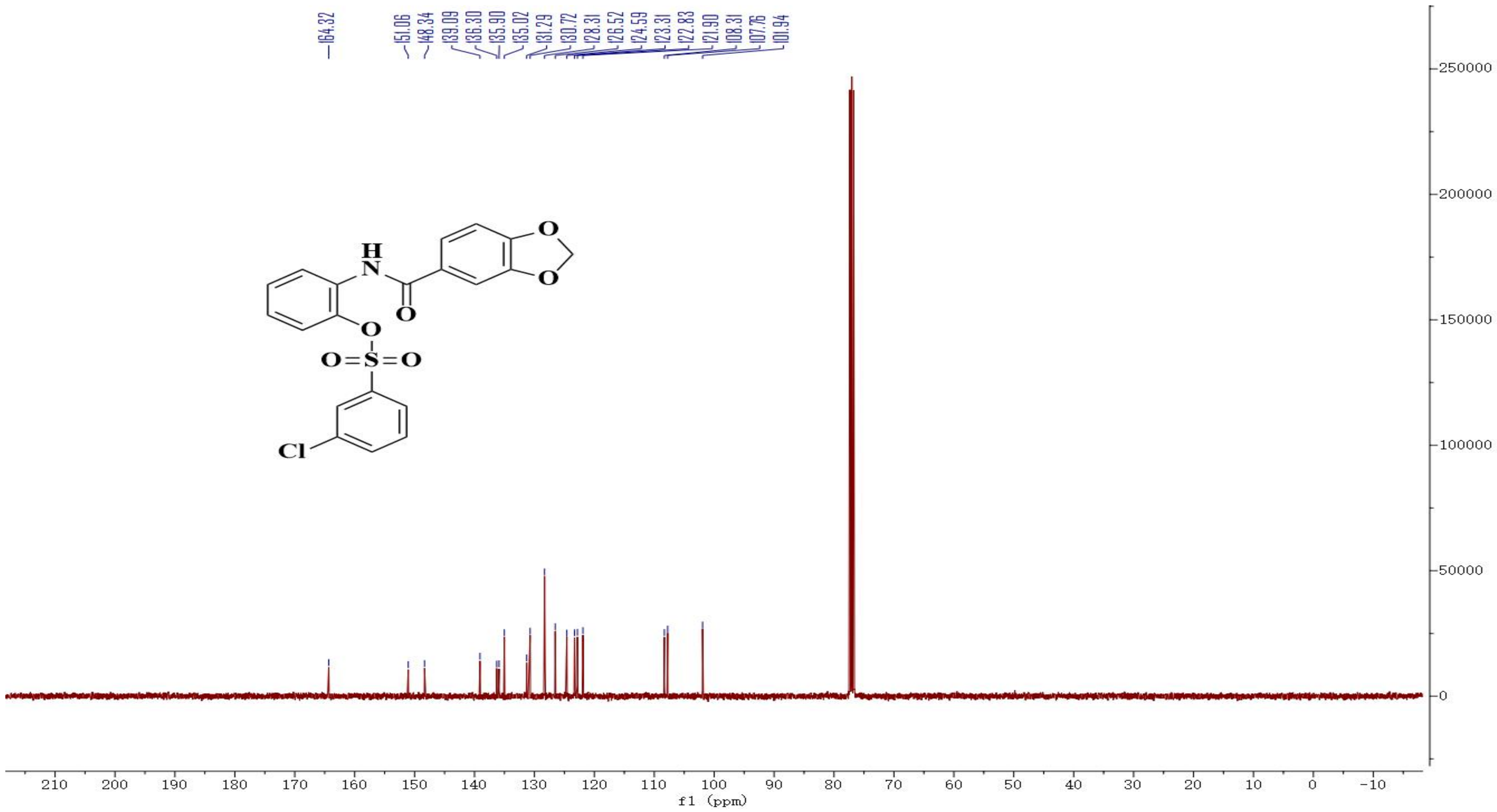

^{13}C NMR of Compound **4w**

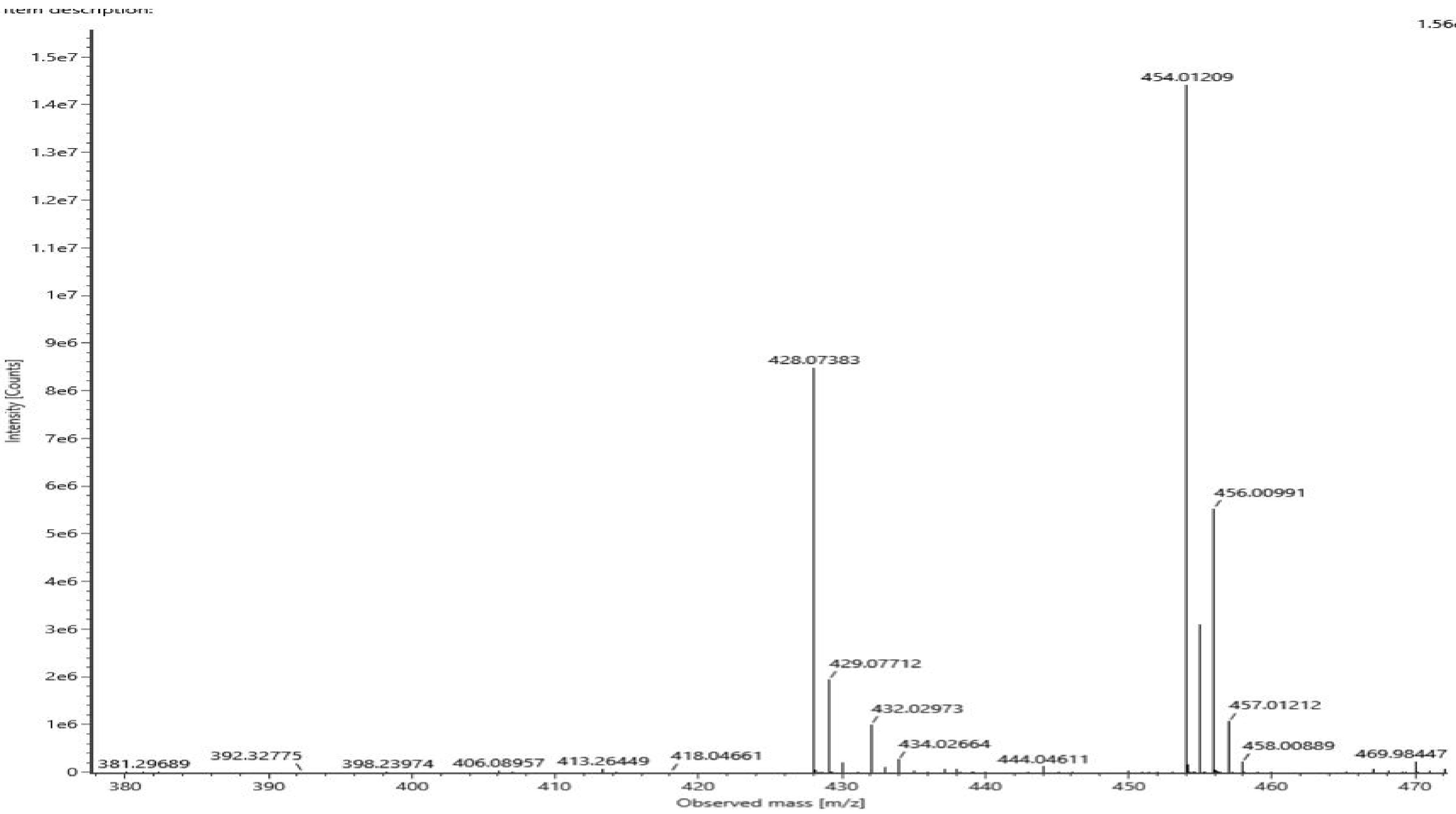

HRMS of Compound **4w**

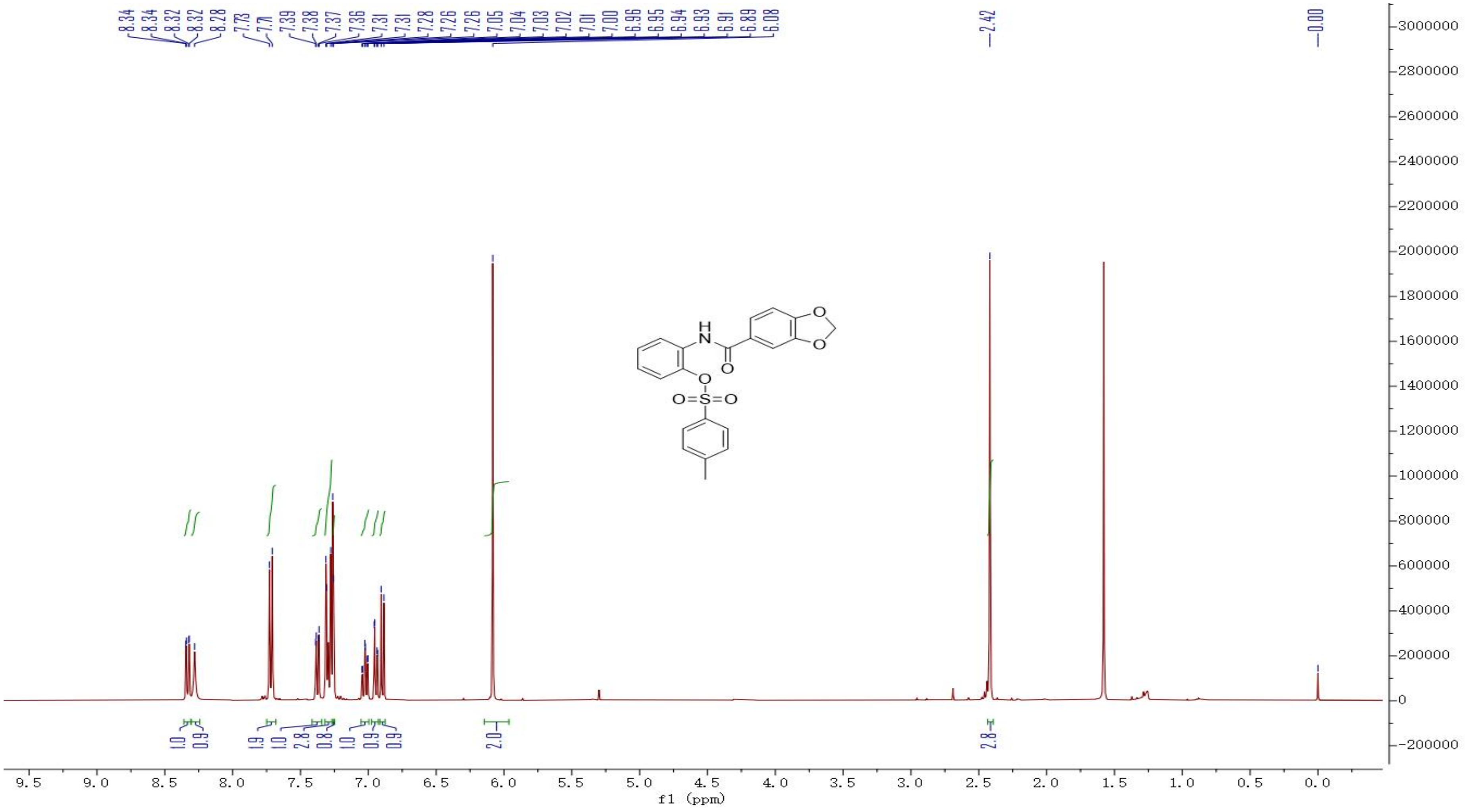

^{1}H NMR of Compound **4x**

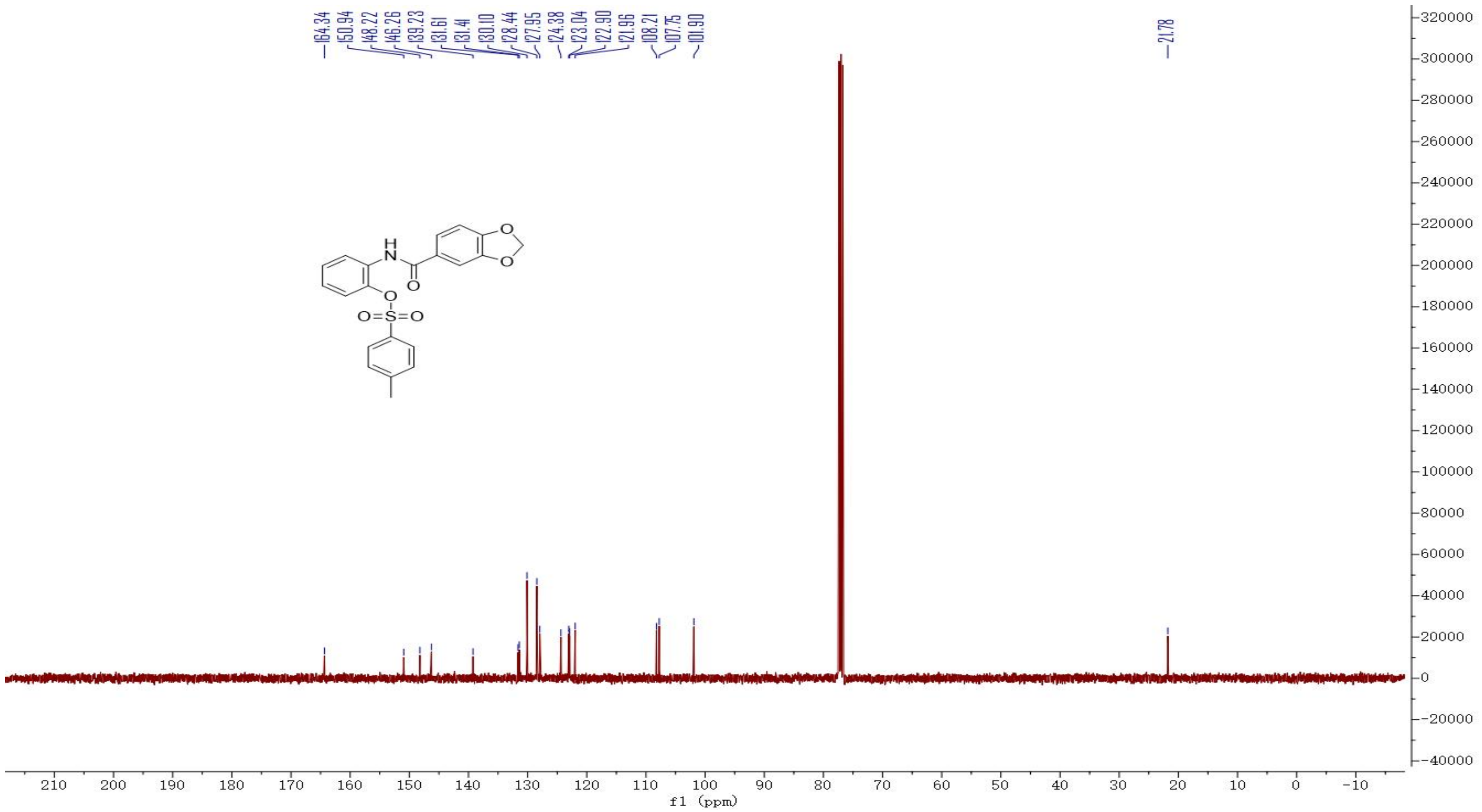

^{13}C NMR of Compound **4x**

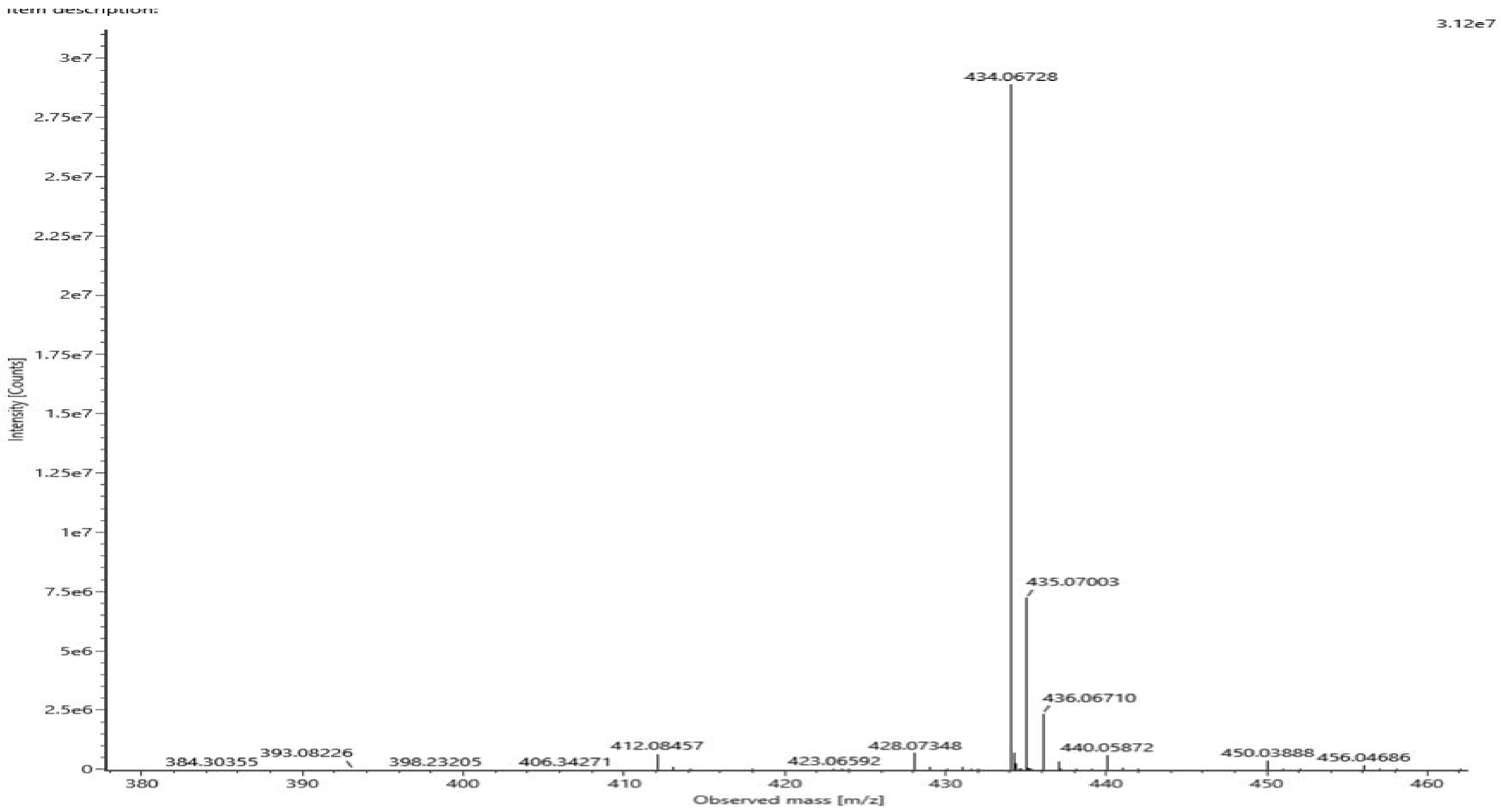

HRMS of Compound **4x**
